# Fueling Open-Source Drug Discovery: 177 Small-Molecule Leads against Tuberculosis

**DOI:** 10.1002/cmdc.201200428

**Published:** 2013-01-10

**Authors:** Lluís Ballell, Robert H Bates, Rob J Young, Daniel Alvarez-Gomez, Emilio Alvarez-Ruiz, Vanessa Barroso, Delia Blanco, Benigno Crespo, Jaime Escribano, Rubén González, Sonia Lozano, Sophie Huss, Angel Santos-Villarejo, José Julio Martín-Plaza, Alfonso Mendoza, María José Rebollo-Lopez, Modesto Remuiñan-Blanco, José Luis Lavandera, Esther Pérez-Herran, Francisco Javier Gamo-Benito, José Francisco García-Bustos, David Barros, Julia P Castro, Nicholas Cammack

**Affiliations:** [a] Tres Cantos Medicines Development Campus (TCMDC), GlaxoSmithKline (GSK)Severo Ochoa 2, Tres Cantos, Madrid (Spain); [b] CSC Medicinal Chemistry, Medicines Research Centre, GlaxoSmithKline (GSK)Stevenage, Hertfordshire, SG1 2NY (UK)

**Keywords:** drug discovery, high-throughput screening, open innovation, tuberculosis

## Abstract

With the aim of fuelling open-source, translational, early-stage drug discovery activities, the results of the recently completed antimycobacterial phenotypic screening campaign against *Mycobacterium bovis* BCG with hit confirmation in *M. tuberculosis* H37Rv were made publicly accessible. A set of 177 potent non-cytotoxic H37Rv hits was identified and will be made available to maximize the potential impact of the compounds toward a chemical genetics/proteomics exercise, while at the same time providing a plethora of potential starting points for new synthetic lead-generation activities. Two additional drug-discovery-relevant datasets are included: a) a drug-like property analysis reflecting the latest lead-like guidelines and b) an early lead-generation package of the most promising hits within the clusters identified.

## Introduction

Despite available treatments for tuberculosis (TB), the threat this disease represents is still a painful reality for the ten million people infected and the two million that die from TB each year.^1^ Infections by *Mycobacterium tuberculosis*, the causative bacterial agent of TB, represents an escalating threat for global health with the increased prevalence of multi- and extensively drug-resistant (MDR and XDR, respectively) TB. Mortality rates for XDR TB can approach 100 % of those infected.^2^ In some countries, MDR/XDR strains can account for up to 22 % of infections.^3^

While these numbers are partly attributable to the misuse of current antitubercular agents, they are also a direct consequence of the nature of the treatment: a combination of at least three different drugs that must be taken for six months or longer. Owing to side effects and the length of treatment, patients often partly or completely drop off therapy, with a consequent rise in drug-resistant strains and cases of infection relapse. This increased prevalence of resistant TB has led the World Health Organization (WHO) to call for the widespread implementation of *directly observed treatment short course* (DOTS), in which treatment compliance is monitored by healthcare workers. Although this approach has been successful where appropriately implemented, achieving an 84 % cure rate according to the latest WHO 2011 TB report,^1^ the development of a new drug regimen for the treatment of TB could still be the most cost-effective way of tackling the pandemic. Specifically, any new drug should ideally be able to shorten the duration of treatment, avoid significant drug–drug interactions with current regimens, treat MDR and XDR TB patients (via a new mode of action), and be cost competitive with current drugs.

Various reasons are behind the lack of new medicines for the treatment of TB and other neglected diseases over the last 40 years, but most of them are generally related to a lack of critical mass in terms of funding, R & D capacity, and commercial interest. With the aim of contributing to the reversal of this trend, researchers at GlaxoSmithKline (GSK) and others^4^ recently decided to explore new collaborative models of conducting early-stage TB drug discovery research to help mitigate current limitations. One such strategy is the translation of the “open source” philosophy,^5^ which was recently manifested in the publication of several thousand small molecules from our corporate compound collection that showed significant in vitro potency against the malaria parasite *Plasmodium falciparum*.^6^

It is with the aim of stimulating similar community-based research efforts for novel TB therapeutics that we make the results of a phenotypic screening campaign against *M. bovis* BCG with hit confirmation in *M. tuberculosis* H37Rv publicly available. These datasets are intended to serve a dual purpose: to maximize the potential impact of the compounds toward a chemical genetics/proteomics exercise, while simultaneously providing many potential new starting points for synthetic lead-generation activities.

### Target-based versus phenotypic screening in infectious diseases

Although target-based approaches are widely used in drug discovery, questions have been raised about the efficiency of this approach given the very high attrition rates that these projects have historically shown in the anti-infectives field.^7^, ^8^ This situation becomes particularly alarming when considering the very limited number of validated targets for TB drug discovery. Furthermore, the state of affairs is a symptom of wider inconsistencies between results obtained with animal models of infection and their translation to clinical therapeutic value in humans. Overall, we describe this situation as a 4M disconnect, that is, a lack of translation between data generated at the **m**olecular level (target), the **M**IC (minimum inhibitory concentration), in **m**ice (in vivo animal efficacy models)^9^, ^10^ and in **m**an. To address the early disconnect between the molecular and MIC datasets, the present trend is to turn to whole-cell screens, in which compounds are identified based on their antibacterial activity. To facilitate advancement of cell-based hits, it is important to identify the corresponding cellular targets. New genetic, genomic, and proteomic tools are now available that increase the feasibility of this process for new antitubercular agents.^11^ Compounds identified in whole-cell screens fulfill a double function: 1) they provide lead structures for further optimization within the drug development progression sequence, and 2) they can be exploited as tools to identify new targets. Notably, cell-based hits already fulfill some important criteria, including permeability issues (very troublesome in TB due to the impervious nature of the mycobacterial cell wall) and, given the progression criteria in the high-throughput screen, higher activity against mycobacteria than mammalian cells. Thus, they provide suitable chemical and biological starting points. Of course, lead optimization projects in which the key driving factor is phenotypic antitubercular activity can present medicinal chemists with significant challenges in terms of understanding and rationalizing structure–activity relationships (SAR), as changes in penetration of the compound may also modulate activity in addition to producing changes in target engagement.

## Results and Discussion

### *M. bovis* BCG as a TB surrogate

To evaluate the feasibility of performing a high-throughput screening (HTS) campaign with GSK′s corporate compound collection (>2×10^6^ chemical entities) against mycobacteria while avoiding the implicit limitations related to manually manipulating a large number of microtiter plates and large culture volumes of infective material within a biosafety level (BSL) 3 environment, we decided to use *M. bovis* BCG as an *M. tuberculosis* surrogate for early drug screening purposes. *M. bovis* BCG can be manipulated safely within a BSL2 environment, and its genome is >99 % identical to that of *M. tuberculosis* H37Rv.^12^

To challenge the predictive value of BCG as an *M. tuberculosis* surrogate, we selected a GSK compound collection subset (Core02) of ∼20 000 compounds. The compounds belonging to this subset are characterized by good cell membrane permeability (measured values), availability within GSK stocks, and fall clearly within pre-established drug-like parameters.^13^

Compounds were assayed against both *M. bovis* BCG and *M. tuberculosis* H37Rv (referred to herein as BCG and H37Rv, respectively) in two independent experiments at a single 10 μm concentration. Promisingly, similar hit rates were found in both experiments: 127 primary hits were identified as inhibitors of BCG growth and 112 hits as inhibitors of H37Rv. Retesting both sets of hits confirmed 88 of the compounds detected in the BCG screen (69 %) and 73 of the primary hits from H37Rv (65 %) as active.

We then proceeded to assess the degree of cross-inhibition between the two strains, assaying all confirmed hits that inhibit BCG against H37Rv, and vice versa. To maximize the information gained from this exercise, the cross-screening was performed at two different concentrations: the standard 10 μm used in the primary screen and 25 μm. This second activity point would help us spot compounds that have cross-inhibition activity, but at lower levels of potency. Only 55 % of the BCG positives were able to inhibit H37Rv at 10 μm (48 out of 88) although this percentage increased to 86 % (76 out of 88) when tested at 25 μm. Most of the compounds selected as H37Rv inhibitors were able to inhibit BCG growth (67 out of 73) when assayed at 10 μm. When the assay compound concentration was raised to 25 μm, the degree of cross-activity increased even further to 97 % (71 out of 73).

Altogether, these results prompted us to consider the use of the BCG surrogate as a feasible compromise for detecting H37Rv inhibitors. This evidence came with the clear realization that any BCG-based screening campaign was bound to generate a significant number of BCG-specific compounds that, although interesting for the basic understanding of the biology of mycobacteria, would be devoid of potential from a drug-discovery standpoint.

### HTS BCG results

The initial HTS BCG primary hit list (see Supporting Information for more details) was narrowed after the application of a number of similarity and physicochemical property filters. First, a GSK internally developed soft filter algorithm was applied to select a smaller subset of hits, making sure that every compound that was not selected had a neighbor within a given Daylight Tanimoto similarity of 0.7.^14^ In that process all hits showing >90 % inhibition at 10 μm were included. Second, a physicochemical properties hard filter was applied to compounds with calculated log *P*>6 and *M*_r_>600 Da. These relatively relaxed initial drug-like property considerations were included considering recent discussions with regard to the possible specific physicochemical makeup of antitubercular agents.^15^, ^16^ Third, in the search for new chemical diversity, known antibacterials identified as positives during the campaign were also discarded. Fourth, structures containing reactive functional groups were also eliminated. An overall description of that process can be found in Figure 2 below.

This selection resulted in a final list of 3509 compounds that were progressed to dose–response studies. According to the dose–response results, 309 compounds exhibited inhibition of BCG growth with IC_50_>10 μm, 2694 compounds with IC_50_ between 1 and 10 μm, 215 compounds with IC_50_ between 100 nm and 1 μm, and 10 compounds with IC_50_ between 10 and 100 nm. A total of 281 compounds displayed no measurable IC_50_ values against *M. bovis* BCG growth at the tested concentrations.

### Determination of cytotoxicity of HTS hits

With the aim of establishing an initial indicator of the possible therapeutic index for the hit structures, the HepG2 cytotoxicity of each antimycobacterial hit was evaluated. From the dose–response results, 1471 compounds displayed IC_50_ values <10 μm, 304 compounds displayed IC_50_ values between 1 and 10 μm, 20 compounds between 100 nm and 1 μm, 27 compounds between 10 and 100 nm, and 25 compounds between 1 and 10 nm. The remaining 1662 compounds displayed no detectable cytotoxic effects.

**Figure 1:**
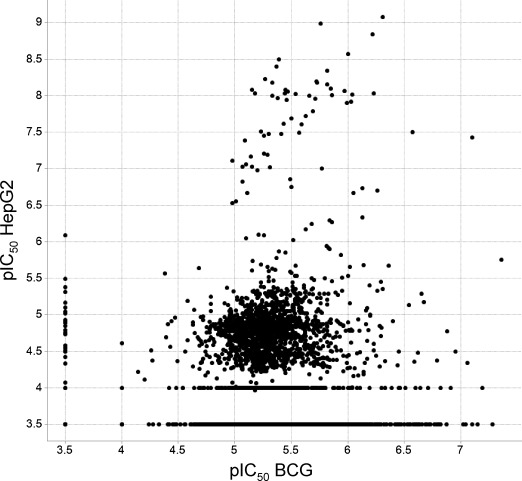
Plot of pIC_50_ BCG versus pIC_50_ HepG2: compounds were ranked according to therapeutic index [T.I.=(IC_50_ HepG2)/(IC_50_ BCG)]. According to the criteria established (T.I.>50), 960 compounds were selected for evaluation in H37Rv.

**Figure 2:**
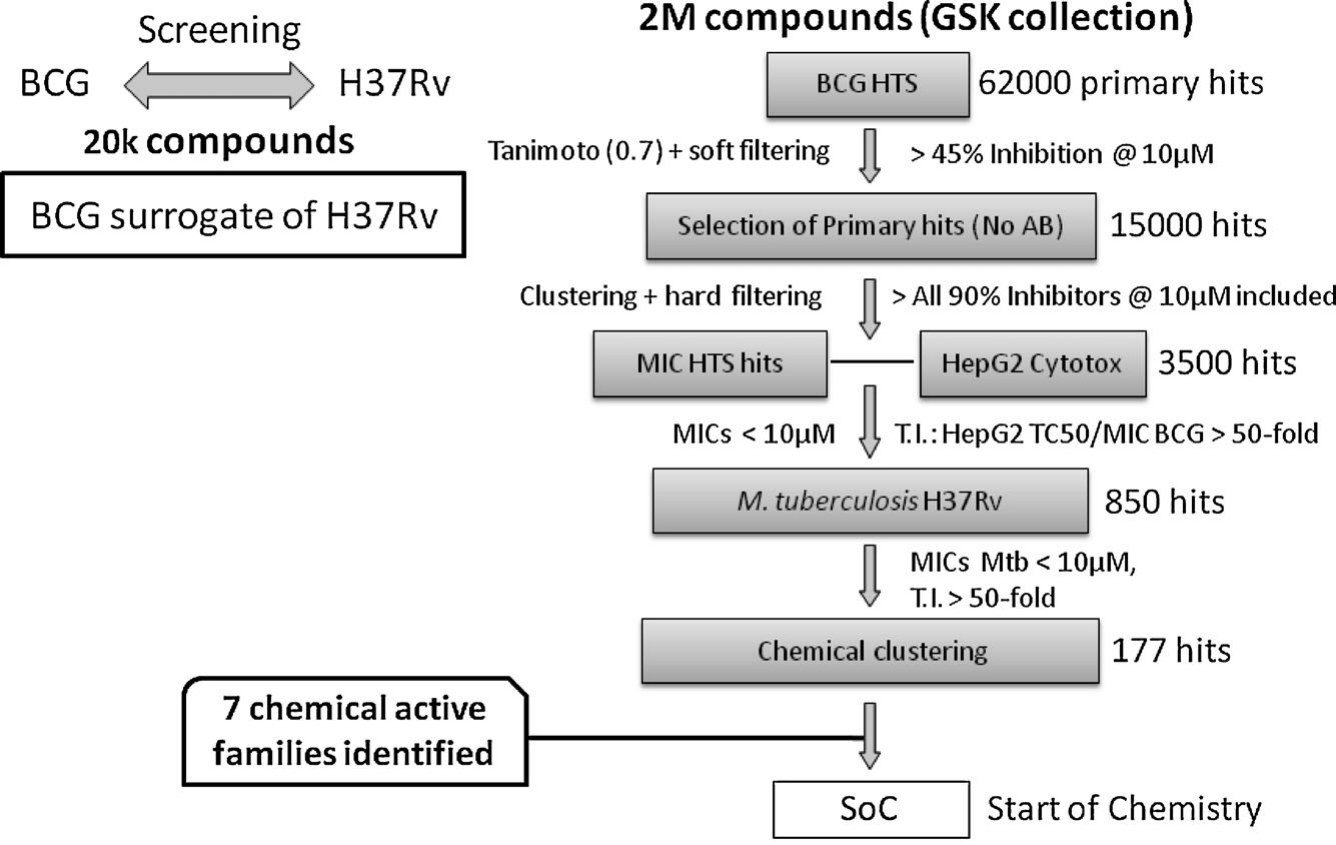
HTS progression cascade leading to 177 confirmed H37Rv-positive compounds.

After combining cytotoxicity and antimycobacterial activity, a total of 960 compounds displayed a therapeutic index [T.I.=(IC_50_ HepG2)/(IC_50_ BCG)] >50 (Figure 1). This list of 960 compounds was further refined by establishing the BCG MIC threshold at MIC<10 μm. These criteria yielded 777 compounds that were further progressed to MIC determination against H37Rv under standard 7H9 medium growth conditions, resulting in 177 positives showing an MIC against H37Rv of <10 μm and a therapeutic index of (HepG2 IC_50_/MIC)>50 (Figure 2). Reevaluation of the 777 BCG-positive list in H37Rv under alternative carbon source and anti-replicating conditions is currently ongoing and will be disclosed elsewhere.

**Figure 3:**
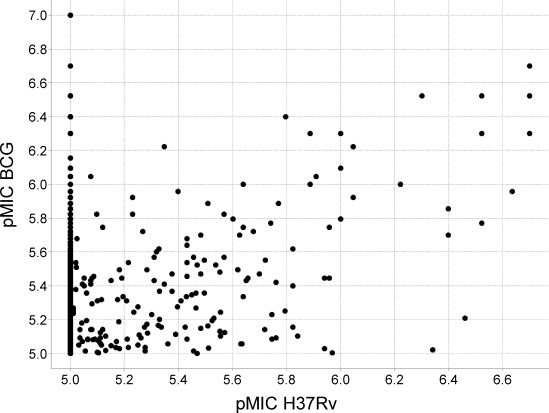
Plot of pMIC H37Rv versus pMIC BCG: comparison of the H37Rv versus BCG activities of all active compounds from the BCG screen. Although a large number of BCG-selective compounds were found (vertical cluster at left, pMIC H37Rv=5.0), many hits displayed good activities in both species.

**Figure 4:**
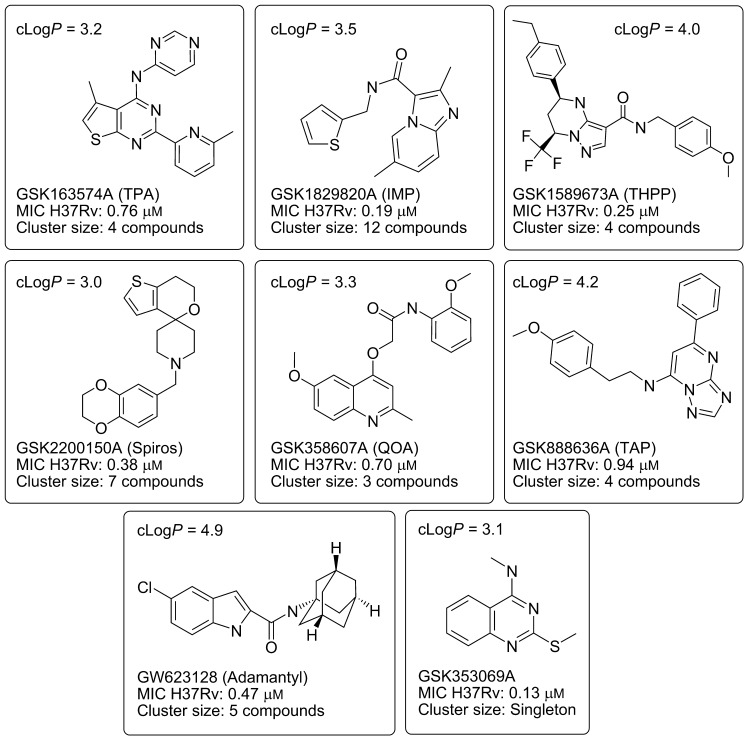
Most promising families from the HTS campaign: seven chemical families and one potent singleton selected from the 177 H37Rv hits for complete biological profiling. Full data are listed in Table 1.

In agreement with recently published data by Novartis,^17^ a high level of attrition was observed between the selected BCG compound set (777 compounds, Supporting Information) and the final H37Rv list (177 compounds, Supporting Information) (Figure 3). Although this compound progression flow did provide a large number of BCG-selective hits, we were confident that no *M. bovis* BCG positives from this screen with potential for *M. tuberculosis* inhibition were left out of the H37Rv MIC determination. Visual clustering of this final selection of 177 *M. tuberculosis* positives provided a number of highly potent structural clusters (Figure 4 and Supporting Information).

### Drug-like parameters

**Figure 5:**
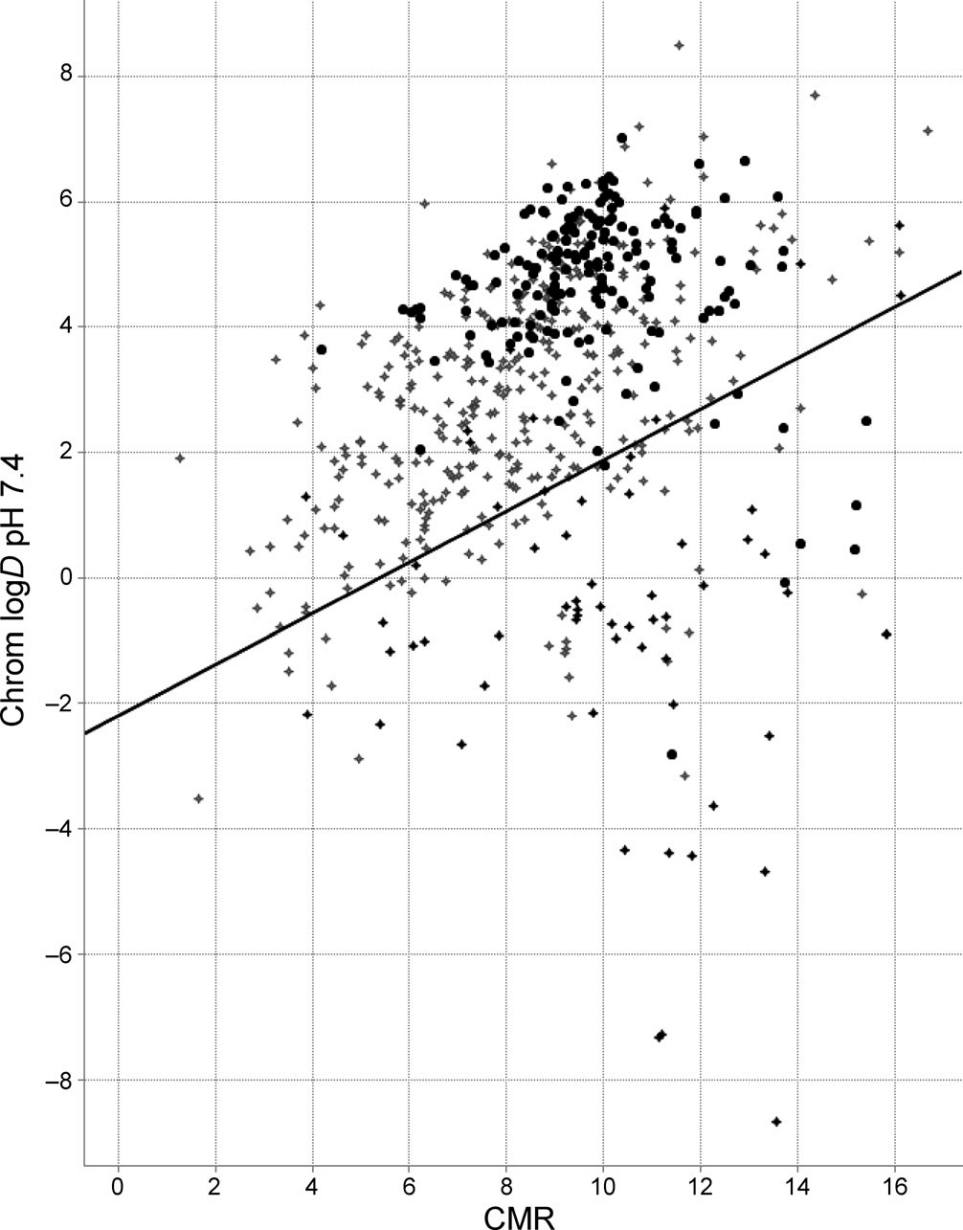
Plot of calculated chromatographic log *D*_7.4_ versus calculated molar refraction (CMR). Grey crosses represent marketed drugs with >30 % oral bioavailability, black crosses <30 % oral bioavailability, and the “TB set” as black circles. The line represents a discriminator between likely good and bad permeability. The chromatographic log *D* scale gives values approximately two units higher than the traditional distribution values assessed in octanol/water.

In assembling this set of compounds, attention was paid again to physical properties to help further triage and select the most attractive chemical starting points for later optimization. A good indication of the quality of the compounds in the set is illustrated in Figure 5, in which the lipophilicity of the set is plotted versus size, as computed by chromatographic log *D* pH 7.4 (log *D*_7.4_) and calculated molar refraction, respectively.^18^ The compounds reside comfortably within the bounds occupied by marketed drugs, if generally toward the more lipophilic extreme. Measured solubility data (see Supporting Information) were available for most compounds and were generally within acceptable levels; this might have been expected given the distribution of lipophilicity and aromatic rings.^19^, ^20^ In any optimization program, attention to decreasing lipophilicity and/or minimizing aromatic ring count would be a sensible approach with some of the compounds, but the majority present attractive starting points for lead optimization.

### Hit characterization

To further elucidate the lead progression potential of the antitubercular positive hit list, a number of chemical clusters were selected based on two criteria: a) H37Rv MIC<1 μm and b) the number of structural analogues represented within the list. At this point, other less potent structural classes and singletons were deprioritized, yet further evaluation of these compounds remains ongoing. This exercise yielded seven chemical clusters, representatives of which (one or two compounds) were selected for further characterization. This secondary biological and physical profiling package consisted of a number of assays representing important compound progression and selection criteria in early-stage antitubercular drug discovery, including MIC determination against various *M. tuberculosis* strains, characterization against intracellular growth (see Supporting Information for protocol details), activity profiling against an internal panel of eight different bacteria for early assessment of the potential for wide-spectrum activity, measured solubility (see Supporting Information for protocol), evaluation of cytotoxicity, artificial membrane permeability studies (see Supporting Information for protocol), cytochrome P450 (CYP) inhibition profiling against the most notorious drug-discovery-relevant isoforms,^21^ and early in vitro metabolic studies to establish the stability of the hit structures in mouse and human microsomal fractions (Cl_int_ and *t*_1/2_ values; see Supporting Information for protocol). All data were collected and are listed in Table 1.

**Table 1:** Complete biological profile of selected hit compounds.^[a]^

REGNO	Family					MIC [μm]^[b]^							Ctox.	Solu.	Perm.			CYP				Mouse		Human
		H37Rv	I-BCG	BCG	*Efa* I	*Efm* X7501	*Hin* H128	*Hin* H128 Acr A−	*Mca* 1502	*Spn* 1629	*Eco* 3	Spy 1308007P				2D6	2C9	2C19	3A4VR	3A4VG	Cl_int_	*t*_1/2_	Cl_int_	*t*_1/2_
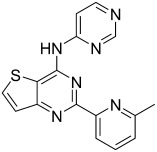 GSK153890A	TPA	0.47	>10	0.3	>64	16	8	0.5	1	>64	>64	>64	31.4	277	480	42	26	31		17	19.5	<3	3.6	22
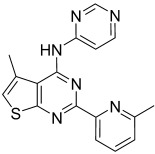 GSK163574A	TPA	0.76	>10	0.3	16	8	16	0.25	0.5	16	>64	16	>25 (47 %)	23	140	3.5	17	0.7	32	76	20.8	<3	7.2	11.5
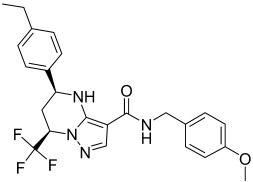 GSK1589673A	THPP	0.25	0.5	2.7	>64	64	64	16	8	64	>64	64	38	0	130	7.6	4.4	10	>50	5.0	1.9	>30	1.8	>30
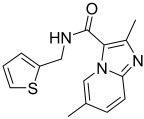 GSK1829820A	IMP	0.19	>10	0.5	>64	>64	>64	>64	>64	>64	>64	>64	>25	339	560	3.8	7.6	25	>50	9.8	>30	<3	3.9	21
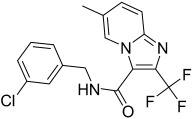 GSK1829731A	IMP	0.19	>10	ND^[c]^	64	>64	64	64	64	64	>64	16	>50	9	1300	1.9	3.1	14	43	3.5	>30	<3	>30	<3
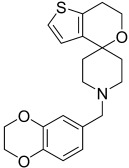 GSK2200150A	Spiros	0.38	0.4	1.6	64	64	64	64	32	64	>64	16	>50 (48 %)	≥260	500	0.5	48	20	>50	26	34.9	<3	25.1	5.2
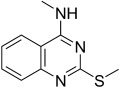 GSK353069A	singleton	0.13	>10	0.3	>64	64	>64	>64	>64	>64	>64	>64	>50	≥443	600	>50	>50	22	>50	17	>30	<3	9.7	6.7
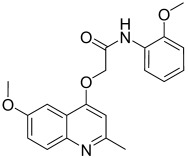 GSK358607A	QOA	0.70	>10	1.4	64	>64	64	32	32	64	>64	16	>50	38	120	32	0.6	2.0	>50	3.4	>30	<3	5.1	16.7
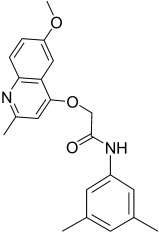 GSK749336A	QOA	0.25	>10	0.5	64	>64	64	32	32	64	>64	16	>50	32		4.6	2.8	11	16	1.9	>30	<3	4.3	17.5
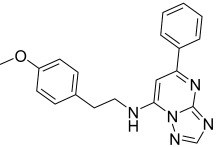 GSK888636A	TAP	0.94	>10	1	>64	64	64	64	32	>64	>64	16	>25	14	350	>50	1.6	<001	>50	3.3	9.8	7.3	3	24.8
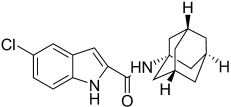 GW623128	Adamantyl	0.47	0.06	0.8	>64	>64	>64	>64	>64	>64	>64	>64	>50	<1	470	>50	6.3	21	>50	20	3.3	21.9	0.9	>30
Rifampicin		0.02											>256											
Isoniazid		1.80											207											

[a] Data include MICs against *M. tuberculosis* and a variety of other bacteria species, cytotoxicity (Ctox.: IC_50_ HepG2 [μm]), in vitro profiling for solubility (Solu.: CLND [μm]) and permeability (Perm. [nm s^−1^]), cytochrome P450 inhibition (CYP [μm]), and microsomal fraction stability in mouse and human (Cl_int_ [mL min^−1^ g^−1^], *t*_1/2_ [min]); rifampicin and isoniazid were included as controls. [b] I-BCG: intracellular BCG, *Efa*: *Enterococcus faecalis*, *Efm*: *Enterococcus faecium*, *Hin*: *Haemophilus influenzae*, *Mca*: *Moraxella catarrhalis*, *Spn*: *Streptococcus pneumoniae*, *Eco*: *Escherichia coli*, and *Spy*: *Streptococcus pyogenes*. [c] Not determined.

The screening methods described above led to the selection of seven promising chemical series for complete biological profiling (see Figure 4 and Table 1). For the purposes of discussion, the seven families were given short names or acronyms that are used throughout this section: GSK153890A and GSK163574A represent the thienopyrimidine^22^ amine (TPA) family, GSK1589673A the tetrahydropyrazolopyrimidine (THPP) family,^23^ GSK1829820A and GSK1829731A the imidazopyridine (IMP) family,^17^, ^24^ GSK2200150A the spirocyclic (Spiros) family, GSK358607A and GSK749336A the quinoloxyacetamide (QOA) family, GSK888636A the triazolopyrimidine (TAP) family, and GW623128 the adamantyl amide (Adamantyl) family.^25^ A thioquinazoline singleton was also included given the attractive MIC values shown against H37Rv.

Biological profiling of the seven families began with testing against a panel of eight bacteria species. All of the families showed high levels of mycobacteria selectivity except for TPA (Table 1), which displayed sub-micromolar MICs against both the *H.* *influenzae* H128 and *M. catarrhalis* 1502 strains.

The intracellular MICs of the compounds were also determined with the *M. bovis* BCG strain. Although not considered critical for compound progression, intracellular activity is highly desirable for new chemical hits, as it demonstrates the compound′s ability to kill tuberculosis bacteria growing inside macrophages. This ability is essential for a compound to have activity in murine efficacy models of TB infection, where most of the bacteria are found inside macrophages. Of the seven chemical families, only three displayed measurable intracellular activities: THPP, Spiros, and Adamantyl.

Next, the compounds were tested for two key physical properties: solubility and permeability. These properties are essential for the initial absorption and distribution of drugs in biological systems, and clear warnings were immediately available for the QOA (permeability) and Adamantyl (solubility) families. Less dramatic, but still significant, physical property warnings were also observed for the THPP (permeability) and TPA (both) families.

The compounds were further tested in vitro against the most significant cytochrome P450s. The CYP enzymes are particularly relevant in tuberculosis drug discovery given the need for combination chemotherapy and the prevalence of drug–drug interactions. Although few of the families were completely clean against all the tested isoforms, the most concerning results were the sub-micromolar activities of the QUO, Spiros, and TAP families.

Finally, the mouse and human microsomal stabilities of the hits were determined. For a compound to be viable in an in vivo assay, it must have a reasonable level of microsomal stability. Although some of the compounds displayed at least moderate stability in the human microsomal fraction assay, only the THPP, Adamantyl, and, to a lesser extent, the TAP families showed workable stabilities in the mouse microsomal assay. All of the other families would therefore require further optimization before being ready for an in vivo proof-of-concept assay.

Based on these complete in vitro biology and physicochemical data, some of the chemical families were further progressed by the tuberculosis group at GSK. One family (THPP) has advanced to a full lead optimization program. Three others (Spiros, IMP, and TAP) have been extensively explored as potential lead compounds. Results from these medicinal chemistry programs will be described in separate publications.

Further mechanism of action studies around the hits described herein are underway both within GSK and with a number of external partners. The methods being applied include computational docking, genomics, transcriptomics, and proteomics. Early evidence stemming from these studies seems to indicate mycobacterial cell wall biosynthesis and bacterial respiration as the most likely targets affected. The findings from these studies will be reported in due course.

## Conclusions

In conclusion, as part of GSK′s continued commitment to open innovation in neglected disease research, we are now making the data from our phenotypic screen against tuberculosis publicly available. A total of 177 compounds have been identified based on strict cutoffs of MIC_95_<10 μm and T.I. (HepG2 IC_50_/H37Rv MIC)>50. Initial explorations around the biological modes of action of these compounds have been commenced, pointing toward inhibition of mycobacterial cell wall biosynthesis and electron transport chain inhibition as the most likely mechanisms of action. Additionally, seven chemical families with strong antitubercular activities were tentatively profiled as potential lead compounds. Samples of the 177 hit structures will be made publicly available upon request, and a competitive funding scheme has been created for those interested in pursuing unexplored hit structures further.^26^ Finally, the complete data are publicly available at DOI 10.6019/CHEMBL2095176.^27^ It is our hope that the release of these data will inspire further efforts toward the ultimate goal of eradicating tuberculosis.

## Experimental Section

### *M. bovis* BCG str. Pasteur 1173P2 HTS assay

Bacterial inocula were grown for 4–5 days in Middlebrook 7H9 medium (Difco cat. # 271310) with glucose as carbon source. The culture medium contained per liter: 4.7 g Middlebrook 7H9 powder, 5 g albumin, 1 g glucose, 0.85 g NaCl, and 0.25 g Tween 80. The solution was sterilized by filtration through a 0.2 μm filter.

The HTS assay was carried out in 1536-well sterile plates (Greiner, 782074). The screening compounds were added to the plates as a 50 nL solution in neat DMSO prior to addition of the assay components by using an Echo 555 instrument (Labcyte Inc). The assay plates were subsequently filled with 5 μL of the bacterial solution (adjusted to 10^5^ bacteria per mL) using a Multidrop Combi NL instrument (Thermo Fischer Scientific Inc.). Inoculated plates were stacked in groups of 7–8 plates, with the top plate covered with a sterile lid. Plates were carefully wrapped with aluminum foil to prevent evaporation and allowed to incubate at 37 °C at 80 % relative humidity for seven days.

After the incubation period, plates were removed from the incubator and allowed to equilibrate at room temperature. Freshly reconstituted BacTiter-Glo (5 μL, Promega) was added to each well using the Multidrop Combi. After standing at room temperature for 7–8 min, the luminescence signal was quantified with an Acquest reader (Molecular Devices) in the focused luminescence mode. Every assay plate contained two columns of negative controls (ctrl 1) with DMSO, which correspond to 100 % activity reactions (maximum luminescence), and two columns of positive controls (ctrl 2) in which 100 % inhibition was reached by adding a known inhibitor (2 μm rifampicin as standard; bacterial growth completely inhibited). These controls were used to monitor assay quality through determination of *Z′* as well as for normalizing the data on a per-plate basis. The effect of a given compound is calculated as: % Inhib.=100×[(data−ctrl 1)/(ctrl 2−ctrl 1)].

While the resazurin-based method is a reliable way to test the phenotypic activity of antitubercular compounds, it is unfortunately unsuitable for HTS campaigns given the low signal-to-noise ratio and the frequent interference of fluorescent compounds. As an alternative to a resazurin-based readout, we used a commercially available system based on ATP measurement (BacTiter-Glo, Promega). This assay measures the effect of the compounds on bacterial growth by determining the amount of ATP per well, which is related to the number of living bacteria. The reagent causes bacterial cell lysis and generates a luminescent signal proportional to the amount of ATP present and thus to the number of viable cells in culture. The assay relies on the activity of a thermostable luciferase and on the properties of a buffer formulation for extracting ATP from bacteria.

To validate the final HTS assay configuration, a screen of a small compound set (validation set) was run in triplicate. The compounds (∼9800) were assayed in 1536-well plates at a final concentration of 10 μm. The assay plates displayed an average *Z′* factor of 0.72 and a standard deviation (SD) of 0.03. The correlation between the three replicates was very good, with an interclass correlation coefficient of 0.97. The obtained cutoff (average response of the sample distribution +3×SD) was 53.5 %, and the hit rate was 1.4 %. No signal patterns were detected after analysis of the data set using a pattern-recognition program developed in house.

The primary screening campaign (Figure 2) was performed in 16 runs at an average throughput of 90 plates per day (∼125 000 compounds per run). A total of 1.94×10^6^ compounds were screened at the final assay concentration of 10 μm. The resulting median *Z′* factor for the primary campaign was 0.76 with a standard deviation of 0.08, and the plate failure rate was <1 %. The average statistical cutoff for the primary HTS was 45.2 % inhibition, which corresponds to a 3.3 % hit rate or a total of 62 000 primary hits. No significant signal patterns due to evaporation were observed during the primary screen.

To ensure the appropriate sensitivity of the assay, a separate QC plate was included in each run containing a series of dose–responses of known pharmacological standards (streptomycin, isoniazid, and rifampicin). The calculated IC_50_ values were in agreement with the specifications of the assay and displayed a stable distribution throughout the HTS campaign.

### *M. tuberculosis* H37Rv inhibition assay

The measurement of the minimum inhibitory concentration (MIC) for each tested compound was performed in 96-well flat-bottom polystyrene microtiter plates. Ten twofold drug dilutions in neat DMSO starting at 50 mm were performed. These drug solutions (5 μL) were added to 95 μL Middlebrook 7H9 medium (lines A–H, rows 1–10 of the plate layout). Isoniazid was used as a positive control; eight twofold dilutions of isoniazid starting at 160 μg mL^−1^ were prepared, and this control curve (5 μL) was added to 95 μL Middlebrook 7H9 medium (row 11, lines A–H). Neat DMSO (5 μL) was added to row 12 (growth and blank controls). The inoculum was standardized to ∼1×10^7^ CFU mL^−1^ and diluted 1:100 in Middlebrook 7H9 broth (Middlebrook ADC enrichment, a dehydrated culture medium which supports growth of mycobacterial species, available from Becton–Dickinson, cat. # 211887), to produce the final inoculum of H37Rv strain (ATCC25618). This inoculum (100 μL) was added to the entire plate except G-12 and H-12 wells (blank controls). All plates were placed in a sealed box to prevent drying out of the peripheral wells and were incubated at 37 °C without shaking for six days. A resazurin solution was prepared by dissolving one tablet of resazurin (VWR International Ltd., *Resazurin Tablets for Milk Testing*, cat. # 330884Y′) in 30 mL sterile phosphate-buffered saline (PBS). Of this solution, 25 μL were added to each well. Fluorescence was measured (Spectramax M5, Molecular Devices; *λ*_ex_ 530 nm, *λ*_em_ 590 nm) after 48 h to determine the MIC value.

### HepG2 cytotoxicity assay

Actively growing HepG2 cells were removed from a T-175 TC flask using 5 mL Eagle’s MEM (containing 10 % FBS, 1 % NEAA, 1 % penicillin/streptomycin) and dispersed in the medium by repeated pipetting. Seeding density was checked to ensure that new monolayers were not >50 % confluent at the time of harvesting. Cell suspension was added to 500 mL of the same medium at a final density of 1.2×10^5^ cells mL^−1^. This cell suspension (25 μL, typically 3000 cells per well) were dispensed into the wells of 384-well clear-bottom plates (Greiner, cat. # 781091) using a Multidrop instrument. Prior to addition of the cell suspension, the screening compounds (250 nL) were dispensed into the plates with an Echo 555 instrument. Plates were allowed to incubate at 37 °C at 80 % relative humidity for 48 h under 5 % CO_2_.

After the incubation period, the plates were allowed to equilibrate at room temperature for 30 min before proceeding to develop the luminescent signal. The signal developer, CellTiter-Glo (Promega) was equilibrated at room temperature for 30 min and added to the plates (25 μL per well) using a Multidrop. The plates were left for 10 min at room temperature for stabilization and were subsequently read using a ViewLux instrument (PerkinElmer).

### General antimicrobial activity assay

Whole-cell antimicrobial activity was determined by broth microdilution using the Clinical and Laboratory Standards Institute (CLSI) recommended procedure, Document M7-A7, “Methods for Dilution Susceptibility Tests for Bacteria that Grow Aerobically”. Some compounds were evaluated against a panel of Gram-positive and Gram-negative organisms, including *Enterococcus faecium*, *Enterococcus faecalis*, *Haemophilus influenzae*, *Moraxella catarrhalis*, *Streptococcus pneumoniae*, *Escherichia coli*, and *Streptococcus pyogenes*. The minimum inhibitory concentration (MIC) was determined as the lowest concentration of compound required to produce a >80 % decrease in observed fluorescence.

## Supplementary material

As a service to our authors and readers, this journal provides supporting information supplied by the authors. Such materials are peer reviewed and may be re-organized for online delivery, but are not copy-edited or typeset. Technical support issues arising from supporting information (other than missing files) should be addressed to the authors.
