# Supplementary material for: Fueling Open-Source Drug Discovery: 177 Small-Molecule Leads against Tuberculosis
Source: ChemMedChem. 2013 Jan 10;8(2):313–21. doi: 10.1002/cmdc.201200428 (PMC3743164; doi:10.1002/cmdc.201200428)
Supplement: Supplementary file 1 [file cmdc0008-0313-SD1.pdf]

CHEMMEDCHEM
Chemistry & Drug Discovery

Supporting Information

© Copyright Wiley-VCH Verlag GmbH & Co. KGaA, 69451 Weinheim, 2013

Fueling Open-Source Drug Discovery: 177 Small-Molecule Leads against Tuberculosis

Lluís Ballell,*[a] Robert H. Bates,[a] Rob J. Young,[b] Daniel Alvarez-Gomez,[a] Emilio Alvarez-Ruiz,[a] Vanessa Barroso,[a] Delia Blanco,[a] Benigno Crespo,[a] Jaime Escribano,[a] Rubén González,[a] Sonia Lozano,[a] Sophie Huss,[a] Angel Santos-Villarejo,[a] José Julio Martín-Plaza,[a] Alfonso Mendoza,[a] María José Rebollo-Lopez,[a] Modesto Remuiñan-Blanco,[a] José Luis Lavandera,[a] Esther Pérez-Herran,[a] Francisco Javier Gamo-Benito,[a] José Francisco García-Bustos,[a] David Barros,[a] Julia P. Castro,[a] and Nicholas Cammack[a]

cmdc_201200428_sm_miscellaneous_information.pdf

GSK in-house kinetic solubility assay: 5 ml of 10 mM DMSO stock solution diluted to 100 ml with pH 7.4 phosphate buffered saline, equilibrated for 1 h at RT, filtered through Millipore Multiscreen HTS-PCF filter plates (MSSL BPC). The eluent is quantified by suitably calibrated flow injection CLND.

GSK in-house hydrophobicity assay: 10 ml of 10 mM DMSO stock solution diluted to 750 ml with octanol saturated pH 7.4 phosphate buffer and 160 ml buffer saturated octanol in a 96 well deep well block. Block sealed and inverted for 3 sets of 50 inversions, then centrifuged at 300 g for 20 min. Both phases are then quantified using generic gradient UV-HPLC.

Intracellular MIC BCG assay: Human THP-1 monocytes have been shown as a good model to study the intracellular stages of *Mycobacterium bovis* and *Mycobacterium tuberculosis*. A luminescent strain was used to infect the monocytes. This provides a much more rapid assessment of the bacterial viability (5 days) as compared to a colony forming unit (CFU) assay which takes more than 3 weeks to form countable colonies. The objective of this assay is to identify intracellular inhibitors of the *M. bovis* BCG and *M. tuberculosis* growth as part of a global effort focused on the search of new antitubercular drugs. This particular assay uses *M. bovis* BCG as a nonpathogenic surrogate of *M. tuberculosis*. In summary, the assay determines the effect of the compounds on mycobacteria growing inside phagocytes by determining luciferase activity per well, which is related to the number of living bacteria.

Protocol Steps

1) Bacterial culture

The *M. bovis* BCG strain expressing the *P. pyralis* luciferase gene (1) was grown in Middlebrook 7H9 broth supplemented with ADC (Difco Laboratories, Detroit, Mich.) and 1% (vol/vol) glycerol at 37ºC for 7 days.

2) Single cell suspension protocol:

Subculture mycobacterial broth to have an OD 600 between 0.6-1 at time of infection 0.
Pellet the mycobacterial cultures at 3500 rpm for 10 min.
Disperse the bacterial pellet by shaking with 10 glass beads during 60 seconds and centrifuge for 5 min at 1500 rpm.
Collect the supernatant and measure OD 600 nm of the dispersed bacterial suspension using a 1:4 dilution into RPMI-0.05% Tween 80.
Calculate MOI of 10 using the following conversion: An OD of 0.1 = 10^7 CFU/mL.

3) THP1 cell preparation

THP1 cells (Human acute monocytic leukemia cell line, ATCC number TIB-202) were maintained in suspension with RPMI-1640 media (Sigma) containing 10% fetal bovine serum (Lonza), 1 mM of Pyruvate (Sigma), 2mM of L-Glutamine (Sigma), and incubated at 37 ºC with 5% CO2. Monocytes were routinely subcultured every 3 days at a cell density of 10^5 cells/mL.

Monocytes were grown to sub-confluence (5x10^5 cell/mL) and infected ON in a cell roller bottle with a MOI of 10. Excess bacteria were removed by washing five times in RPMI media (1500 rpm 5min.). 100 µL/well (50,000 cells/well) of infected cells were dispensed in 96 well white plates. 1:3 serial dilutions of antitubercular agents were added to the cell plates. As DMSO damages cells, the percentage must be reduced under 0.5% by diluting 1:400 in culture medium. Luminescence was measured after 5 days using the Steady-Glo Promega kit into a Victor 1420 system. Results were processed by using an Excel spreadsheet and Grafit software. MIC90 values were calculated from the dose-response curves by non-linear regression analysis.

<u>Artificial membrane permeability assay - HPLC – Permeability:</u>

A 8% L-a-phosphatidylcholine (EPC) in 1% cholesterol decane solution and a 1.8% EPC in cholesterol decane solution were prepared. The lipid solution was then aliquoted into 4 mL capped vials, sealed with parafilm and stored in -20°C freezer. The lipid solution was then transferred from 4 mL vial into 96-well half area plate (130 µL/well) for daily assay usage. An additional 50 mM phosphate buffer with 0.5% encapsin, pH at 7.4 was prepared.

The assay was run by the Biomek FX and Biomek software. The assay procedure is written under the Biomek software. For one batch assay, it can test two 96-well sample plates with at least one standard on each sample plate. The total assay time was about 4 hours. 3.5 µL of lipid solution were added to the filler plate, shaken for 12 seconds, and 250 µL of buffer were added to donor side and 100 µL to the receiver side. The assay plate was shaken for 45 min before adding the compounds. The test compounds (2.5 µL) were added to the donor side. The assay was run as replicates: Assay plates 1 and 2 tested the sample plate 1; assay plates 3 and 4 tested the sample plate 2. The assay plates were then incubated and shaken for 3 hours at room temperature. The assay samples were transferred to the HPLC analysis plates and 100 µL of receiver solution were aspirated and transferred to the receiver for analysis. Similarly, another 100 µL from the donor solution were transferred to the donor analysis plate. Compound concentration was measured by HPLC at different time-points and permeability was established as cm/sec.

GSKnumber BCG	MIC90 H37Rv uM	SMILES		
GSK1829676A	0,2	FC(F)(F)c1nc2ccccn2c1C(=O)NCc1ccc(Oc2ccccc2)cc1		
GSK353069A	0,2	CNc1nc(SC)nc2ccccc12		
GSK1829820A	0,2	Cc1nc2ccc(C)cn2c1C(=O)NCc1cccs1		
GR135486X	0,2	Nc1cccnc1Nc1ccc(Cl)cc1		
GSK163574A	0,3	Cl.Cc1csc2nc(nc(Nc3ccncn3)c12)-c1cccc(C)n1		

GSK749336A	0,3	COc1ccc2nc(C)cc(OCC(=O)Nc3cc(C)cc(C)c3)c2c1		
GW861072X	0,3	O=C(C=Cc1cccs1)N1CCN(Cc2ccc3OCOc3c2)CC1		
GSK1329419A	0,3	OC(=O)C(F)(F)F.Nc1nc2ccc(cc2[nH]1)-c1cncc(Oc2ccc(F)cc2)c1		
GSK358607A	0,4	COc1ccc2nc(C)cc(OCC(=O)Nc3ccccc3OC)c2c1		
GSK2111534A	0,4	Cc1nc2ccccn2c1C(=O)NCc1ccc(Cl)cc1		

SB-829405	0,5	CN1CCC[C@H]1COc1c(C)cc(\C=C\c2cccc(c2)C(O)=O)cc1Cl		
GSK153890A	0,5	Cc1cccc(n1)-c1nc(Nc2ccncn2)c2sccc2n1		
GSK888636A	0,6	COc1ccc(CCNc2cc(nc3ncnn23)-c2ccccc2)cc1		
GSK1829736A	0,9	Cc1ccc2nc(c(C(=O)NCc3cc(Cl)cc(Cl)c3)n2c1)C(F)(F)F		
GSK1829729A	0,9	Cc1ccc2nc(c(C(=O)NCc3cccs3)n2c1)C(F)(F)F		

GSK2200160A	1,0	CCc1ccc(CN2CCC3(CC2)OCCc2sccc32)s1		
GW623128X	1,0	Clc1ccc2[nH]c(cc2c1)C(=O)NC12CC3CC(CC(C3)C1)C2		
GSK2200150A	1,0	C(N1CCC2(CC1)OCCc1sccc21)c1ccc2OCCOc2c1		
GSK1759150A	1,1	OC(=O)C(O)=O.CCOc1ccc(cc1)-c1nc(CNCCc2ccc(F)cc2)cs1		
GSK1829732A	1,1	Cc1ccc2nc(c(C(=O)NCc3ccc4OCOc4c3)n2c1)C(F)(F)F		

GSK463114A	1,1	COc1ccc2nc(C)cc(OCC(=O)NCc3ccccc3)c2c1		
GSK1783710A	1,1	CCc1c(C)nc([nH]c1=O)-n1nc(C)cc1NC(=O)c1ccccc1SC		
SB-204804-A	1,1	Cl.C[C@@H](NCCc1nc(no1)-c1cn(C)c2ccccc12)C1CCCCC1		
GSK1180781A	1,2	Cc1ccc(CNC(=O)c2cnn3C(CC(Nc23)c2ccc(C)cc2)C(F)F)cc1		
GSK1220329A	1,3	CCCCS(=O)(=O)Nc1ccc2cn(C)nc2c1		

GSK1829819A	1,3	Cc1nc2ccc(C)cn2c1C(=O)NCc1ccco1		
GSK1121877A	1,4	COc1ccc(cc1)-c1cncc(c1)-c1cc(nc(n1)-c1ccccc1)N1CCN(CCO)CC1		
GW339742X	1,5	CCOC(=O)CC[C@H](NC(=O)c1ccc(cc1)S(=O)(=O)Nc1ccc2nc(N)[nH]c(=O)c2c1)C(=O)OCC		
GSK1955236A	1,5	Clc1ccc(CCNc2cc(nc3ncnn23)-c2ccccc2)cc1		
GSK810016A	1,5	CCN(CC)c1ccc(cc1)-c1nn2c(nnc2s1)-c1n[nH]c2CCCc12		

GW356807A	1,6	Cl.O=C(Nc1ccc(NC2=NCCN2)cc1)c1ccc(cc1)C(=O)Nc1ccc(NC2=NCCN2)cc1		
GSK731389A	1,6	COc1ccc(cc1)-c1nsc(SCC(=O)N2CCCC2)n1		
GW876411A	1,7	OC(=O)C(F)(F)F.Cc1csc2nc(nc(Nc3ccncn3)c12)-c1ccccn1		
GSK921190A	1,7	CSc1ccccc1C(=O)Nc1nc(cs1)-c1ccccn1		
GW859039X	1,7	CC(C)(C)c1ccc(cc1)-c1nnc(SCC(=O)OC2CCCCC2)o1		

GSK1519001A	1,8	OC(=O)C(F)(F)F.C([C@H]1CN(CCN1)c1nccc(Oc2ccc(cc2)-n2ccnc2)n1)c1ccccc1		
GSK2059310A	1,8	Cc1n[nH]c(C)c1S(=O)(=O)NCc1nc(NCCCc2ccccc2)c2ccccc2n1		
SB-435634	1,8	Nc1ccccc1Nc1ccc2OCOc2c1		
GSK1744926A	1,9	Brc1ccc(CN2CCN(CC2)C(=O)C=Cc2cccs2)s1		
GSK1750922A	1,9	CN1CCN(CC1)c1ccc(cc1NC(=O)c1ccccn1)C(F)(F)F		

GSK957094A	2,0	Cc1nc2ccccn2c1C(=O)NCc1ccccc1		
GSK1829733A	2,1	CC(C)Oc1cccc(CNC(=O)c2c(nc3ccc(C)cn23)C(F)(F)F)c1		
GSK695914A	2,2	COc1ccccc1-c1cccc(CNC(=O)c2cc3ccccc3cc2OC)c1		
GR135487X	2,2	Nc1cccnc1Nc1ccc(cc1)C(F)(F)F		
GSK735816A	2,3	Fc1ccc2nc(Nc3nc(cs3)-c3ccccn3)sc2c1		

BRL-7940SA	2,3	Br.CC(C)(C)c1ccccc1OCCCON1C(=N)N=C(N)NC1(C)C		
GSK124576A	2,3	Cc1cc(OCC(=O)NCc2ccccc2)c2cc(Br)ccc2n1		
BRL-8088SA	2,3	Br.CC1(C)N=C(N)N=C(N)N1OCCc1ccc2ccccc2c1		
BRL-10988SA	2,3	Br.CCCc1cc(ccc1OCCCON1C(=N)N=C(N)NC1(C)C)C(C)=O		
CCI7967	2,4	Cc1cc(NCCc2ccccc2)nc(NC(=N)Nc2ccc(Cl)cc2)n1		

GSK254610A	2,4	FC(F)(F)c1ccc2c(SCCCN3CCc4ccc(cc4CC3)C#N)ccnc2c1		
GSK810037A	2,4	C(c1cccs1)c1nn2c(nnc2s1)-c1n[nH]c2CCCc12		
GSK426032A	2,5	CCOc1ccc(OCc2ccc(cc2)C(=O)NNC(=O)c2ccc(o2)N(=O)=O)cc1		
GR223839X	2,7	C(Sc1ccccc1)c1nn2c(nnc2s1)-c1ccccc1		
GSK735826A	2,7	C1Oc2cc3nc(Nc4nc(cs4)-c4ccccn4)sc3cc2O1		

GSK861337A	2,7	O=C(N1CCN(Cc2ccc3OCOc3c2)CC1)c1cc2ccccc2o1		
GSK829969A	2,8	CCC(Sc1nc2ccccc2o1)C(=O)Nc1nccs1		
GSK124945A	2,8	Fc1ccccc1-c1nc(C#N)c(NCCc2ccccc2)o1		
GSK1859936A	2,8	CCN(Cc1ccc2OCOc2c1)C(=O)CSc1nc(NC)c2ccccc2n1		
GSK1742694A	2,8	CCc1ccc(Nc2nnc(SCc3cc(C)on3)s2)cc1		

GSK498315A	2,9	Cc1nc2ccccn2c1C(=O)NCc1ccc2OCOc2c1		
GSK1996236A	3,0	COc1ccc(cc1)-c1cc(=O)c2cc(OCC(=O)OC(C)C)ccc2o1		
GW360240X	3,0	COc1cc(Cc2cnc(N)nc2N)c2cnccc2c1N(C)C		
SB-650816	3,1	O=C(COc1ccccc1)N1CCN(CC1)c1cnc2ccccc2n1		
GSK1829674A	3,1	FC(F)(F)c1nc2ccccn2c1C(=O)NCc1cc(Cl)cc(Cl)c1		

GSK762874A	3,1	COc1ccc(CCNC(=O)c2c(C)nc3ccccn23)cc1		
BRL-10143SA	3,2	Br.CCc1ccccc1OCCCON1C(=N)N=C(N)NC1(C)C		
GSK353071A	3,2	CSc1nc(NCC=C)c2ccccc2n1		
GW857165X	3,3	Cc1cc(OCC(=O)Nc2ccccc2)c2cc(Br)ccc2n1		
GSK1829671A	3,3	FC(F)(F)c1nc2ccccn2c1C(=O)NCc1ccc2OCOc2c1		

GSK847913A	3,3	FC(F)(F)c1cc(SCc2ccccc2)nc(n1)-c1ccccn1		
GSK994258A	3,4	COc1ccccc1OCCCCn1c(nc2ccccc12)C1CC1		
GSK353496A	3,4	CNc1nc(SC)nc2ccsc12		
GSK1829728A	3,4	Cc1ccc(CNC(=O)c2c(nc3ccc(C)cn23)C(F)(F)F)o1		
GSK547481A	3,4	C(CN1CCOCC1)Cn1c(Sc2ccnc(n2)N2CCN(CC2)c2ccncc2)nnc1-c1cccs1		

GSK1365028A	3,5	NC(=O)c1cc(ccc1OCCOc1ccccc1)-c1ccsc1		
GSK920684A	3,5	Fc1cccc(OCC(=O)Nc2nc(cs2)-c2ccccn2)c1		
BRL-51091AM	3,5	Br.CC1(C)NC(N)=NC(=N)N1OCCCOc1ccc(Cl)cc1Cl		
GSK2200157A	3,7	Cc1cc(CN2CCC3(CC2)OCCc2sccc32)oc1C		
GSK2043267A	3,7	COc1ccc(cc1OC)-c1nc(no1)-c1cccc(NC(=O)Cc2ccc(F)cc2)c1		

SB-516933	3,7	O=C(C=Cc1ccccc1)N1CCN(Cc2ccc3OCOc3c2)CC1		
GSK385518A	3,7	CC(=O)N1CCc2cc3nc(C)cc(C)c3cc12		
GSK798463A	3,7	Cc1cccc(Cn2ccc(NC(=O)c3ccc4OCOc4c3)n2)c1		
GSK1072678A	3,7	CSc1cccc(c1)C(=O)Nc1nc(cs1)-c1ccccn1		
BRL-8903SA	3,8	Br.Cc1ccccc1OCCCON1C(=N)N=C(N)NC1(C)C		

GSK1829727A	3,9	Cc1ccc2nc(c(C(=O)NCc3ccco3)n2c1)C(F)(F)F		
GSK237561A	4,0	Cc1occc1C(=O)Nc1nc(nc2ccsc12)-c1ccccn1		
GSK262906A	4,0	CC(C)(C)C(=O)CC1(O)Oc2ccccc2N=C1C=C(O)C(C)(C)C		
GR153167X	4,1	CCOC(=O)c1cc(on1)-c1csc(Nc2c(C)cc(C)cc2C)n1		
GSK2032710A	4,2	O=C(C1CCN(CC1)c1nnc(s1)-n1cccc1)N1CCC(Cc2ccccc2)CC1		

GSK1985270A	4,2	Cc1ccccc1CN1CCC(O)(CC1)c1ccc(Cl)c(c1)C(F)(F)F		
GSK1589671A	4,4	CCc1ccc(cc1)C1CC(n2ncc(C(=O)NCc3cccs3)c2N1)C(F)(F)F		
GSK381407A	4,5	NC(=Nc1nc(cc2ccccc12)-c1ccccn1)c1cccc(Cl)c1		
SB-712970	4,5	O=C(N1CCN(Cc2ccc3OCOc3c2)CC1)c1[nH]nc2CCCCc12		
GW713556X	4,6	O=C(C=Cc1ccco1)N1CCN(Cc2ccc3OCOc3c2)CC1		

GSK1826247A	4,7	Cn1ccnc1CNCCS(=O)(=O)c1cccc(Nc2ccc(cn2)-c2cccc(F)c2)c1		
SB-811137-V	4,7	OC(=O)C(F)(F)F.CCc1ccc(cc1)S(=O)(=O)Nc1cc(CN2CCN(CCC(C)C)CC2)ccc1C		
GSK754716A	4,7	Brc1c(nc2ncccn12)-c1ccc2OCOc2c1		
GSK705278A	4,7	C(Oc1ccc(cc1)-n1cncn1)c1ccc(cc1)-n1cncn1		
GSK1941290A	4,8	CC(C)n1ncc2c(cc(nc12)-c1ccccc1)C(=O)NCC(N1CCOCC1)c1cccs1		

GSK1826825A	4,9	Fc1cccc(Cl)c1COC(=O)c1ccc(cc1)-n1ncc(Cl)c(Cl)c1=O		
GSK1589673A	4,9	CCc1ccc(cc1)C1CC(n2ncc(C(=O)NCc3ccc(OC)cc3)c2N1)C(F)(F)F		
GSK847920A	5,0	Cc1nc2ccc(C)cn2c1-c1ccn(Cc2ccccc2)n1		
GSK275628A	5,2	Brc1cncc(c1)-c1cc(NCCCn2ccnc2)nc(n1)-c1ccccc1		
GSK636544A	5,2	Cc1nn(Cc2ccccc2)c(Cl)c1C(=O)NC1CCCCCC1		

GSK1434490A	5,2	Fc1ccccc1-c1noc(n1)N1CCN(Cc2ccc3OCOc3c2)CC1		
GSK1731114A	5,3	O=C(N1CCN(Cc2ccc3OCOc3c2)CC1)c1cc2CCCc2s1		
GSK345724A	5,3	COc1cc(nc2ccc(C)c(N)c12)C(F)(F)F		
BRL-51093AM	5,3	Br.Cc1cc(C)cc(OCCCON2C(=N)N=C(N)NC2(C)C)c1		
GSK937733A	5,4	Cc1ccc(cc1)C1CC(n2ncc(C(=O)NCc3ccc4OCOc4c3)c2N1)C(F)(F)F		

GSK1402290A	5,5	C(Cc1ccccc1)Nc1cc(nc2nncn12)-c1ccccc1		
GSK1588120A	5,6	OC(=O)C(F)(F)F.C(NCc1ccc(cc1)-c1cncc2ccccc12)C1CCNC1		
GSK130506A	5,6	FC(F)(F)c1cccc(c1)C(=O)Nc1nnc(o1)-c1ccc(Cl)cc1		
GSK1925843A	5,6	CNc1nc(SCC(=O)N(C)Cc2ccc(Cl)s2)nc2ccccc12		
GSK991960A	5,8	CN(C)S(=O)(=O)c1ccc(cc1)C(=O)Nc1nnc(o1)-c1ccc(Cl)s1		

GSK445886A	5,9	Clc1ccc(Nc2nc(cs2)-c2ccccn2)nc1		
GSK1302651A	5,9	Cc1nnc(NCc2cccc(Cl)c2)c2ccccc12		
GSK937213A	6,1	O=C(CCC1CCCCC1)Nc1cccc(c1)-c1nnc(o1)-c1ccco1		
GSK270670A	6,1	COc1cc(Nc2ccccc2)ccc1NS(=O)(=O)c1c(C)noc1C		
GI247341A	6,2	OC(=O)C(F)(F)F.NC(=N)c1ccc(CCN2CCC(CC2)c2nc(COCC(F)(F)F)c(o2)-c2ccc(F)cc2)cc1		

GSK1829660A	6,2	Cc1nc2ccccn2c1C(=O)Nc1ccc(cc1)C(C)(C)C		
GSK547543A	6,4	C(CN1CCOCC1)Cn1c(Sc2ccnc(n2)N2CCN(CC2)c2ccncc2)nnc1-c1ccccn1		
GI103688B	6,5	Cl.Cn1cc(NC(=O)c2cc(NC(=O)CNC(N)=N)cn2C)cc1C(=O)NCCC(N)=N		
GSK1729177A	6,6	O=C(Nc1ccccc1N1CCOCC1)[C@H](Cc1ccccc1)NC(=O)c1cccs1		
GSK1758774A	6,6	Fc1ccccc1CNC(=O)N1CCN(Cc2ccc(Br)s2)CC1		

GSK1650514A	6,6	CSc1ccc(cc1)-c1nnc(NC(=O)c2ccc(Cl)s2)o1		
GSK1812410A	6,7	OC1(Oc2ccccc2N=C1c1ccccc1)c1ccccc1		
GSK831784A	6,8	CC(C)OC(=O)COc1ccc(cc1)-c1cnc(nc1)N(C)CCc1ccncc1		
GSK1857145A	6,8	COc1ccc(CC(=O)Nc2c(nc3cc(C)ccn23)-c2cccs2)cc1		
GSK848336A	7,1	C1CC1c1cc(NC2CCCCC2)nc(n1)-c1ccccn1		

GSK1051703A	7,1	COc1ccc(Nc2ccccc2NC(=O)c2ccc(O)nc2)cc1		
GSK437009A	7,4	C1CCCN(CC1)c1nc(nc(n1)-n1ccnc1)-c1ccccc1		
GSK276001A	7,4	CN1CCN(CC1)c1cc(nc(n1)-c1ccncc1)-c1ccc(F)cc1		
GSK1826089A	7,6	COc1ccc(cn1)-c1ccc(Nc2cccc(c2)S(=O)(=O)CCNCCc2cccs2)nc1		
GSK479031A	7,6	Cc1ccc2nc(Cl)c(CNCc3cccs3)cc2c1		

GSK1055950A	7,6	O=C(Nc1ccccc1N1CCOCC1)C(Cc1ccccc1)NC(=O)c1cccs1		
GSK1829816A	7,7	CC(C)CNC(=O)c1c(C)nc2ccc(C)cn12		
GSK1905227A	7,7	Cc1ccc2[nH]c(C3CCCNC3)c(-c3ccncc3)c2c1		
SB-811796-V	7,8	OC(=O)C(F)(F)F.CCCc1ccc(cc1)S(=O)(=O)Nc1ccc(CN2CCN(CC3CC3)CC2)cc1		
GSK1863309A	7,8	Cc1onc(c1C(=O)NCCSc1ccc(C)cc1)-c1ccccc1		

GSK316438A	7,8	Clc1ccc(s1)C(=O)Nc1nnc(o1)-c1ccc(Cl)s1		
SB-706404	7,9	CC1=Nc2ccccc2CN1CC(O)c1cc(Br)cs1		
GSK547511A	7,9	C(Cn1c(Cc2ccccc2)nnc1Sc1ccnc(n1)N1CCN(CC1)c1ccncc1)N1CCOCC1		
GSK1691553A	7,9	O=C(Nc1nc(cs1)-c1ccccn1)c1cncn1-c1ccccc1		
GSK146660A	7,9	CCCOC(=O)COc1ccc2c(c1)occ(-c1ccccc1OC)c2=O		

GSK468214A	8,0	CCc1nnc(NC(=O)c2cccc(n2)C(=O)Nc2nnc(CC)s2)s1		
GSK1733953A	8,1	COc1ccc(cc1)-c1cc(NC(=O)c2ccc(F)cc2Cl)ccc1OC		
GSK690382A	8,2	COc1ccc2sc(Nc3nc(cs3)-c3ccccn3)nc2c1		
SB-552112	8,2	O=C(\C=C\c1ccccc1)N1CCN(Cc2cccc(OCc3ccccc3)c2)CC1		
GSK2157753A	8,3	O=C(CCCSc1ccccc1)NNC(=O)c1cccs1		

GSK1832831A	8,3	CC(C)c1ccc(cc1)-c1cncc(n1)N1CCN(CC1)c1ccncc1		
GSK347301A	8,3	O=C(NNC(=O)c1ccc2ccccc2c1)c1ccc(o1)N(=O)=O		
GSK1385423A	8,4	CCn1c2ccc(cc2[nH]c(=O)c1=O)C(=O)Nc1ccccc1Nc1ccc(OC)cc1		
GSK1372568A	8,4	Cc1ccc(s1)C(=O)Nc1nc(cs1)-c1ccccn1		
GSK1788487A	8,4	Cl.Clc1cccc2sc(nc12)N(CCCn1ccnc1)C(=O)c1ccco1		

GSK920703A	8,4	Clc1ccc(SCCC(=O)Nc2nc(cs2)-c2ccccn2)cc1		
GSK1107112A	8,6	Fc1ccc2c(Cl)c(sc2c1)C(=O)NC1=NCCS1		
GSK1598164A	8,6	C(Nc1cccnc1)c1ccc(s1)-c1cc2ccncc2cc1OC1CCNCC1		
GSK921295A	8,7	CSc1ccc(cc1)C(=O)Nc1nc(cs1)-c1ccccn1		
GW664700A	8,7	OC(=O)C(F)(F)F.O=C(Nc1ccccc1-c1nc(cs1)C1CCC1)OCC1CCNCC1		

GW369335X	8,7	Nc1nc(N)c2c(Sc3ccc4ccccc4c3)cccc2n1		
GSK889423A	8,8	CCc1nc2ccccc2n1CC(O)COc1cc(C)ccc1Cl		
GSK1668869A	8,9	Clc1ccc(cc1)S(=O)(=O)c1oc(nc1C#N)-c1ccccc1		
GSK547487A	8,9	CC(=O)NCCn1c(Sc2ccnc(n2)N2CCN(CC2)c2ccncc2)nnc1-c1ccccc1		
GSK892651A	9,0	Cc1nc2ccccn2c1C(=O)NCc1cccs1		

GSK275984A	9,0	CN1CCN(CC1)c1cc(nc(n1)-c1ccccc1)-c1ccncc1		
GSK352635A	9,1	FC(F)(F)c1nnc2ccc(Cl)nn12		
SB-746177	9,2	CCCCc1ccc(nc1)C(=O)Nc1nccc2ccccc12		
GSK1518999A	9,2	OC(=O)C(F)(F)F.Cc1cnc(nc1Oc1ccc(cc1)-n1ccnc1)N1CCN[C@@H](Cc2ccccc2)C1		
GSK1570606A	9,3	Fc1ccc(CC(=O)Nc2nc(cs2)-c2ccccn2)cc1		

GV187303X	9,4	Nc1cc(OCc2ccccc2)ccc1Nc1ccccc1		
SB-354364	9,5	COc1cc(CN2CCC3(CC2)C=Cc2ccccc32)cc2OCOc12		
GSK1635139A	9,5	COc1ccc2cc(sc2c1)C(=O)Nc1ccc(cc1)C1CCN(C)CC1		
GSK1310678A	9,5	Oc1ccc(F)cc1C(=O)c1cnn(c1)C(=O)c1ccco1		
GSK1611550A	9,8	CC(Oc1ccccc1)C(=O)Nc1nc(cs1)-c1ccccn1		

GSK1174628A	9,8	Cn1c2nsc(S(C)=O)c2c(=O)n(C)c1=O		
GSK133167A	9,8	OC(=O)C(F)(F)F.COc1cccc(n1)-c1nc2c(cccc2n1CC1CCCN1)N1CCCC1		

GSKnumber BCG	parent mw	aring	cmr	flex	hba	hbd	liphba	liphbd	mw	naring	rb	sp2	sp3	tpsa	ChromLogD_v3.value	ChromLogD_v3.SFI	clogp_day.value	pKa_acd_v11.a1	pKa_acd_v11.b1	pKa_acd_v11.acidclass	pKa_acd_v11.baseclass
GSK1829676A	411,38	4	10,613	12	3	1	5	1	411,38	0	4	23	7	55,63	5,53	9,53	4,988	10,88	4,69	1	1
GSK353069A	205,28	2	6,057	13	2	1	3	1	205,28	0	2	10	4	37,81	4,23	6,23	3,085		5,66	0	1
GSK1829820A	285,36	3	8,175	13	2	1	4	1	285,36	0	3	16	4	46,4	4,07	7,07	3,471	11,59	5,39	1	1
GR135486X	219,67	2	6,217	6	1	2	3	2	219,67	0	1	12	3	50,94	4,15	6,15	3,587		5,53	0	1
GSK163574A	334,4	4	9,449	11	5	1	6	1	370,86	0	3	21	4	76,48	5,19	9,19	3,286		3,01	0	1
GSK749336A	350,41	3	10,17	17	2	1	5	1	350,41	0	5	18	8	60,45	5,73	8,73	4,832	12,65	6,66	1	2
GW861072X	356,44	2	10,034	17	1	0	5	0	356,44	2	5	15	10	42,01	5,52	7,52	3,658		6,12	0	2
GSK1329419A	320,32	4	8,944	6	4	2	7	3	434,34	0	2	23	8	114,12	3,28	7,28	3,87	10,31	6,88	1	2
GSK358607A	352,38	3	9,859	21	2	1	6	1	352,38	0	6	18	8	69,68	4,57	7,57	3,319	12,02	6,66	1	2
GSK2111534A	299,76	3	8,393	13	2	1	4	1	299,76	0	3	17	4	46,4	4,67	7,67	3,809	11,8	5,39	1	1
SB-829405	385,88	2	10,989	17	2	0	4	1	385,88	1	5	16	11	49,77	3,93	5,93	4,112	4,18	9,59	7	7
GSK153890A	320,37	4	8,985	11	5	1	6	1	320,37	0	3	21	2	76,48	4,8	8,8	2,997		2,97	0	1
GSK888636A	345,4	4	10,108	17	3	1	6	1	345,4	0	5	21	5	64,34	5,7	9,7	4,202		1,92	0	1
GSK1829736A	402,2	3	9,395	10	2	1	4	1	402,2	0	3	17	9	46,4	5,14	8,14	4,815	10,4	4,57	1	1
GSK1829729A	339,34	3	8,221	12	2	1	4	1	339,34	0	3	16	7	46,4	3,85	6,85	3,035	10,48	4,58	1	1
GSK2200160A	333,51	2	9,623	12	1	0	2	0	333,51	2	3	10	12	12,47	5,15	7,15	3,741		8,04	0	5
GW623128X	328,84	2	9,271	7	1	2	3	2	328,84	3	2	11	12	44,89	6,24	8,24	4,923			0	0
GSK2200150A	357,47	2	9,943	6	1	0	4	0	357,47	3	2	11	14	30,93	4,38	6,38	2,991		8,38	0	5
GSK1759150A	356,46	3	10,165	21	5	1	7	3	446,49	0	7	21	10	108,75	4,56	7,56	4,741		7,54	0	4
GSK1829732A	377,32	3	9,005	10	2	1	6	1	377,32	1	3	17	10	64,86	3,9	6,9	3,354	10,89	4,61	1	1
GSK463114A	336,38	3	9,706	22	2	1	5	1	336,38	0	6	18	7	60,45	4,98	7,98	3,86	14	6,71	1	2
GSK1783710A	383,47	3	10,714	17	4	2	7	2	383,47	0	5	20	7	92,67	3,35	6,35	1,715	7,23		3	0
SB-204804-A	352,47	3	10,402	20	2	1	5	1	388,93	1	6	14	13	55,88	4,45	7,45	4,267		9,35	0	7
GSK1180781A	410,46	3	11,267	15	2	2	5	2	410,46	1	5	19	11	58,95	5,73	8,73	4,069		1,33	0	1
GSK1220329A	267,35	2	7,155	21	3	0	5	1	267,35	0	4	11	6	63,99	4,27	6,27	2,269	9,41	0,67	1	1
GSK1829819A	269,3	3	7,58	13	2	1	5	1	269,3	0	3	16	4	59,54	3,55	6,55	2,771	11,7	5,4	1	1
GSK1121877A	467,56	4	13,702	12	4	1	7	1	467,56	1	5	24	11	74,61	5,21	9,21	4,513		6,42	0	2
GW339742X	545,56	3	13,709	22	7	3	13	4	545,56	0	9	25	12	199,64	2,38	5,38	1,045	7,85	2,95	2	1
GSK1955236A	349,82	4	9,983	14	3	1	5	1	349,82	0	4	21	4	55,11	6,25	10,25	4,996		1,9	0	1
GSK810016A	379,48	4	10,661	12	4	1	7	1	379,48	1	4	19	8	75	5,33	9,33	4,199	11,67	4,27	1	1
GW356807A	482,54	3	13,733	7	2	6	10	6	519	2	3	26	11	131,04	0,03	3,03	1,361	12,14	10,4	1	7
GSK731389A	335,44	2	9,02	20	3	0	5	0	335,44	1	5	13	9	55,32	5,06	7,06	2,357			0	0
GW876411A	320,37	4	8,985	9	7	1	8	2	434,4	0	3	23	7	113,78	3,63	7,63	2,787		3,01	0	1
GSK921190A	327,42	3	9,236	16	3	1	4	1	327,42	0	4	19	3	54,88	4,92	7,92	2,727	6,98	0,72	4	1
GW859039X	374,5	2	10,375	17	3	0	5	0	374,5	1	5	13	13	65,22	7,01	9,01	4,937			0	0
GSK1519001A	412,49	4	12,05	12	6	1	9	2	526,51	1	5	25	13	105,4	3,11	7,11	3,995		8,56	0	6
GSK2059310A	450,56	4	12,588	22	5	3	8	3	450,56	0	8	23	8	112,66	4,57	8,57	4,741	4,88		7	0
SB-435634	228,25	2	6,53	10	0	2	4	2	228,25	1	2	12	5	56,51	3,47	5,47	2,487		5,02	0	1
GSK1744926A	397,35	2	10,028	20	1	0	3	0	397,35	1	5	14	8	23,55	6,08	8,08	4,244		5,56	0	1
GSK1750922A	364,36	2	9,246	10	2	1	5	1	364,36	1	3	14	12	48,47	3,92	5,92	3,394	11,12	7,44	1	3
GSK957094A	265,31	3	7,902	13	2	1	4	1	265,31	0	3	17	3	46,4	4,08	7,08	3,096	12,66	5,45	1	1
GSK1829733A	391,39	3	9,957	16	2	1	5	1	391,39	0	5	17	11	55,63	4,79	7,79	4,146	10,9	4,61	1	1

GSK695914A	397,47	4	11,965	18	1	1	4	1	397,47	0	6	24	6	47,56	6,61	10,61	5,381	13,29		1	0
GR135487X	253,22	2	6,236	10	1	2	3	2	253,22	0	2	12	6	50,94	4,3	6,3	3,836		5,18	0	1
GSK735816A	328,39	4	8,768	12	3	1	4	1	328,39	0	3	20	2	50,7	5,86	9,86	4,277		1,28	0	1
BRL-7940SA	347,46	1	9,872	23	1	3	7	3	428,37	1	6	10	16	95,96	5,17	6,17	3,827		5,97	0	1
GSK124576A	385,25	3	9,866	19	2	1	4	1	385,25	0	5	18	6	51,22	5,62	8,62	4,476	13,95	5,86	1	1
BRL-8088SA	311,38	2	9,24	16	1	2	6	2	392,29	1	4	14	10	89,23	3,3	5,3	1,767		8,32	0	5
BRL-10988SA	375,47	1	10,371	32	2	3	8	3	456,38	1	9	12	16	113,03	4,54	5,54	3,298		5,96	0	1
CCI7967	380,87	3	10,933	17	3	4	6	4	380,87	0	5	20	7	85,72	4,48	7,48	3,676		5,56	0	1
GSK254610A	441,51	3	11,909	14	2	0	3	0	441,51	1	5	16	13	39,92	5,86	8,86	5,812		8,38	0	5
GSK810037A	328,42	4	8,71	11	4	1	6	1	328,42	1	3	18	4	71,76	4,2	8,2	2,566	11,74		1	0
GSK426032A	425,39	3	10,97	21	3	2	10	2	425,39	0	7	23	7	135,62	4,74	7,74	3,876	9,09		1	0
GR223839X	324,42	4	9,274	8	3	0	4	0	324,42	0	2	20	2	43,08	5,74	9,74	3,442			0	0
GSK735826A	354,41	4	9,345	10	3	1	6	1	354,41	1	3	20	4	69,16	5,6	9,6	4,659		2,17	0	1
GSK861337A	364,39	3	10,073	12	1	0	6	0	364,39	2	4	17	10	55,15	5,13	8,13	3,58		6,01	0	2
GSK829969A	319,4	3	8,554	21	3	1	5	1	319,4	0	5	16	5	68,02	4,85	7,85	3,166	7,79	2,26	2	1
GSK124945A	307,32	3	8,503	20	2	1	4	1	307,32	0	5	17	4	61,85	5,88	8,88	4,576			0	0
GSK1859936A	410,49	3	11,42	25	3	1	7	1	410,49	1	8	18	11	76,58	5,23	8,23	4,269		4,52	0	1
GSK1742694A	332,44	3	9,131	25	3	1	5	1	332,44	0	6	16	6	63,84	6,04	9,04	4,266		0,59	0	1
GSK498315A	309,32	3	8,495	11	2	1	6	1	309,32	1	3	17	6	64,86	4,02	7,02	3,061	11,98	5,4	1	1
GSK1996236A	368,38	3	9,952	17	2	0	6	0	368,38	0	5	19	8	74,97	5,99	8,99	3,977			0	0
GW360240X	324,38	3	9,369	11	3	2	7	2	324,38	0	3	16	8	103,18	2,83	5,83	2,239		7,12	0	3
SB-650816	348,4	3	9,997	13	3	0	6	0	348,4	1	4	18	8	58,56	4,63	7,63	3,245		5,57	0	1
GSK1829674A	388,17	3	8,931	11	2	1	4	1	388,17	0	3	17	8	46,4	5,12	8,12	4,316	10,39	4,65	1	1
GSK762874A	309,36	3	8,983	20	2	1	5	1	309,36	0	5	17	6	55,63	4,27	7,27	3,144	12,44	5,44	1	1
BRL-10143SA	319,4	1	8,944	29	1	3	7	3	400,31	1	7	10	14	95,96	4,45	5,45	3,029		5,97	0	1
GSK353071A	231,32	2	6,959	23	2	1	3	1	231,32	0	4	12	4	37,81	4,84	6,84	3,859		5,62	0	1
GW857165X	371,23	3	9,403	12	2	1	4	1	371,23	0	3	18	5	51,22	5,52	8,52	4,45	12,6	5,89	1	1
GSK1829671A	363,29	3	8,541	10	2	1	6	1	363,29	1	3	17	9	64,86	3,82	6,82	2,855	10,88	4,69	1	1
GSK847913A	347,36	3	8,858	15	3	0	3	0	347,36	0	4	18	6	38,67	6,23	9,23	4,308			0	0
GSK994258A	336,43	3	9,985	28	1	0	4	0	336,43	1	8	15	10	36,28	6,33	9,33	4,458		6,36	0	2
GSK353496A	211,31	2	5,866	14	2	1	3	1	211,31	0	2	9	4	37,81	4,28	6,28	3,133		5,09	0	1
GSK1829728A	337,3	3	8,09	11	2	1	5	1	337,3	0	3	16	8	59,54	3,74	6,74	3,064	10,6	4,59	1	1
GSK547481A	549,71	4	15,218	20	5	0	10	0	549,71	2	9	22	16	88,33	1,16	5,16	3,074		10,73	0	7
GSK1365028A	339,41	3	9,622	23	1	1	4	1	339,41	0	6	19	5	61,55	5,2	8,2	4,086			0	0
GSK920684A	329,35	3	8,598	20	3	1	5	1	329,35	0	5	19	4	64,11	4,94	7,94	3,115	7,44	0,74	3	1
BRL-51091AM	360,24	1	8,999	25	1	3	7	3	441,15	1	6	10	14	95,96	4,76	5,76	3,386		5,95	0	1
GSK2200157A	317,45	2	9,028	8	1	0	3	0	317,45	2	2	10	12	25,61	4,52	6,52	3,191		8,2	0	5
GSK2043267A	433,43	4	11,595	20	3	1	7	1	433,43	0	7	25	7	86,48	5,57	9,57	4,362	13,76		1	0
SB-516933	350,41	2	10,377	13	1	0	5	0	350,41	2	4	16	10	42,01	5,61	7,61	4,012		6,25	0	2
GSK385518A	240,3	2	7,175	5	2	0	3	0	240,3	1	1	12	6	33,2	4,76	6,76	2,247		6,2	0	2
GSK798463A	335,36	3	9,318	14	2	1	6	1	335,36	1	4	19	6	65,38	4,56	7,56	3,67	12,3	0,94	1	1
GSK1072678A	327,42	3	9,236	16	3	1	4	1	327,42	0	4	19	3	54,88	5,37	8,37	3,427	6,75	0,75	4	1
BRL-8903SA	305,38	1	8,48	26	1	3	7	3	386,29	1	6	10	13	95,96	4,03	5,03	2,5		5,97	0	1
GSK1829727A	323,27	3	7,626	12	2	1	5	1	323,27	0	3	16	7	59,54	3,43	6,43	2,565	10,6	4,59	1	1
GSK237561A	336,37	4	9,121	11	4	1	6	1	336,37	0	3	22	2	80,91	4,54	8,54	2,984	8,26	1,16	2	1

GSK262906A	345,43	1	9,741	11	4	2	5	2	345,43	1	3	12	13	79,12	5,31	6,31	3,814	9,08	2,23	1	1
GR153167X	357,43	3	9,652	18	3	1	6	1	357,43	0	5	18	7	77,25	6,28	9,28	5,635		0,23	0	1
GSK2032710A	435,58	3	12,511	17	3	0	6	0	435,58	2	6	18	13	54,26	6,06	9,06	3,043		1,88	0	1
GSK1985270A	383,84	2	9,793	10	1	1	2	1	383,84	1	3	12	14	23,47	5,73	7,73	4,785	13,46	7,48	1	3
GSK1589671A	434,48	3	11,091	15	2	2	5	2	434,48	1	5	18	12	58,95	5,64	8,64	3,373	13,82	1,03	1	1
GSK381407A	358,82	4	10,492	10	2	1	4	1	358,82	0	3	24	2	64,16	5,13	9,13	3,409		4,06	0	1
SB-712970	368,43	2	10,067	12	2	1	7	1	368,43	3	4	13	14	70,69	3,96	5,96	2,868		6,14	0	2
GW713556X	340,37	2	9,439	17	1	0	6	0	340,37	2	5	15	10	55,15	5,09	7,09	3,188		6,14	0	2
GSK1826247A	465,54	4	12,71	25	4	2	7	2	465,54	0	9	25	7	88,91	4,38	8,38	3,427		7,51	0	4
SB-811137-V	443,64	2	13,03	20	4	0	7	2	557,67	1	8	16	21	89,95	3,96	5,96	5,722	8,23	9,06	2	7
GSK754716A	318,13	3	7,265	4	2	0	5	0	318,13	1	1	15	4	48,65	4,67	7,67	3,004		2,08	0	1
GSK705278A	318,33	4	8,854	14	4	0	7	0	318,33	0	4	22	2	70,65	3,94	7,94	2,092		2,58	0	1
GSK1941290A	475,61	4	13,602	15	4	1	7	1	475,61	1	6	22	12	72,28	6,08	10,08	4,399	12,28	6,28	1	2
GSK1826825A	427,64	3	10,108	10	3	0	5	0	427,64	0	3	21	6	61,19	6,41	9,41	4,705			0	0
GSK1589673A	458,48	3	11,899	16	2	2	6	2	458,48	1	6	19	14	68,18	5,81	8,81	3,646		1,08	0	1
GSK847920A	302,37	4	9,227	11	2	0	4	0	302,37	0	3	20	3	35,12	5,56	9,56	4,332		5,84	0	1
GSK275628A	435,32	4	11,344	19	4	1	6	1	435,32	0	6	23	5	68,52	5,64	9,64	4,373		6,97	0	2
GSK636544A	345,87	2	9,775	15	2	1	4	1	345,87	1	4	13	11	46,92	5,47	7,47	3,788	13,53		1	0
GSK1434490A	382,39	3	9,99	12	2	0	7	0	382,39	2	4	17	11	63,86	5,41	8,41	4,177		6,16	0	2
GSK1731114A	370,46	2	10,194	13	1	0	5	0	370,46	3	4	13	13	42,01	5,37	7,37	3,809		5,95	0	1
GSK345724A	256,22	2	6,125	5	1	1	3	1	256,22	0	1	10	8	48,14	4,28	6,28	3,367		2,36	0	1
BRL-51093AM	319,4	1	8,944	25	1	3	7	3	400,31	1	6	10	14	95,96	4,52	5,52	2,999		5,97	0	1
GSK937733A	458,43	3	11,411	10	2	2	7	2	458,43	2	4	19	14	77,41	5,35	8,35	3,163	13,88	1,06	1	1
GSK1402290A	315,37	4	9,491	14	3	1	5	1	315,37	0	4	21	3	55,11	5,86	9,86	4,466		3,19	0	1
GSK1588120A	317,43	3	10,019	15	3	2	5	3	431,45	1	5	18	13	74,25	0,68	3,68	3,266		10,69	0	7
GSK130506A	367,71	3	8,373	7	3	1	5	1	367,71	0	2	19	6	68,02	5,82	8,82	4,092	10,17		1	0
GSK1925843A	392,93	3	10,665	25	3	1	5	1	392,93	0	7	17	8	58,12	5,22	8,22	4,176		4,52	0	1
GSK991960A	412,87	3	9,84	10	5	1	8	1	412,87	0	3	20	5	105,4	4,47	7,47	2,285	9,43		1	0
GSK445886A	288,76	3	7,747	14	3	1	4	1	288,76	0	3	17	2	50,7	5,14	8,14	3,464		1,47	0	1
GSK1302651A	283,76	3	8,253	13	2	1	3	1	283,76	0	3	16	4	37,81	5,06	8,06	4,209		6,18	0	2
GSK937213A	365,43	3	10,118	20	3	1	6	1	365,43	1	6	18	9	81,16	6,13	9,13	4,622			0	0
GSK270670A	373,43	3	9,868	14	3	1	7	2	373,43	0	4	19	6	93,46	4,96	7,96	3,82	7,98		2	0
GI247341A	504,52	3	12,782	17	4	2	8	3	618,54	1	8	21	22	125,67	1,95	4,95	3,247		11,89	0	7
GSK1829660A	307,39	3	9,293	8	2	1	4	1	307,39	0	2	17	6	46,4	5,63	8,63	4,743	13,06	5,34	1	1
GSK547543A	544,67	4	15,198	20	6	0	11	0	544,67	2	9	23	16	101,22	0,44	4,44	1,986		10,73	0	7
GI103688B	430,46	2	11,399	28	3	8	13	8	466,92	0	9	20	12	208,93	-2,67	-0,67	-2,278		11,73	0	7
GSK1729177A	435,54	3	12,384	20	3	2	6	2	435,54	1	7	21	10	70,67	4,27	7,27	2,882	13,05	5,41	1	1
GSK1758774A	412,32	2	10,012	19	1	1	4	1	412,32	1	5	13	11	35,58	5,52	7,52	4,352	13,33	5,44	1	1
GSK1650514A	351,83	3	8,941	12	3	1	5	1	351,83	0	3	18	4	68,02	5,45	8,45	3,497	10,01		1	0
GSK1812410A	301,34	3	8,959	3	2	1	3	1	301,34	1	1	20	3	41,82	5,46	8,46	4,372	9,13	2,31	1	1
GSK831784A	406,48	3	11,499	25	4	0	7	0	406,48	0	8	20	10	77,44	5,11	8,11	3,583		5,83	0	1
GSK1857145A	377,46	4	10,839	16	2	1	5	1	377,46	0	5	22	5	55,63	4,98	8,98	4,18	11,02	6	1	1
GSK848336A	294,39	2	8,795	16	3	1	4	1	294,39	2	4	12	10	50,7	5,84	7,84	4,419		4,13	0	1
GSK1051703A	335,36	3	9,507	14	3	3	6	3	335,36	0	4	20	5	83,48	3,75	6,75	3,816	0,25	8,56	7	6
GSK437009A	320,39	3	9,27	7	4	0	6	0	320,39	1	2	17	7	59,73	5,17	8,17	3,048		4,69	0	1

GSK276001A	349,4	3	9,972	3	3	0	5	0	349,4	1	1	18	8	45,15	4,7	7,7	3,415		7,15	0	3
GSK1826089A	494,63	4	13,691	29	4	2	7	2	494,63	0	11	25	8	93,21	4,97	8,97	4,715		7,78	0	4
GSK479031A	302,82	3	8,737	18	1	1	2	1	302,82	0	4	15	5	24,92	5,16	8,16	3,497		6,15	0	2
GSK1055950A	435,54	3	12,384	20	3	2	6	2	435,54	1	7	21	10	70,67	4,27	7,27	2,882	13,05	5,41	1	1
GSK1829816A	245,32	2	7,246	15	2	1	4	1	245,32	0	3	11	7	46,4	3,86	5,86	3,084	12,83	5,48	1	1
GSK1905227A	291,39	3	9,08	4	1	2	3	2	291,39	1	1	15	7	40,71	2,51	5,51	3,217		10,13	0	7
SB-811796-V	427,6	2	12,429	20	4	0	7	2	541,63	2	8	16	20	89,95	4,03	6,03	5,299	8,13	7,81	2	4
GSK1863309A	352,45	3	10,243	14	2	1	4	1	352,45	0	4	19	6	55,13	6,08	9,08	4,183	13,48		1	0
GSK316438A	346,21	3	7,972	13	3	1	5	1	346,21	0	3	17	3	68,02	5,27	8,27	3,487	9,87		1	0
SB-706404	351,26	2	8,641	13	1	1	3	1	351,26	1	3	13	7	35,83	4,51	6,51	3,986	13,59	8,63	1	6
GSK547511A	543,69	4	15,409	20	5	0	10	0	543,69	2	9	23	16	88,33	2,51	6,51	2,454		10,73	0	7
GSK1691553A	347,39	4	9,696	10	4	1	6	1	347,39	0	3	24	1	72,7	4,88	8,88	3,274	6,55	1,82	4	1
GSK146660A	368,38	3	9,952	24	2	0	6	0	368,38	0	7	19	8	74,97	5,69	8,69	3,137			0	0
GSK468214A	389,46	3	9,865	21	7	2	9	2	389,46	0	6	20	6	122,65	2,02	5,02	1,282	5,24		7	0
GSK1733953A	385,82	3	10,32	13	1	1	4	1	385,82	0	4	20	7	47,56	6	9	4,145	11,26		1	0
GSK690382A	340,42	4	9,37	15	3	1	5	1	340,42	0	4	20	3	59,93	5,77	9,77	4,438		2,52	0	1
SB-552112	412,52	3	12,913	20	1	0	4	0	412,52	1	7	22	9	32,78	6,65	9,65	5,734		6,17	0	2
GSK2157753A	320,43	2	8,943	27	2	2	4	2	320,43	0	6	15	6	58,2	4,57	6,57	3,151	10,69		1	0
GSK1832831A	359,47	3	10,884	13	2	0	5	0	359,47	1	4	18	9	45,15	4,63	7,63	4,454		10,73	0	7
GSK347301A	325,28	3	8,45	11	3	2	8	2	325,28	0	3	21	2	117,16	3,59	6,59	2,745	9,12		1	0
GSK1385423A	430,46	4	12,184	14	3	3	8	3	430,46	0	5	26	6	105,22	4,27	8,27	3,402	9,79	1,58	1	1
GSK1372568A	301,39	3	8,239	13	3	1	4	1	301,39	0	3	18	2	54,88	4,53	7,53	3,003	6	0,8	5	1
GSK1788487A	386,86	4	10,18	24	3	0	6	0	423,32	0	7	21	6	64,16	4,67	8,67	2,529		6,97	0	2
GSK920703A	375,9	3	10,191	19	3	1	4	1	375,9	0	5	19	5	54,88	6,33	9,33	4,491	8,7	0,77	1	1
GSK1107112A	314,79	2	7,772	4	2	1	3	1	314,79	1	1	13	6	41,46	4,72	6,72	2,462	8,61	3,91	1	1
GSK1598164A	416,54	4	12,282	17	2	2	5	2	416,54	1	6	21	9	59,07	2,45	6,45	3,34		9,61	0	7
GSK921295A	327,42	3	9,236	12	3	1	4	1	327,42	0	3	19	3	54,88	5,4	8,4	3,427	6,98	0,75	4	1
GW664700A	371,5	2	10,471	14	4	2	7	3	485,52	2	5	15	18	100,55	1,89	3,89	3,932	13,16	10,26	1	7
GW369335X	318,4	4	9,697	7	2	2	4	2	318,4	0	2	20	3	77,82	3,8	7,8	4,742		8,56	0	6
GSK889423A	344,84	3	9,687	23	2	1	4	1	344,84	0	6	15	9	47,28	5,8	8,8	4,403	13,67	6,16	1	2
GSK1668869A	344,77	3	8,555	4	4	0	5	0	344,77	0	1	19	1	83,96	4,94	7,94	3,306			0	0
GSK547487A	501,61	4	14,077	17	5	1	10	1	501,61	1	7	25	11	104,96	0,55	4,55	1,944		10,73	0	7
GSK892651A	271,34	3	7,711	14	2	1	4	1	271,34	0	3	16	3	46,4	4,02	7,02	2,972	11,57	5,37	1	1
GSK275984A	331,41	3	9,957	3	3	0	5	0	331,41	1	1	18	7	45,15	4,64	7,64	3,269		6,81	0	2
GSK352635A	222,56	2	4,174	0	3	0	4	0	222,56	0	0	9	5	43,08	3,64	5,64	1,087			0	0
SB-746177	305,37	3	9,189	20	3	1	4	1	305,37	0	5	18	5	54,88	5,55	8,55	4,417	10,45	3,99	1	1
GSK1518999A	426,51	4	12,514	11	6	1	9	2	540,54	1	5	25	14	105,4	3,46	7,46	4,494		8,56	0	6
GSK1570606A	313,35	3	8,445	16	3	1	4	1	313,35	0	4	19	3	54,88	4,99	7,99	2,996	8,68	0,77	1	1
GV187303X	290,36	3	9,065	16	0	2	3	2	290,36	0	4	18	4	47,28	5,22	8,22	4,315		4,39	0	1
SB-354364	349,42	2	10,108	10	0	0	4	0	349,42	3	3	14	12	30,93	4,96	6,96	4,614		8,51	0	6
GSK1635139A	380,5	3	11,156	13	1	1	4	1	380,5	1	4	17	10	41,57	3,92	6,92	4,415	12,32	9,5	1	7
GSK1310678A	300,24	3	7,311	16	4	1	6	1	300,24	0	4	20	2	85,33	4,66	7,66	3,859	7,21		3	0
GSK1611550A	325,38	3	9,047	16	3	1	5	1	325,38	0	4	19	4	64,11	5,16	8,16	3,141	7,34	0,74	3	1
GSK1174628A	259,3	2	6,209	5	4	0	6	0	259,3	0	1	13	3	73,96	2,05	4,05	-0,545			0	0
GSK133167A	377,48	3	11,069	13	4	1	8	2	491,51	2	5	17	18	92,51	1,97	4,97	4,073		11,11	0	7

CMPD_NUMBER	CLND_CONC_MOD	CLND_CONC_UM_MEAN	CHROM_LOGD_PH74_MEAN
BRL-10143SA	>=	225	
BRL-10988SA	>=	201	
BRL-51091AM		337	
BRL-51093AM		358	
BRL-7940SA		363	
BRL-8088SA		201	
BRL-8903SA	>=	436	
CCI7967			3,93
GI103688B		108	
GI247341A		255	
GR135487X		328	
GR153167X	<	1	
GR223839X	<	1	
GSK1051703A	>=	362	
GSK1055950A		64	
GSK1072678A		12	
GSK1107112A	<	1	
GSK1121877A	<	1	
GSK1174628A	>=	420	
GSK1180781A	<	1	
GSK1220329A	>=	552	
GSK124576A		15	
GSK124945A		3	
GSK1302651A		127	
GSK130506A	<	1	
GSK1310678A	<	1	
GSK1329419A		100	
GSK133167A		78	
GSK1365028A	<	1	
GSK1372568A		15	
GSK1385423A		47	
GSK1402290A		6	

GSK1434490A		10	
GSK1518999A		228	
GSK1519001A		260	
GSK1570606A		14	
GSK1588120A		61	
GSK1589671A		1	7,14
GSK1598164A			
GSK1611550A		174	
GSK1635139A		62	
GSK1650514A		67	
GSK1668869A	<	1	
GSK1691553A		118	
GSK1729177A		52	
GSK1731114A		117	
GSK1733953A	<	1	
GSK1742694A	<	1	
GSK1744926A		152	
GSK1750922A		182	
GSK1758774A		60	
GSK1759150A		159	
GSK1783710A	<	1	
GSK1788487A		21	
GSK1812410A		263	
GSK1826089A	<	1	
GSK1826247A		64	
GSK1826825A		22	
GSK1829660A		85	
GSK1829671A		115	
GSK1829674A	<	1	
GSK1829676A	<	1	
GSK1829727A		159	
GSK1829728A	>=	259	
GSK1829729A		17	

GSK1829732A		22	
GSK1829733A	<	1	
GSK1829736A	<	1	
GSK1829816A	>=	287	
GSK1829819A	>=	277	
GSK1832831A		89	
GSK1857145A		87	
GSK1859936A		74	
GSK1863309A		50	
GSK1905227A	>=	267	
GSK1925843A		56	
GSK1941290A	<	1	
GSK1955236A	<	1	
GSK1985270A		77	
GSK2032710A	<	1	
GSK2043267A		102	
GSK2059310A		240	
GSK2111534A	>=	405	
GSK2157753A	>=	559	
GSK2200157A	>=	289	
GSK237561A		105	
GSK254610A	<	1	
GSK262906A		75	
GSK270670A		53	
GSK275628A		15	
GSK275984A	>=	379	
GSK276001A		44	
GSK345724A	>=	446	
GSK347301A	<	1	
GSK352635A	>=	344	
GSK353071A		279	
GSK353496A	>=	439	
GSK381407A	<	1	

GSK385518A	>=	309	
GSK426032A	<	1	
GSK437009A	<	1	
GSK445886A		14	
GSK463114A		96	
GSK468214A		26	
GSK479031A		44	
GSK498315A	>=	357	
GSK547481A	>=	185	
GSK547487A		135	
GSK547511A		186	
GSK547543A		154	
GSK636544A		28	
GSK690382A		22	
GSK695914A		84	
GSK731389A		10	
GSK735816A		28,5	
GSK735826A	<	1	
GSK754716A		219	
GSK762874A	>=	348	
GSK798463A		80	
GSK810016A	<	1	
GSK810037A		196	
GSK829969A		105	
GSK831784A		182	
GSK847913A	<	1	
GSK847920A		120	
GSK848336A		302	
GSK861337A		171	
GSK889423A	<	1	
GSK892651A	>=	365	
GSK920684A		14	
GSK921295A		2	

GSK937213A	<	1	
GSK957094A	>=	403	
GSK991960A		177	
GSK994258A		167	
GV187303X	<	1	
GW339742X		300	
GW356807A		26	
GW360240X	>=	304	
GW369335X		32	
GW664700A		115	
GW713556X		186	
GW857165X		16	
GW859039X	<	1	
GW861072X	>=	314	
GW876411A		231	
SB-204804-A		202	
SB-354364		121	
SB-435634		139	
SB-552112		61	
SB-650816	>=	173	
SB-706404	>=	563	
SB-746177		98	
SB-811137-V		313	
SB-811796-V		318	
SB-829405		486	

CMPD_NUMBER	AVG_CHROM_LOGD_PH7_4	Solubility CLND MEAN_AQ_CONC_UM
CCI7967	3,93	
GSK153890A	3,5	
GSK1589671A	7,14	
GSK1589673A	6,83	
GSK163574A	5,56	
GSK1829820A	3,63	
GSK2200150A	4,54	
GSK2200160A	8,18	
GSK316438A	3,48	
GSK353069A	3,85	
GSK358607A	4,87	
GSK749336A	5,23	
GSK888636A	4,78	
GSK920703A	5,86	
GSK921190A	4,59	
GSK937733A	6,49	
GW623128X	7,61	
GSK153890A		277
GSK1589673A		0
GSK163574A		23
GSK1829820A		339
GSK2200150A		260
GSK2200160A		53
GSK316438A		4
GSK353069A		443
GSK358607A		38
GSK468214A		35
GSK735816A		16
GSK749336A		32
GSK888636A		14
GSK920703A		5
GSK921190A		34

GSK937733A		13
GSK991960A		169
GW623128X		1
GW876411A		231

CMPD_NUMBER	CMPD_NUMBER	CHI_LOGD_PH74_MEAN	CLND_CONC_UM_MEAN
	CCI7967	2,17	
	GSK1072678A		
GSK153890A	GSK153890A	1,9	277
	GSK1589671A	4,13	
GSK1589673A	GSK1589673A	3,94	0
	GSK1598164A		
GSK163574A	GSK163574A	3,17	23
GSK1829820A	GSK1829820A	1,98	339
GSK2200150A	GSK2200150A	2,54	260
GSK2200160A	GSK2200160A	4,77	53
	GSK237561A		
GSK316438A	GSK316438A	1,89	4
GSK353069A	GSK353069A	2,12	443
GSK358607A	GSK358607A	2,74	38
	GSK468214A		35
	GSK735816A		16
GSK749336A	GSK749336A	2,96	32
GSK888636A	GSK888636A	2,69	14
GSK920703A	GSK920703A	3,35	5
GSK921190A	GSK921190A	2,57	34
GSK937733A	GSK937733A	3,74	13
	GSK991960A		169
GW623128X	GW623128X	4,42	1
	GW876411A	awol for meas	231
	GSK705278A	awol	

CMPD_NUMBER	mCHROM LOGD PH7.4	mCHROM LOGP	CLND Sol uM	SFI
BRL-10143SA	3,3	4,83	225	4,3
BRL-10988SA	2,92	4,02	201	3,92
BRL-51091AM	3,39	4,83	337	4,39
BRL-51093AM	3,2	4,72	358	4,2
BRL-7940SA	4,04	6,14	363	5,04
BRL-8088SA	2,92	4,23	201	4,92
BRL-8903SA	2,83	3,92	436	3,83
CCI7967	4	6,74	73	7
GI103688B			108	
GI247341A	3,76	6,14	255	6,76
GR135486X			94	
GR135487X	4,97	5,03	328	6,97
GR153167X	7,12	7,45	1	10,12
GR223839X	5,44	5,78	1	9,44
GSK1051703A	2,92	2,97	362	5,92
GSK1055950A	5,53	5,78	64	8,53
GSK1072678A	5,25	5,26	12	8,25
GSK1107112A	5,16	5,49	1	7,16
GSK1121877A	5,91	6,34	1	9,91
GSK1174628A	1,24	1,24	420	3,24
GSK1180781A	6,84	7,09	1	9,84
GSK1220329A	3,48	3,58	552	5,48
GSK124576A	4,97	5,13	15	7,97
GSK124945A	6,37	6,63	3	9,37
GSK1302651A	4,32	4,52	127	7,32
GSK130506A	4,51	6,29	1	7,51
GSK1310678A			1	
GSK1329419A	3,11	3,12	100	7,11
GSK133167A			78	
GSK1365028A	5,44	5,78	1	8,44
GSK1372568A	4,6	4,61	15	7,6
GSK1385423A	3,67	3,87	47	7,67

GSK1402290A	4,97	5,13	6	8,97
GSK1434490A	6,56	6,94	10	9,56
GSK146660A	6	6,24		9
GSK1518999A	4,04	5,03	228	8,04
GSK1519001A	3,58	4,33	260	7,58
GSK1570606A	4,32	4,33	14	7,32
GSK1588120A	1,52	3,02	61	4,52
GSK1589671A	6,99	7,27	1	9,99
GSK1598164A	1,9	4,23	167	5,9
GSK1611550A	5,07	5,06	174	8,07
GSK1635139A	3,11	5,93	62	6,11
GSK1650514A	3,76	6,07	67	6,76
GSK1668869A	7,12	7,48	1	10,12
GSK1691553A	3,39	3,38	118	7,39
GSK1729177A	5,53	5,78	52	8,53
GSK1731114A	6,09	6,34	117	8,09
GSK1733953A	6,28	6,48	1	9,28
GSK1742694A	5,72	5,98	1	8,72
GSK1744926A	6,47	6,74	152	8,47
GSK1750922A	6,19	6,84	182	8,19
GSK1758774A	5,72	5,93	60	7,72
GSK1759150A	6,09	7,45	159	9,09
GSK1783710A	5,63	6,48	1	8,63
GSK1788487A	5,25	5,63	21	9,25
GSK1812410A	6,09	6,19	263	9,09
GSK1826089A	5,63	5,83	1	9,63
GSK1826247A	4,41	4,62	64	8,41
GSK1826825A			22	
GSK1829660A	5,81	6,03	85	8,81
GSK1829671A	4,23	4,38	115	7,23
GSK1829674A	6,09	6,48	1	9,09
GSK1829676A	6,28	6,58	1	10,28
GSK1829727A	4,32	4,48	159	7,32

GSK1829728A	4,97	5,18	259	7,97
GSK1829729A	4,79	4,98	17	7,79
GSK1829732A	4,79	4,98	22	7,79
GSK1829733A	6,19	6,43	1	9,19
GSK1829736A	6,65	7,04	1	9,65
GSK1829816A	3,39	3,41	287	5,39
GSK1829819A	3,02	3,02	277	6,02
GSK1832831A	4,79	6,74	89	7,79
GSK1857145A	3,95	4,02	87	7,95
GSK1859936A	4,69	4,83	74	7,69
GSK1863309A	6,37	6,68	50	9,37
GSK1905227A	2,18	3,72	267	
GSK1925843A	5,07	5,23	56	8,07
GSK1941290A	7,21	7,54	1	11,21
GSK1955236A	5,53	5,73	1	9,53
GSK1985270A			77	
GSK1996236A	5,91	6,29		8,91
GSK2032710A	6,37	6,78	1	9,37
GSK2043267A	6,37	6,68	102	10,37
GSK2059310A	4,32	4,52	240	8,32
GSK2111534A	4,04	4,12	405	7,04
GSK2157753A	3,67	3,77	559	5,67
GSK2200157A			289	
GSK237561A	4,79	4,93	105	8,79
GSK254610A	7,96	8,64	1	10,96
GSK262906A	7,49	7,84	75	8,49
GSK270670A	5,53	5,78	53	8,53
GSK275628A	5,53	5,83	15	9,53
GSK275984A	4,88	5,54	379	7,88
GSK276001A	5,07	5,73	44	8,07
GSK345724A	4,97	5,13	446	6,97
GSK347301A	3,02	3,48	1	6,02
GSK352635A	3,11	3,1	344	5,11

GSK353071A	4,97	5,13	279	6,97
GSK353496A	3,3	3,3	439	5,3
GSK381407A	8,24	8,64	1	12,24
GSK385518A	2,64	2,65	309	4,64
GSK426032A	4,32	4,88	1	7,32
GSK437009A			1	
GSK445886A	5,35	6,53	14	8,35
GSK463114A	3,86	3,92	96	6,86
GSK468214A			26	
GSK479031A	6,84	7,23	44	9,84
GSK498315A	3,02	3,02	357	6,02
GSK547481A	2,08	3,12	185	6,08
GSK547487A	1,06	1,82	135	5,06
GSK547511A	2,18	3,12	186	6,18
GSK547543A	2,08	3,22	154	6,08
GSK636544A	6,75	7,04	28	8,75
GSK690382A	5,35	5,34	22	9,35
GSK695914A	7,21	7,58	84	11,21
GSK731389A	4,97	5,27	10	6,97
GSK735816A	5,53	5,54	28,5	9,53
GSK735826A	5,07	5,06	1	9,07
GSK754716A	3,48	3,48	219	6,48
GSK762874A	3,39	3,41	348	6,39
GSK798463A	5,07	5,27	80	8,07
GSK810016A	5,44	5,78	1	9,44
GSK810037A	3,3	3,48	196	7,3
GSK829969A	5,44	5,78	105	8,44
GSK831784A	5,63	5,93	182	8,63
GSK847913A	7,12	7,54	1	10,12
GSK847920A	5,16	5,54	120	9,16
GSK848336A	6,09	6,74	302	8,09
GSK861337A	5,63	5,83	171	8,63
GSK889423A	5,35	5,63	1	8,35

GSK892651A	2,92	2,93	365	5,92
GSK920684A	4,69	4,69	14	7,69
GSK921295A	5,07	5,06	2	8,07
GSK937213A	6,84	7,18	1	9,84
GSK957094A	3,2	3,2	403	6,2
GSK991960A	2,83	4,69	177	5,83
GSK994258A	5,63	5,83	167	8,63
GV187303X	6,65	6,94	1	9,65
GW339742X	2,46	2,45	300	5,46
GW356807A	0,12	0,918	26	3,12
GW360240X	2,55	2,61	304	5,55
GW369335X	4,41	4,72	32	8,41
GW664700A	4,41	8,15	115	6,41
GW713556X	4,51	4,72	186	6,51
GW857165X	5,25	5,44	16	8,25
GW859039X	9,17	9,65	1	11,17
GW861072X	4,97	5,23	314	6,97
GW876411A			231	
SB-204804-A	6,56	8,75	202	9,56
SB-354364	7,03	8,25	121	9,03
SB-435634			139	
SB-516933	5,16	5,44		7,16
SB-552112	7,21	7,54	61	10,21
SB-650816	4,69	4,72	173	7,69
SB-706404	2,46	4,12	563	4,46
SB-746177	6,65	6,94	98	9,65
SB-811137-V	6,28	7,74	313	8,28
SB-811796-V	4,69	5,54	318	6,69
SB-829405			486	

GSKnumber	Structure	Database	SMILES	BCG_MIC90_(µM)	list	list2
GSK1452496A	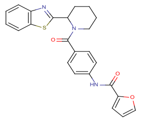	gskversion	O=C(Nc1ccc(cc1)C(=O)N1CCCCC1c1nc2ccccc2s1)c1ccco1	0,10	BCGset	
GSK1452498A	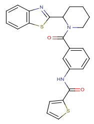	gskversion	O=C(Nc1cccc(c1)C(=O)N1CCCCC1c1nc2ccccc2s1)c1cccs1	0,10	BCGset	
SB-790594-A	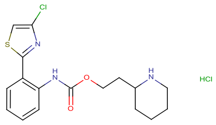	gskversion	Clc1csc(n1)-c1ccccc1NC(=O)OCCC1CCCCN1	0,20	BCGset	
GSK1829676A	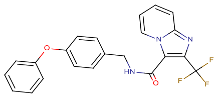	gskversion	FC(F)(F)c1nc2ccccn2c1C(=O)NCc1ccc(Oc2ccccc2)cc1	0,20	BCGset	TBset
GSK1829820A	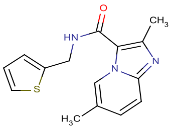	gskversion	Cc1nc2ccc(C)cn2c1C(=O)NCc1cccs1	0,20	BCGset	TBset
GSK353069A	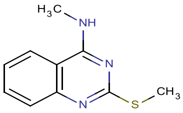	gskversion	CNc1nc(SC)nc2ccccc12	0,20	BCGset	TBset
GR135486X	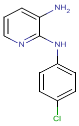	gskversion	Nc1cccnc1Nc1ccc(Cl)cc1	0,23	BCGset	TBset
GSK1917391A	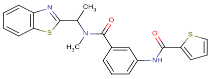	gskversion	CC(N(C)C(=O)c1cccc(NC(=O)c2cccs2)c1)c1nc2ccccc2s1	0,30	BCGset	
GSK2057544A	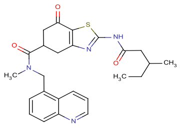	gskversion	CCC(C)CC(=O)Nc1nc2CC(CC(=O)c2s1)C(=O)N(C)Cc1cccc2ncccc12	0,30	BCGset	
GSK749336A	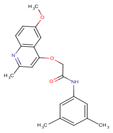	gskversion	COc1ccc2nc(C)cc(OCC(=O)Nc3cc(C)cc(C)c3)c2c1	0,30	BCGset	TBset
GW861072X	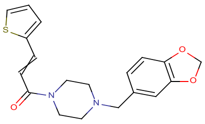	gskversion	O=C(C=Cc1cccs1)N1CCN(Cc2ccc3OCOc3c2)CC1	0,30	BCGset	TBset
GSK163574A	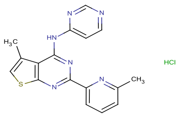	gskversion	Cc1csc2nc(nc(Nc3ccncn3)c12)-c1cccc(C)n1	0,30	BCGset	TBset
GSK1329419A	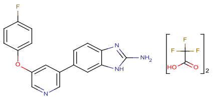	gskversion	Nc1nc2ccc(cc2[nH]1)-c1cncc(Oc2ccc(F)cc2)c1	0,35	BCGset	TBset

BRL-35822GM	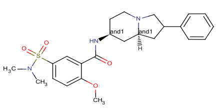	gskversion	COc1ccc(cc1C(=O)N[C@H]1CCN2CC(C[C@H]2C1)c1ccccc1)S(=O)(=O)N(C)C	0,40	BCGset	
GSK1188380A	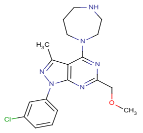	gskversion	COCc1nc(N2CCCNCC2)c2c(C)nn(-c3cccc(Cl)c3)c2n1	0,40	BCGset	
GSK1462954A	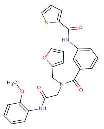	gskversion	COc1ccccc1NC(=O)CN(Cc1ccco1)C(=O)c1cccc(NC(=O)c2cccs2)c1	0,40	BCGset	
GW335118X	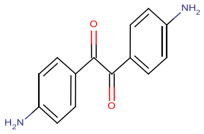	gskversion	Nc1ccc(cc1)C(=O)C(=O)c1ccc(N)cc1	0,40	BCGset	
GW874798X	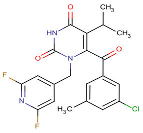	gskversion	CC(C)c1c(C(=O)c2cc(C)cc(Cl)c2)n(Cc2cc(F)nc(F)c2)c(=O)[nH]c1=O	0,40	BCGset	
GSK2111534A	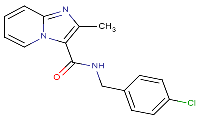	gskversion	Cc1nc2ccccn2c1C(=O)NCc1ccc(Cl)cc1	0,40	BCGset	TBset
GSK358607A	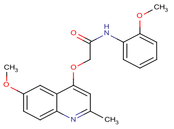	gskversion	COc1ccc2nc(C)cc(OCC(=O)Nc3ccccc3OC)c2c1	0,40	BCGset	TBset
SB-829405	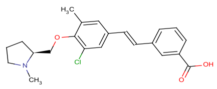	gskversion	CN1CCC[C@H]1COc1c(C)cc(\C=C\c2cccc(c2)C(O)=O)cc1Cl	0,46	BCGset	TBset
GSK186929A	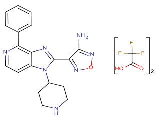	gskversion	Nc1nonc1-c1nc2c(nccc2n1C1CCNCC1)-c1ccccc1	0,50	BCGset	
GSK1956504A	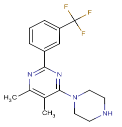	gskversion	Cc1nc(nc(N2CCNCC2)c1C)-c1cccc(c1)C(F)(F)F	0,50	BCGset	
GSK425180A	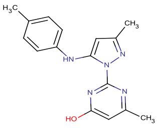	gskversion	Cc1cc(Nc2ccc(C)cc2)n(n1)-c1nc(C)cc(O)n1	0,50	BCGset	
GW315922A	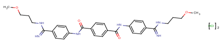	gskversion	COCCCNC(=N)c1ccc(NC(=O)c2ccc(cc2)C(=O)Nc2ccc(cc2)C(=N)NCCCOC)cc1	0,50	BCGset	
SKF-18326	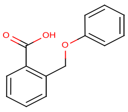	gskversion	OC(=O)c1ccccc1COc1ccccc1	0,50	BCGset	

GSK153890A	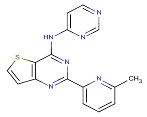	gskversion	Cc1cccc(n1)-c1nc(Nc2ccncn2)c2sccc2n1	0,50	BCGset	TBset
GSK888636A	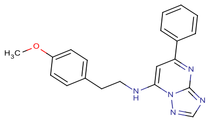	gskversion	COc1ccc(CCNc2cc(nc3ncnn23)-c2ccccc2)cc1	0,60	BCGset	TBset
GSK1691926A	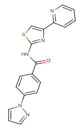	gskversion	O=C(Nc1nc(cs1)-c1ccccn1)c1ccc(cc1)-n1cccn1	0,70	BCGset	
GSK1188387A	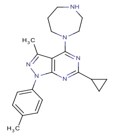	gskversion	Cc1nn(-c2ccc(C)cc2)c2nc(nc(N3CCCNCC3)c12)C1CC1	0,80	BCGset	
GSK1277133A	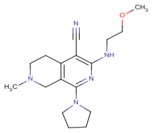	gskversion	COCCNc1nc(N2CCCC2)c2CN(C)CCc2c1C#N	0,80	BCGset	
GSK1279755A	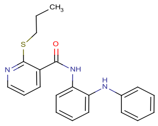	gskversion	CCCSc1ncccc1C(=O)Nc1ccccc1Nc1ccccc1	0,80	BCGset	
GSK1783663A	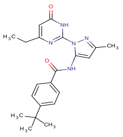	gskversion	CCc1cc(=O)[nH]c(n1)-n1nc(C)cc1NC(=O)c1ccc(cc1)C(C)(C)C	0,80	BCGset	
GSK370378A	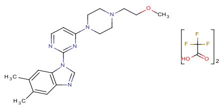	gskversion	COCCN1CCN(CC1)c1ccnc(n1)-n1cnc2cc(C)c(C)cc12	0,80	BCGset	
GW315921B	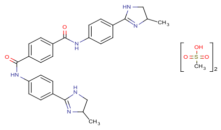	gskversion	CC1CNC(=N1)c1ccc(NC(=O)c2ccc(cc2)C(=O)Nc2ccc(cc2)C2=NC(C)CN2)cc1	0,80	BCGset	
GSK1281628A	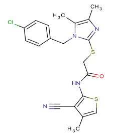	gskversion	Cc1csc(NC(=O)CSc2nc(C)c(C)n2Cc2ccc(Cl)cc2)c1C#N	0,90	BCGset	
GSK984529A	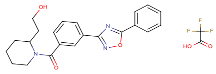	gskversion	OCCC1CCCCN1C(=O)c1cccc(c1)-c1noc(n1)-c1ccccc1	0,90	BCGset	
GW354586X	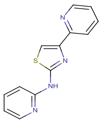	gskversion	N(c1nc(cs1)-c1ccccn1)c1ccccn1	0,90	BCGset	
GSK1829736A	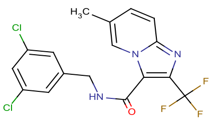	gskversion	Cc1ccc2nc(c(C(=O)NCc3cc(Cl)cc(Cl)c3)n2c1)C(F)(F)F	0,90	BCGset	TBset

GSK1829729A	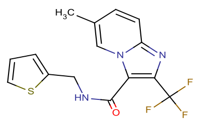	gskversion	Cc1ccc2nc(c(C(=O)NCc3cccs3)n2c1)C(F)(F)F	0,90	BCGset	TBset
GSK362766A	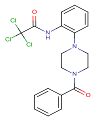	gskversion	ClC(Cl)(Cl)C(=O)Nc1ccccc1N1CCN(CC1)C(=O)c1ccccc1	1,00	BCGset	
GSK2200160A	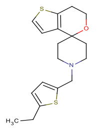	gskversion	CCc1ccc(CN2CCC3(CC2)OCCc2sccc32)s1	1,00	BCGset	TBset
GSK2200150A	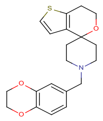	gskversion	C(N1CCC2(CC1)OCCc1sccc21)c1ccc2OCCOc2c1	1,00	BCGset	TBset
GW623128X	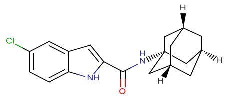	gskversion	Clc1ccc2[nH]c(cc2c1)C(=O)NC12CC3CC(CC(C3)C1)C2	1,00	BCGset	TBset
GSK1759150A	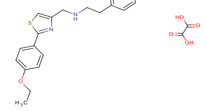	gskversion	CCOc1ccc(cc1)-c1nc(CNCCc2ccc(F)cc2)cs1	1,07	BCGset	TBset
BRL-47858HG	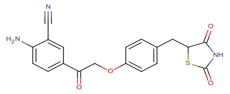	gskversion	Nc1ccc(cc1C#N)C(=O)COc1ccc(CC2SC(=O)NC2=O)cc1	1,10	BCGset	
GSK1188060A	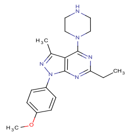	gskversion	CCc1nc(N2CCNCC2)c2c(C)nn(-c3ccc(OC)cc3)c2n1	1,10	BCGset	
GSK1213629A	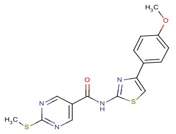	gskversion	COc1ccc(cc1)-c1csc(NC(=O)c2cnc(SC)nc2)n1	1,10	BCGset	
GSK747165A	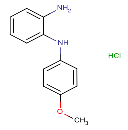	gskversion	COc1ccc(Nc2ccccc2N)cc1	1,10	BCGset	
GW432086X	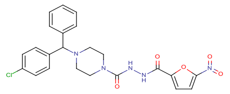	gskversion	Clc1ccc(cc1)C(N1CCN(CC1)C(=O)NNC(=O)c1ccc(o1)N(=O)=O)c1ccccc1	1,10	BCGset	
GSK1829732A	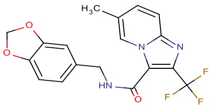	gskversion	Cc1ccc2nc(c(C(=O)NCc3ccc4OCOc4c3)n2c1)C(F)(F)F	1,10	BCGset	TBset
GSK463114A	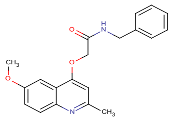	gskversion	COc1ccc2nc(C)cc(OCC(=O)NCc3ccccc3)c2c1	1,10	BCGset	TBset

GSK1783710A	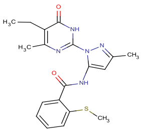	gskversion	CCc1c(C)nc([nH]c1=O)-n1nc(C)cc1NC(=O)c1ccccc1SC	1,15	BCGset	TBset
SB-204804-A	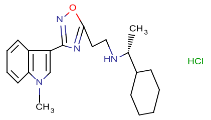	gskversion	C[C@@H](NCCc1nc(no1)-c1cn(C)c2ccccc12)C1CCCCC1	1,15	BCGset	TBset
GSK1567463A	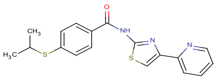	gskversion	CC(C)Sc1ccc(cc1)C(=O)Nc1nc(cs1)-c1ccccn1	1,20	BCGset	
GSK1783695A	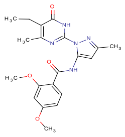	gskversion	CCc1c(C)nc([nH]c1=O)-n1nc(C)cc1NC(=O)c1ccc(OC)cc1OC	1,20	BCGset	
GSK1783704A	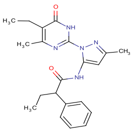	gskversion	CCC(C(=O)Nc1cc(C)nn1-c1nc(C)c(CC)c(=O)[nH]1)c1ccccc1	1,20	BCGset	
GSK468296A	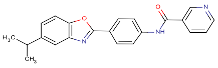	gskversion	CC(C)c1ccc2oc(nc2c1)-c1ccc(NC(=O)c2cccnc2)cc1	1,20	BCGset	
GSK905372A	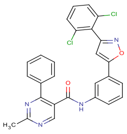	gskversion	Cc1ncc(C(=O)Nc2cccc(c2)-c2cc(no2)-c2c(Cl)cccc2Cl)c(n1)-c1ccccc1	1,20	BCGset	
GSK1180781A	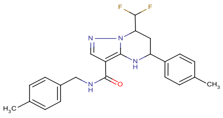	gskversion	Cc1ccc(CNC(=O)c2cnn3C(CC(Nc23)c2ccc(C)cc2)C(F)F)cc1	1,23	BCGset	TBset
GSK1783652A	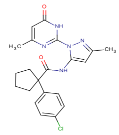	gskversion	Cc1cc(NC(=O)C2(CCCC2)c2ccc(Cl)cc2)n(n1)-c1nc(C)cc(=O)[nH]1	1,30	BCGset	
GSK1825997A	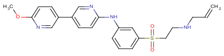	gskversion	COc1ccc(cn1)-c1ccc(Nc2cccc(c2)S(=O)(=O)CCNCC=C)nc1	1,30	BCGset	
GSK1829819A	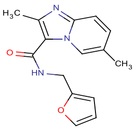	gskversion	Cc1nc2ccc(C)cn2c1C(=O)NCc1ccco1	1,30	BCGset	TBset
GSK1220329A	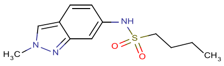	gskversion	CCCCS(=O)(=O)Nc1ccc2cn(C)nc2c1	1,30	BCGset	TBset
GI210527X	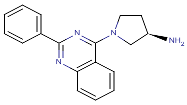	gskversion	N[C@@H]1CCN(C1)c1nc(nc2ccccc12)-c1ccccc1	1,40	BCGset	

GSK1467632A	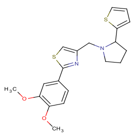	gskversion	COc1ccc(cc1OC)-c1nc(CN2CCCC2c2cccs2)cs1	1,40	BCGset	
GSK1595876A	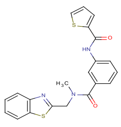	gskversion	CN(Cc1nc2ccccc2s1)C(=O)c1cccc(NC(=O)c2cccs2)c1	1,40	BCGset	
GSK1783682A	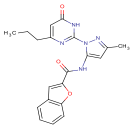	gskversion	CCCc1cc(=O)[nH]c(n1)-n1nc(C)cc1NC(=O)c1cc2ccccc2o1	1,40	BCGset	
GSK434954A	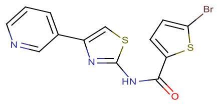	gskversion	Brc1ccc(s1)C(=O)Nc1nc(cs1)-c1cccnc1	1,40	BCGset	
GW652926X	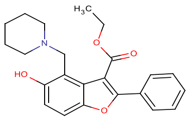	gskversion	CCOC(=O)c1c(oc2ccc(O)c(CN3CCCCC3)c12)-c1ccccc1	1,40	BCGset	
GSK1121877A	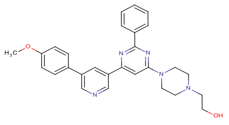	gskversion	COc1ccc(cc1)-c1cncc(c1)-c1cc(nc(n1)-c1ccccc1)N1CCN(CCO)CC1	1,45	BCGset	TBset
GSK1072525A	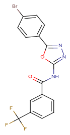	gskversion	FC(F)(F)c1cccc(c1)C(=O)Nc1nnc(o1)-c1ccc(Br)cc1	1,50	BCGset	
GSK1256528A	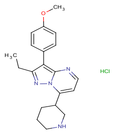	gskversion	CCc1nn2c(ccnc2c1-c1ccc(OC)cc1)C1CCCNC1	1,50	BCGset	
GSK796865A	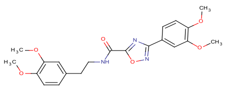	gskversion	COc1ccc(CCNC(=O)c2nc(no2)-c2ccc(OC)c(OC)c2)cc1OC	1,50	BCGset	
SB-708224	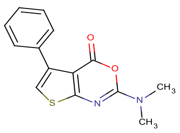	gskversion	CN(C)c1nc2scc(-c3ccccc3)c2c(=O)o1	1,50	BCGset	
GSK1955236A	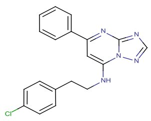	gskversion	Clc1ccc(CCNc2cc(nc3ncnn23)-c2ccccc2)cc1	1,50	BCGset	TBset
GSK810016A	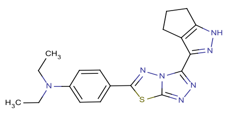	gskversion	CCN(CC)c1ccc(cc1)-c1nn2c(nnc2s1)-c1n[nH]c2CCCc12	1,50	BCGset	TBset
GW339742X	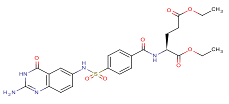	gskversion	CCOC(=O)CC[C@H](NC(=O)c1ccc(cc1)S(=O)(=O)Nc1ccc2nc(N)[nH]c(=O)c2c1)C(=O)O	1,50	BCGset	TBset

GSK1303102A	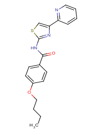	gskversion	CCCCOc1ccc(cc1)C(=O)Nc1nc(cs1)-c1ccccn1	1,60	BCGset	
GSK1472560A	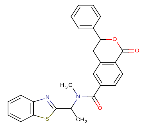	gskversion	CC(N(C)C(=O)c1ccc2C(=O)OC(Cc2c1)c1ccccc1)c1nc2ccccc2s1	1,60	BCGset	
GSK1525120A	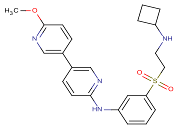	gskversion	COc1ccc(cn1)-c1ccc(Nc2cccc(c2)S(=O)(=O)CCNC2CCC2)nc1	1,60	BCGset	
GSK975839A	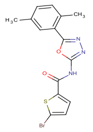	gskversion	Cc1ccc(C)c(c1)-c1nnc(NC(=O)c2ccc(Br)s2)o1	1,60	BCGset	
GW283925A	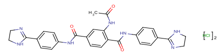	gskversion	CC(=O)Nc1cc(ccc1C(=O)Nc1ccc(cc1)C1=NCCN1)C(=O)Nc1ccc(cc1)C1=NCCN1	1,60	BCGset	
GW652646X	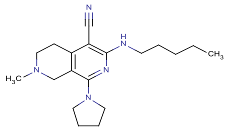	gskversion	CCCCCNc1nc(N2CCCC2)c2CN(C)CCc2c1C#N	1,60	BCGset	
SB-557875	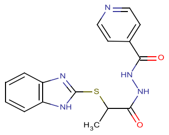	gskversion	CC(Sc1nc2ccccc2[nH]1)C(=O)NNC(=O)c1ccncc1	1,60	BCGset	
GW356807A	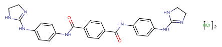	gskversion	O=C(Nc1ccc(NC2=NCCN2)cc1)c1ccc(cc1)C(=O)Nc1ccc(NC2=NCCN2)cc1	1,60	BCGset	TBset
GSK731389A	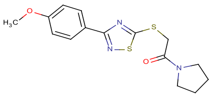	gskversion	COc1ccc(cc1)-c1nsc(SCC(=O)N2CCCC2)n1	1,60	BCGset	TBset
GSK1063699A	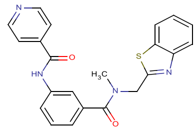	gskversion	CN(Cc1nc2ccccc2s1)C(=O)c1cccc(NC(=O)c2ccncc2)c1	1,70	BCGset	
GSK492450A	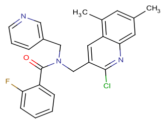	gskversion	Cc1cc(C)c2cc(CN(Cc3cccnc3)C(=O)c3ccccc3F)c(Cl)nc2c1	1,70	BCGset	
GW876411A	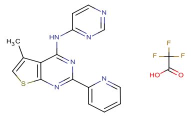	gskversion	Cc1csc2nc(nc(Nc3ccncn3)c12)-c1ccccn1	1,70	BCGset	TBset
GSK921190A	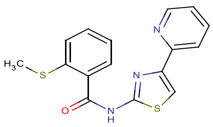	gskversion	CSc1ccccc1C(=O)Nc1nc(cs1)-c1ccccn1	1,74	BCGset	TBset

GW859039X	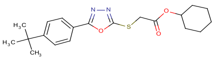	gskversion	CC(C)(C)c1ccc(cc1)-c1nnc(SCC(=O)OC2CCCCC2)o1	1,74	BCGset	TBset
GSK1519001A	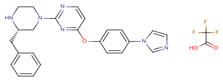	gskversion	C([C@H]1CN(CCN1)c1nccc(Oc2ccc(cc2)-n2ccnc2)n1)c1ccccc1	1,80	BCGset	TBset
GSK1286645A	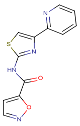	gskversion	O=C(Nc1nc(cs1)-c1ccccn1)c1ccno1	1,80	BCGset	
GSK1303831A	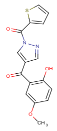	gskversion	COc1ccc(O)c(c1)C(=O)c1cnn(c1)C(=O)c1cccs1	1,80	BCGset	
GSK1352914A	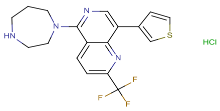	gskversion	FC(F)(F)c1ccc2c(ncc(-c3ccsc3)c2n1)N1CCCNCC1	1,80	BCGset	
GSK1667528A	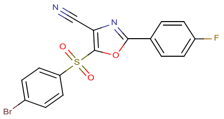	gskversion	Fc1ccc(cc1)-c1nc(C#N)c(o1)S(=O)(=O)c1ccc(Br)cc1	1,80	BCGset	
GSK1783693A	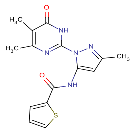	gskversion	Cc1cc(NC(=O)c2cccs2)n(n1)-c1nc(C)c(C)c(=O)[nH]1	1,80	BCGset	
GSK1783699A	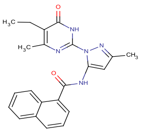	gskversion	CCc1c(C)nc([nH]c1=O)-n1nc(C)cc1NC(=O)c1cccc2ccccc12	1,80	BCGset	
GSK2059310A	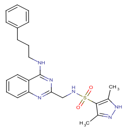	gskversion	Cc1n[nH]c(C)c1S(=O)(=O)NCc1nc(NCCCc2ccccc2)c2ccccc2n1	1,80	BCGset	TBset
SB-435634	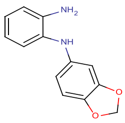	gskversion	Nc1ccccc1Nc1ccc2OCOc2c1	1,82	BCGset	TBset
GSK1042454A	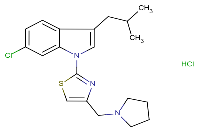	gskversion	CC(C)Cc1cn(-c2nc(CN3CCCC3)cs2)c2cc(Cl)ccc12	1,90	BCGset	
GSK1057729A	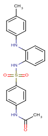	gskversion	CC(=O)Nc1ccc(cc1)S(=O)(=O)Nc1ccccc1Nc1ccc(C)cc1	1,90	BCGset	
GSK1188379A	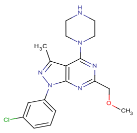	gskversion	COCc1nc(N2CCNCC2)c2c(C)nn(-c3cccc(Cl)c3)c2n1	1,90	BCGset	

GSK1744926A	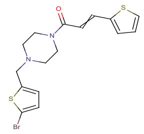	gskversion	Brc1ccc(CN2CCN(CC2)C(=O)C=Cc2cccs2)s1	1,90	BCGset	TBset
GSK1750922A	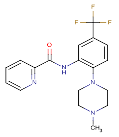	gskversion	CN1CCN(CC1)c1ccc(cc1NC(=O)c1ccccn1)C(F)(F)F	1,91	BCGset	TBset
GSK1022128A	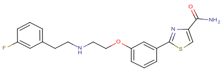	gskversion	NC(=O)c1csc(n1)-c1cccc(OCCNCCc2cccc(F)c2)c1	2,00	BCGset	
GSK1214892A	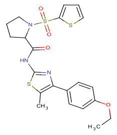	gskversion	CCOc1ccc(cc1)-c1nc(NC(=O)C2CCCN2S(=O)(=O)c2cccs2)sc1C	2,00	BCGset	
GSK1238596A	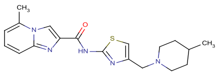	gskversion	CC1CCN(Cc2csc(NC(=O)c3cn4c(C)cccc4n3)n2)CC1	2,00	BCGset	
GSK140123A	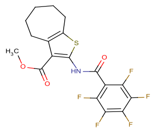	gskversion	COC(=O)c1c(NC(=O)c2c(F)c(F)c(F)c(F)c2F)sc2CCCCCc12	2,00	BCGset	
GSK1471133A	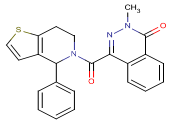	gskversion	Cn1nc(C(=O)N2CCc3sccc3C2c2ccccc2)c2ccccc2c1=O	2,00	BCGset	
GSK1540999A	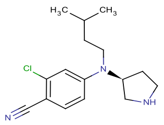	gskversion	CC(C)CCN([C@H]1CCNC1)c1ccc(C#N)c(Cl)c1	2,00	BCGset	
GSK1753353A	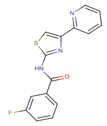	gskversion	Fc1cccc(c1)C(=O)Nc1nc(cs1)-c1ccccn1	2,00	BCGset	
GSK356463A	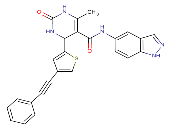	gskversion	CC1=C(C(NC(=O)N1)c1cc(cs1)C#Cc1ccccc1)C(=O)Nc1ccc2[nH]ncc2c1	2,00	BCGset	
SB-615575	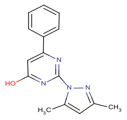	gskversion	Cc1cc(C)n(n1)-c1nc(O)cc(n1)-c1ccccc1	2,00	BCGset	
GSK957094A	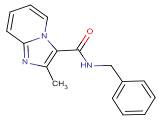	gskversion	Cc1nc2ccccn2c1C(=O)NCc1ccccc1	2,00	BCGset	TBset
GI250690X	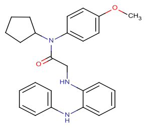	gskversion	COc1ccc(cc1)N(C1CCCC1)C(=O)CNc1ccccc1Nc1ccccc1	2,10	BCGset	

GSK1057079A	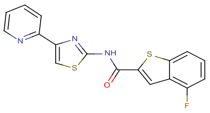	gskversion	Fc1cccc2sc(cc12)C(=O)Nc1nc(cs1)-c1ccccn1	2,10	BCGset	
GSK1277359A	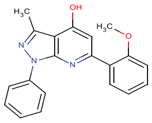	gskversion	COc1ccccc1-c1cc(O)c2c(C)nn(-c3ccccc3)c2n1	2,10	BCGset	
GW869933B	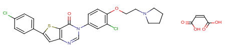	gskversion	Clc1ccc(cc1)-c1cc2ncn(-c3ccc(OCCN4CCCC4)c(Cl)c3)c(=O)c2s1	2,10	BCGset	
SB-381759	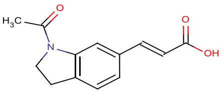	gskversion	CC(=O)N1CCc2ccc(\C=C\C(O)=O)cc12	2,10	BCGset	
GSK1829733A	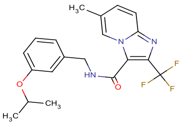	gskversion	CC(C)Oc1cccc(CNC(=O)c2c(nc3ccc(C)cn23)C(F)(F)F)c1	2,10	BCGset	TBset
BRL-24348PM-A	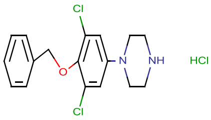	gskversion	Clc1cc(cc(Cl)c1OCc1ccccc1)N1CCNCC1	2,20	BCGset	
GSK1072523A	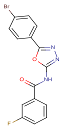	gskversion	Fc1cccc(c1)C(=O)Nc1nnc(o1)-c1ccc(Br)cc1	2,20	BCGset	
GSK547524A	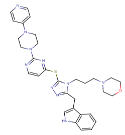	gskversion	C(CN1CCOCC1)Cn1c(Cc2c[nH]c3ccccc23)nnc1Sc1ccnc(n1)N1CCN(CC1)c1ccncc1	2,20	BCGset	
GSK695914A	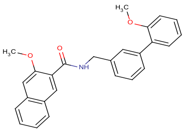	gskversion	COc1ccccc1-c1cccc(CNC(=O)c2cc3ccccc3cc2OC)c1	2,20	BCGset	TBset
GR135487X	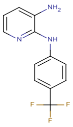	gskversion	Nc1cccnc1Nc1ccc(cc1)C(F)(F)F	2,24	BCGset	TBset
GI105268X	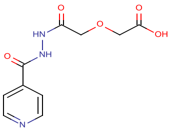	gskversion	OC(=O)COCC(=O)NNC(=O)c1ccncc1	2,30	BCGset	
GR119270B	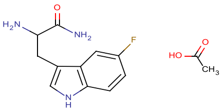	gskversion	NC(Cc1c[nH]c2ccc(F)cc12)C(N)=O	2,30	BCGset	
GSK1073692A	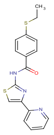	gskversion	CCSc1ccc(cc1)C(=O)Nc1nc(cs1)-c1ccccn1	2,30	BCGset	

GSK1215270A	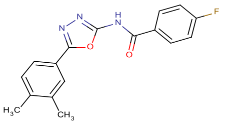	gskversion	Cc1ccc(cc1C)-c1nnc(NC(=O)c2ccc(F)cc2)o1	2,30	BCGset	
GSK2200174A	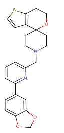	gskversion	C(N1CCC2(CC1)OCCc1sccc21)c1cccc(n1)-c1ccc2OCOc2c1	2,30	BCGset	
GSK735791A	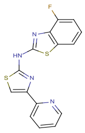	gskversion	Fc1cccc2sc(Nc3nc(cs3)-c3ccccn3)nc12	2,30	BCGset	
SB-277574	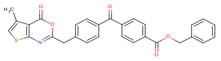	gskversion	Cc1csc2nc(Cc3ccc(cc3)C(=O)c3ccc(cc3)C(=O)OCc3ccccc3)oc(=O)c12	2,30	BCGset	
SB-439950	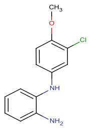	gskversion	COc1ccc(Nc2ccccc2N)cc1Cl	2,30	BCGset	
BRL-7940SA	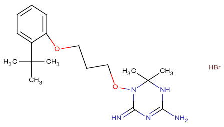	gskversion	CC(C)(C)c1ccccc1OCCCON1C(=N)N=C(N)NC1(C)C	2,30	BCGset	TBset
GSK124576A	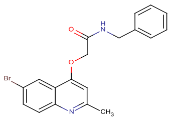	gskversion	Cc1cc(OCC(=O)NCc2ccccc2)c2cc(Br)ccc2n1	2,30	BCGset	TBset
GSK735816A	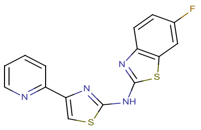	gskversion	Fc1ccc2nc(Nc3nc(cs3)-c3ccccn3)sc2c1	2,30	BCGset	TBset
BRL-8088SA	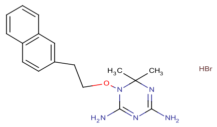	gskversion	CC1(C)N=C(N)N=C(N)N1OCCc1ccc2ccccc2c1	2,32	BCGset	TBset
BRL-10988SA	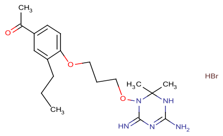	gskversion	CCCc1cc(ccc1OCCCON1C(=N)N=C(N)NC1(C)C)C(C)=O	2,34	BCGset	TBset
CCI7967	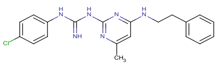	gskversion	Cc1cc(NCCc2ccccc2)nc(NC(=N)Nc2ccc(Cl)cc2)n1	2,37	BCGset	TBset
GSK1839939A	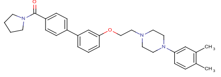	gskversion	Cc1ccc(cc1C)N1CCN(CCOc2cccc(c2)-c2ccc(cc2)C(=O)N2CCCC2)CC1	2,40	BCGset	
GSK441195A	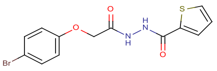	gskversion	Brc1ccc(OCC(=O)NNC(=O)c2cccs2)cc1	2,40	BCGset	

GSK547489A	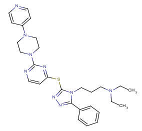	gskversion	CCN(CC)CCCn1c(Sc2ccnc(n2)N2CCN(CC2)c2ccncc2)nnc1-c1ccccc1	2,40	BCGset	
GSK547541A	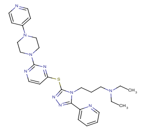	gskversion	CCN(CC)CCCn1c(Sc2ccnc(n2)N2CCN(CC2)c2ccncc2)nnc1-c1ccccn1	2,40	BCGset	
GSK728157A	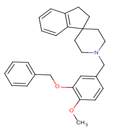	gskversion	COc1ccc(CN2CCC3(CCc4ccccc34)CC2)cc1OCc1ccccc1	2,40	BCGset	
GSK854007A	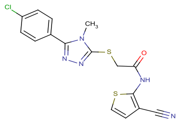	gskversion	Cn1c(SCC(=O)Nc2sccc2C#N)nnc1-c1ccc(Cl)cc1	2,40	BCGset	
GSK810037A	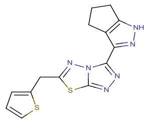	gskversion	C(c1cccs1)c1nn2c(nnc2s1)-c1n[nH]c2CCCc12	2,40	BCGset	TBset
GSK254610A	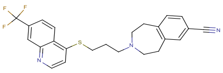	gskversion	FC(F)(F)c1ccc2c(SCCCN3CCc4ccc(cc4CC3)C#N)ccnc2c1	2,40	BCGset	TBset
GSK1274131A	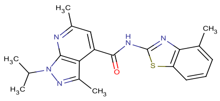	gskversion	CC(C)n1nc(C)c2c(cc(C)nc12)C(=O)Nc1nc2c(C)cccc2s1	2,50	BCGset	
GSK1817710A	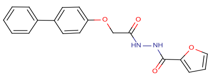	gskversion	O=C(COc1ccc(cc1)-c1ccccc1)NNC(=O)c1ccco1	2,50	BCGset	
GSK222392A	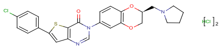	gskversion	Clc1ccc(cc1)-c1cc2ncn(-c3ccc4O[C@@H](CN5CCCC5)COc4c3)c(=O)c2s1	2,50	BCGset	
GSK641592A	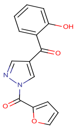	gskversion	Oc1ccccc1C(=O)c1cnn(c1)C(=O)c1ccco1	2,50	BCGset	
GW611920X	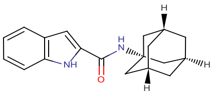	gskversion	O=C(NC12CC3CC(CC(C3)C1)C2)c1cc2ccccc2[nH]1	2,50	BCGset	
GSK426032A	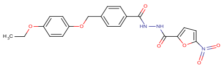	gskversion	CCOc1ccc(OCc2ccc(cc2)C(=O)NNC(=O)c2ccc(o2)N(=O)=O)cc1	2,50	BCGset	TBset
GI259770X	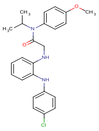	gskversion	COc1ccc(cc1)N(C(C)C)C(=O)CNc1ccccc1Nc1ccc(Cl)cc1	2,60	BCGset	

GSK1517183A	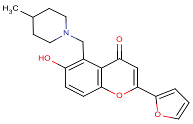	gskversion	CC1CCN(Cc2c(O)ccc3oc(cc(=O)c23)-c2ccco2)CC1	2,60	BCGset	
GSK489489A	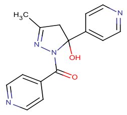	gskversion	CC1=NN(C(=O)c2ccncc2)C(O)(C1)c1ccncc1	2,60	BCGset	
GSK547496A	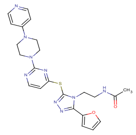	gskversion	CC(=O)NCCn1c(Sc2ccnc(n2)N2CCN(CC2)c2ccncc2)nnc1-c1ccco1	2,60	BCGset	
SB-779796-V	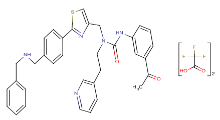	gskversion	CC(=O)c1cccc(NC(=O)N(CCc2cccnc2)Cc2csc(n2)-c2ccc(CNCc3ccccc3)cc2)c1	2,60	BCGset	
GR223839X	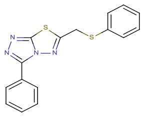	gskversion	C(Sc1ccccc1)c1nn2c(nnc2s1)-c1ccccc1	2,69	BCGset	TBset
GI205671X	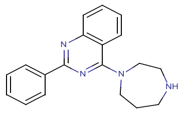	gskversion	C1CNCCN(C1)c1nc(nc2ccccc12)-c1ccccc1	2,70	BCGset	
GSK1645397A	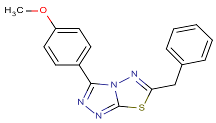	gskversion	COc1ccc(cc1)-c1nnc2sc(Cc3ccccc3)nn12	2,70	BCGset	
GSK1717126A	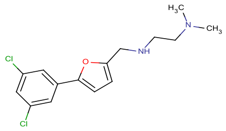	gskversion	CN(C)CCNCc1ccc(o1)-c1cc(Cl)cc(Cl)c1	2,70	BCGset	
GSK1783677A	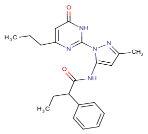	gskversion	CCCc1cc(=O)[nH]c(n1)-n1nc(C)cc1NC(=O)C(CC)c1ccccc1	2,70	BCGset	
GSK2200168A	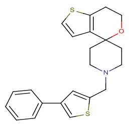	gskversion	C(N1CCC2(CC1)OCCc1sccc21)c1cc(cs1)-c1ccccc1	2,70	BCGset	
GSK547512A	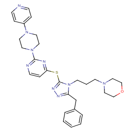	gskversion	C(CN1CCOCC1)Cn1c(Cc2ccccc2)nnc1Sc1ccnc(n1)N1CCN(CC1)c1ccncc1	2,70	BCGset	
SB-255624-M	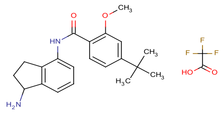	gskversion	COc1cc(ccc1C(=O)Nc1cccc2C(N)CCc12)C(C)(C)C	2,70	BCGset	
GSK861337A	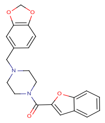	gskversion	O=C(N1CCN(Cc2ccc3OCOc3c2)CC1)c1cc2ccccc2o1	2,70	BCGset	TBset

GSK735826A	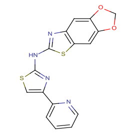	gskversion	C1Oc2cc3nc(Nc4nc(cs4)-c4ccccn4)sc3cc2O1	2,70	BCGset	TBset
GSK124945A	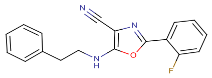	gskversion	Fc1ccccc1-c1nc(C#N)c(NCCc2ccccc2)o1	2,79	BCGset	TBset
GSK829969A	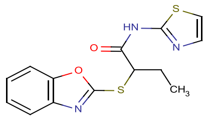	gskversion	CCC(Sc1nc2ccccc2o1)C(=O)Nc1nccs1	2,79	BCGset	TBset
AH24048X	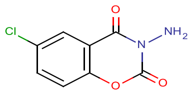	gskversion	Nn1c(=O)oc2ccc(Cl)cc2c1=O	2,80	BCGset	
GSK1048034A	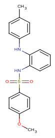	gskversion	COc1ccc(cc1)S(=O)(=O)Nc1ccccc1Nc1ccc(C)cc1	2,80	BCGset	
GSK1735958A	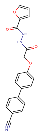	gskversion	O=C(COc1ccc(cc1)-c1ccc(cc1)C#N)NNC(=O)c1ccco1	2,80	BCGset	
GSK1783702A	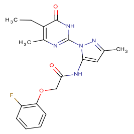	gskversion	CCc1c(C)nc([nH]c1=O)-n1nc(C)cc1NC(=O)COc1ccccc1F	2,80	BCGset	
GSK2200151A	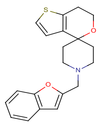	gskversion	C(N1CCC2(CC1)OCCc1sccc21)c1cc2ccccc2o1	2,80	BCGset	
GSK237560A	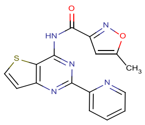	gskversion	Cc1cc(no1)C(=O)Nc1nc(nc2ccsc12)-c1ccccn1	2,80	BCGset	
GSK270677A	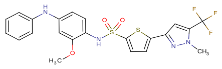	gskversion	COc1cc(Nc2ccccc2)ccc1NS(=O)(=O)c1ccc(s1)-c1cc(n(C)n1)C(F)(F)F	2,80	BCGset	
GSK346583A	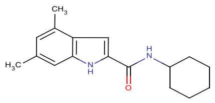	gskversion	Cc1cc(C)c2cc([nH]c2c1)C(=O)NC1CCCCC1	2,80	BCGset	
GSK370365A	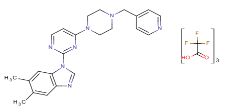	gskversion	Cc1cc2ncn(-c3nccc(n3)N3CCN(Cc4ccncc4)CC3)c2cc1C	2,80	BCGset	
GSK547498A	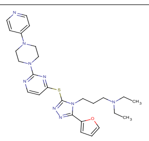	gskversion	CCN(CC)CCCn1c(Sc2ccnc(n2)N2CCN(CC2)c2ccncc2)nnc1-c1ccco1	2,80	BCGset	

GSK547500A	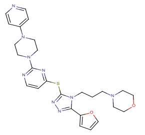	gskversion	C(CN1CCOCC1)Cn1c(Sc2ccnc(n2)N2CCN(CC2)c2ccncc2)nnc1-c1ccco1	2,80	BCGset	
GSK547523A	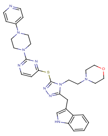	gskversion	C(Cn1c(Cc2c[nH]c3ccccc23)nnc1Sc1ccnc(n1)N1CCN(CC1)c1ccncc1)N1CCOCC1	2,80	BCGset	
GSK857710A	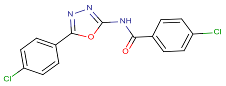	gskversion	Clc1ccc(cc1)C(=O)Nc1nnc(o1)-c1ccc(Cl)cc1	2,80	BCGset	
GSK975840A	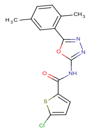	gskversion	Cc1ccc(C)c(c1)-c1nnc(NC(=O)c2ccc(Cl)s2)o1	2,80	BCGset	
GSK1859936A	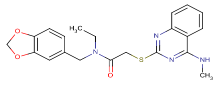	gskversion	CCN(Cc1ccc2OCOc2c1)C(=O)CSc1nc(NC)c2ccccc2n1	2,80	BCGset	TBset
GSK1742694A	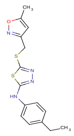	gskversion	CCc1ccc(Nc2nnc(SCc3cc(C)on3)s2)cc1	2,80	BCGset	TBset
GSK134227A	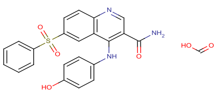	gskversion	NC(=O)c1cnc2ccc(cc2c1Nc1ccc(O)cc1)S(=O)(=O)c1ccccc1	2,90	BCGset	
GSK1669247A	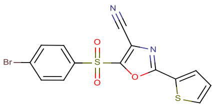	gskversion	Brc1ccc(cc1)S(=O)(=O)c1oc(nc1C#N)-c1cccs1	2,90	BCGset	
GSK1826054A	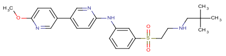	gskversion	COc1ccc(cn1)-c1ccc(Nc2cccc(c2)S(=O)(=O)CCNCC(C)(C)C)nc1	2,90	BCGset	
GSK547510A	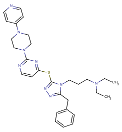	gskversion	CCN(CC)CCCn1c(Cc2ccccc2)nnc1Sc1ccnc(n1)N1CCN(CC1)c1ccncc1	2,90	BCGset	
GSK884710A	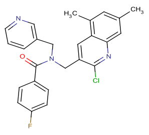	gskversion	Cc1cc(C)c2cc(CN(Cc3cccnc3)C(=O)c3ccc(F)cc3)c(Cl)nc2c1	2,90	BCGset	
GSK498315A	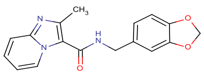	gskversion	Cc1nc2ccccn2c1C(=O)NCc1ccc2OCOc2c1	2,90	BCGset	TBset
GSK1996236A	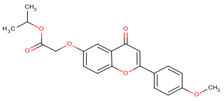	gskversion	COc1ccc(cc1)-c1cc(=O)c2cc(OCC(=O)OC(C)C)ccc2o1	2,95	BCGset	TBset

GW360240X	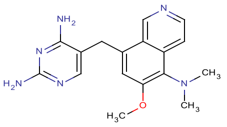	gskversion	COc1cc(Cc2cnc(N)nc2N)c2cnccc2c1N(C)C	2,99	BCGset	TBset
GSK1188072A	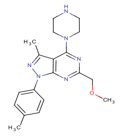	gskversion	COCc1nc(N2CCNCC2)c2c(C)nn(-c3ccc(C)cc3)c2n1	3,00	BCGset	
GSK270671A	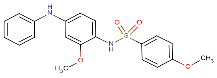	gskversion	COc1ccc(cc1)S(=O)(=O)Nc1ccc(Nc2ccccc2)cc1OC	3,00	BCGset	
GSK273492A	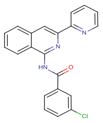	gskversion	Clc1cccc(c1)C(=O)Nc1nc(cc2ccccc12)-c1ccccn1	3,00	BCGset	
GSK293721A	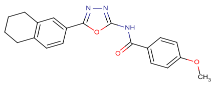	gskversion	COc1ccc(cc1)C(=O)Nc1nnc(o1)-c1ccc2CCCCc2c1	3,00	BCGset	
GSK359447A	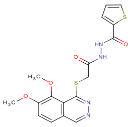	gskversion	COc1ccc2cnnc(SCC(=O)NNC(=O)c3cccs3)c2c1OC	3,00	BCGset	
GSK736709A	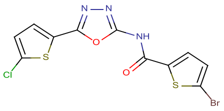	gskversion	Clc1ccc(s1)-c1nnc(NC(=O)c2ccc(Br)s2)o1	3,00	BCGset	
GSK768470A	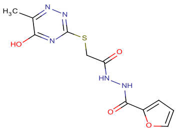	gskversion	Cc1nnc(SCC(=O)NNC(=O)c2ccco2)nc1O	3,00	BCGset	
GSK920656A	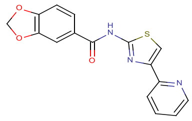	gskversion	O=C(Nc1nc(cs1)-c1ccccn1)c1ccc2OCOc2c1	3,00	BCGset	
GSK975852A	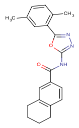	gskversion	Cc1ccc(C)c(c1)-c1nnc(NC(=O)c2ccc3CCCCc3c2)o1	3,00	BCGset	
SB-650816	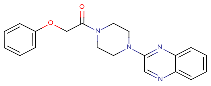	gskversion	O=C(COc1ccccc1)N1CCN(CC1)c1cnc2ccccc2n1	3,09	BCGset	TBset
GR156016X	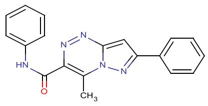	gskversion	Cc1c(nnc2cc(nn12)-c1ccccc1)C(=O)Nc1ccccc1	3,10	BCGset	
GSK1093563A	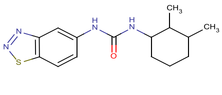	gskversion	CC1CCCC(NC(=O)Nc2ccc3snnc3c2)C1C	3,10	BCGset	

GSK1222186A	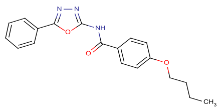	gskversion	CCCCOc1ccc(cc1)C(=O)Nc1nnc(o1)-c1ccccc1	3,10	BCGset	
GSK1692825A	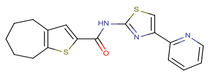	gskversion	O=C(Nc1nc(cs1)-c1ccccn1)c1cc2CCCCCc2s1	3,10	BCGset	
GSK1825996A	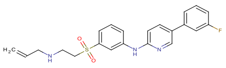	gskversion	Fc1cccc(c1)-c1ccc(Nc2cccc(c2)S(=O)(=O)CCNCC=C)nc1	3,10	BCGset	
GSK1839929A	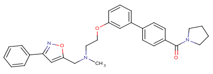	gskversion	CN(CCOc1cccc(c1)-c1ccc(cc1)C(=O)N1CCCC1)Cc1cc(no1)-c1ccccc1	3,10	BCGset	
GSK497472A	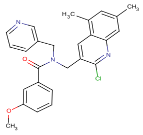	gskversion	COc1cccc(c1)C(=O)N(Cc1cccnc1)Cc1cc2c(C)cc(C)cc2nc1Cl	3,10	BCGset	
GSK904260A	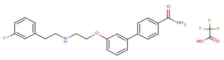	gskversion	NC(=O)c1ccc(cc1)-c1cccc(OCCNCCc2cccc(F)c2)c1	3,10	BCGset	
SB-797843-A	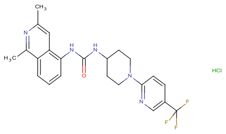	gskversion	Cc1cc2c(NC(=O)NC3CCN(CC3)c3ccc(cn3)C(F)(F)F)cccc2c(C)n1	3,10	BCGset	
SKF-67461	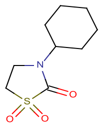	gskversion	O=C1N(CCS1(=O)=O)C1CCCCC1	3,10	BCGset	
GSK1829674A	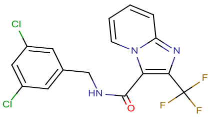	gskversion	FC(F)(F)c1nc2ccccn2c1C(=O)NCc1cc(Cl)cc(Cl)c1	3,10	BCGset	TBset
GSK762874A	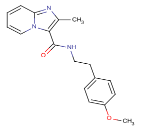	gskversion	COc1ccc(CCNC(=O)c2c(C)nc3ccccn23)cc1	3,10	BCGset	TBset
BRL-10143SA	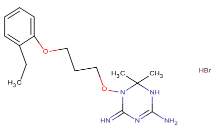	gskversion	CCc1ccccc1OCCCON1C(=N)N=C(N)NC1(C)C	3,20	BCGset	TBset
GSK353071A	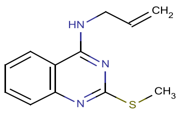	gskversion	CSc1nc(NCC=C)c2ccccc2n1	3,20	BCGset	TBset
GSK1296323A	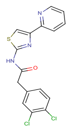	gskversion	Clc1ccc(CC(=O)Nc2nc(cs2)-c2ccccn2)cc1Cl	3,20	BCGset	

GSK1600166A	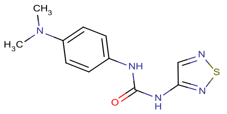	gskversion	CN(C)c1ccc(NC(=O)Nc2cnsn2)cc1	3,20	BCGset	
GSK552667A	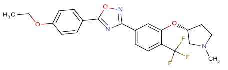	gskversion	CCOc1ccc(cc1)-c1nc(no1)-c1ccc(c(O[C@@H]2CCN(C)C2)c1)C(F)(F)F	3,20	BCGset	
GSK1026396A	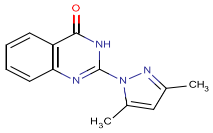	gskversion	Cc1cc(C)n(n1)-c1nc2ccccc2c(=O)[nH]1	3,30	BCGset	
GSK1252526A	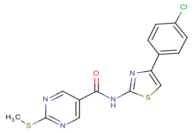	gskversion	CSc1ncc(cn1)C(=O)Nc1nc(cs1)-c1ccc(Cl)cc1	3,30	BCGset	
GSK1618054A	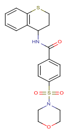	gskversion	O=C(NC1CCSc2ccccc12)c1ccc(cc1)S(=O)(=O)N1CCOCC1	3,30	BCGset	
GSK1685856A	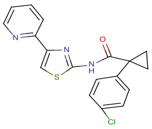	gskversion	Clc1ccc(cc1)C1(CC1)C(=O)Nc1nc(cs1)-c1ccccn1	3,30	BCGset	
GSK1783703A	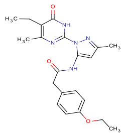	gskversion	CCOc1ccc(CC(=O)Nc2cc(C)nn2-c2nc(C)c(CC)c(=O)[nH]2)cc1	3,30	BCGset	
GSK547528A	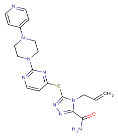	gskversion	NC(=O)c1nnc(Sc2ccnc(n2)N2CCN(CC2)c2ccncc2)n1CC=C	3,30	BCGset	
GSK813524A	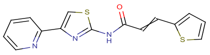	gskversion	O=C(Nc1nc(cs1)-c1ccccn1)C=Cc1cccs1	3,30	BCGset	
GSK816677A	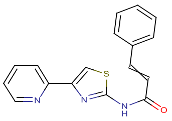	gskversion	O=C(Nc1nc(cs1)-c1ccccn1)C=Cc1ccccc1	3,30	BCGset	
GSK921452A	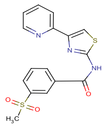	gskversion	CS(=O)(=O)c1cccc(c1)C(=O)Nc1nc(cs1)-c1ccccn1	3,30	BCGset	
GSK1829671A	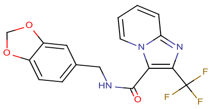	gskversion	FC(F)(F)c1nc2ccccn2c1C(=O)NCc1ccc2OCOc2c1	3,30	BCGset	TBset
GW857165X	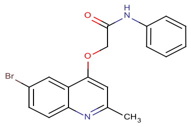	gskversion	Cc1cc(OCC(=O)Nc2ccccc2)c2cc(Br)ccc2n1	3,30	BCGset	TBset

GSK847913A	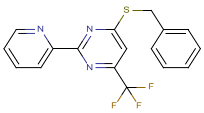	gskversion	FC(F)(F)c1cc(SCc2ccccc2)nc(n1)-c1ccccn1	3,30	BCGset	TBset
GSK1220653A	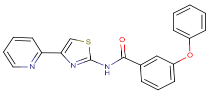	gskversion	O=C(Nc1nc(cs1)-c1ccccn1)c1cccc(Oc2ccccc2)c1	3,40	BCGset	
GSK1221860A	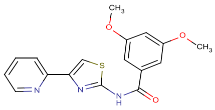	gskversion	COc1cc(OC)cc(c1)C(=O)Nc1nc(cs1)-c1ccccn1	3,40	BCGset	
GSK1646210B	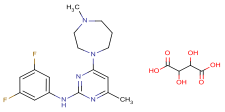	gskversion	CN1CCCN(CC1)c1cc(C)nc(Nc2cc(F)cc(F)c2)n1	3,40	BCGset	
GSK353496A	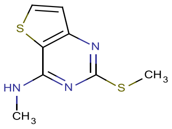	gskversion	CNc1nc(SC)nc2ccsc12	3,40	BCGset	TBset
GSK994258A	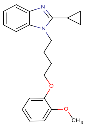	gskversion	COc1ccccc1OCCCCn1c(nc2ccccc12)C1CC1	3,40	BCGset	TBset
GSK1829728A	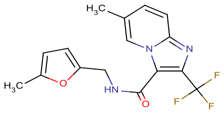	gskversion	Cc1ccc(CNC(=O)c2c(nc3ccc(C)cn23)C(F)(F)F)o1	3,43	BCGset	TBset
GSK547481A	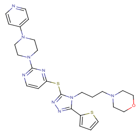	gskversion	C(CN1CCOCC1)Cn1c(Sc2ccnc(n2)N2CCN(CC2)c2ccncc2)nnc1-c1cccs1	3,43	BCGset	TBset
GSK1372431A	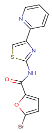	gskversion	Brc1ccc(o1)C(=O)Nc1nc(cs1)-c1ccccn1	3,50	BCGset	
GSK2188570A	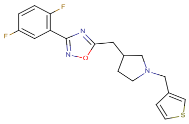	gskversion	Fc1ccc(F)c(c1)-c1noc(CC2CCN(Cc3ccsc3)C2)n1	3,50	BCGset	
GSK1365028A	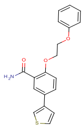	gskversion	NC(=O)c1cc(ccc1OCCOc1ccccc1)-c1ccsc1	3,50	BCGset	TBset
GSK920684A	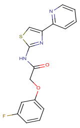	gskversion	Fc1cccc(OCC(=O)Nc2nc(cs2)-c2ccccn2)c1	3,51	BCGset	TBset
BRL-51091AM	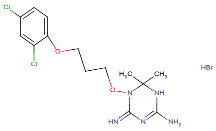	gskversion	CC1(C)NC(N)=NC(=N)N1OCCCOc1ccc(Cl)cc1Cl	3,55	BCGset	TBset

GR201245X	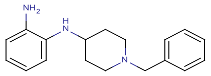	gskversion	Nc1ccccc1NC1CCN(Cc2ccccc2)CC1	3,60	BCGset	
GSK1046121A	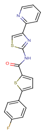	gskversion	Fc1ccc(cc1)-c1ccc(s1)C(=O)Nc1nc(cs1)-c1ccccn1	3,60	BCGset	
GSK1235509A	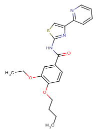	gskversion	CCCCOc1ccc(cc1OCC)C(=O)Nc1nc(cs1)-c1ccccn1	3,60	BCGset	
GSK1447646A	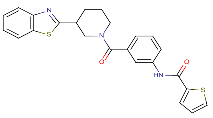	gskversion	O=C(Nc1cccc(c1)C(=O)N1CCCC(C1)c1nc2ccccc2s1)c1cccs1	3,60	BCGset	
GSK1452001A	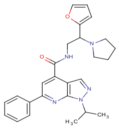	gskversion	CC(C)n1ncc2c(cc(nc12)-c1ccccc1)C(=O)NCC(N1CCCC1)c1ccco1	3,60	BCGset	
GSK1783646A	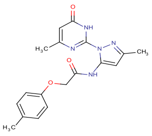	gskversion	Cc1cc(NC(=O)COc2ccc(C)cc2)n(n1)-c1nc(C)cc(=O)[nH]1	3,60	BCGset	
GSK1825944A	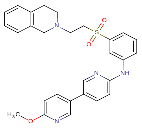	gskversion	COc1ccc(cn1)-c1ccc(Nc2cccc(c2)S(=O)(=O)CCN2CCc3ccccc3C2)nc1	3,60	BCGset	
GSK1825993A	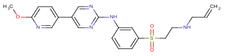	gskversion	COc1ccc(cn1)-c1cnc(Nc2cccc(c2)S(=O)(=O)CCNCC=C)nc1	3,60	BCGset	
GSK490197A	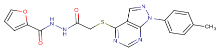	gskversion	Cc1ccc(cc1)-n1ncc2c(SCC(=O)NNC(=O)c3ccco3)ncnc12	3,60	BCGset	
GSK662563A	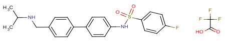	gskversion	CC(C)NCc1ccc(cc1)-c1ccc(NS(=O)(=O)c2ccc(F)cc2)cc1	3,60	BCGset	
GSK817365A	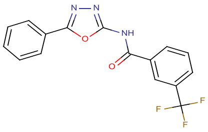	gskversion	FC(F)(F)c1cccc(c1)C(=O)Nc1nnc(o1)-c1ccccc1	3,60	BCGset	
GSK921639A	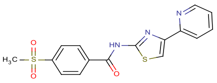	gskversion	CS(=O)(=O)c1ccc(cc1)C(=O)Nc1nc(cs1)-c1ccccn1	3,60	BCGset	
GW781990X	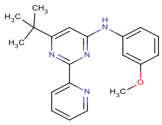	gskversion	COc1cccc(Nc2cc(nc(n2)-c2ccccn2)C(C)(C)C)c1	3,60	BCGset	

GSK1051654A	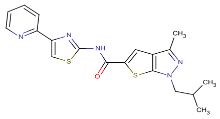	gskversion	CC(C)Cn1nc(C)c2cc(sc12)C(=O)Nc1nc(cs1)-c1ccccn1	3,70	BCGset	
GSK1073731A	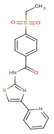	gskversion	CCS(=O)(=O)c1ccc(cc1)C(=O)Nc1nc(cs1)-c1ccccn1	3,70	BCGset	
GSK1104201A	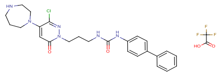	gskversion	Clc1nn(CCCNC(=O)Nc2ccc(cc2)-c2ccccc2)c(=O)cc1N1CCCNCC1	3,70	BCGset	
GSK112532A	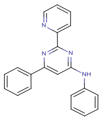	gskversion	N(c1ccccc1)c1cc(nc(n1)-c1ccccn1)-c1ccccc1	3,70	BCGset	
GSK1215273A	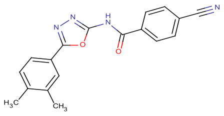	gskversion	Cc1ccc(cc1C)-c1nnc(NC(=O)c2ccc(cc2)C#N)o1	3,70	BCGset	
GSK1953199A	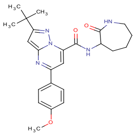	gskversion	COc1ccc(cc1)-c1cc(C(=O)NC2CCCCNC2=O)n2nc(cc2n1)C(C)(C)C	3,70	BCGset	
GSK415581A	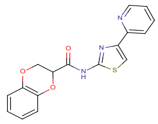	gskversion	O=C(Nc1nc(cs1)-c1ccccn1)C1COc2ccccc2O1	3,70	BCGset	
GSK621353A	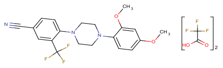	gskversion	COc1ccc(N2CCN(CC2)c2ccc(cc2C(F)(F)F)C#N)c(OC)c1	3,70	BCGset	
SB-255019-M	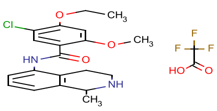	gskversion	CCOc1cc(OC)c(cc1Cl)C(=O)Nc1cccc2C(C)NCCc12	3,70	BCGset	
SB-516933	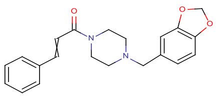	gskversion	O=C(C=Cc1ccccc1)N1CCN(Cc2ccc3OCOc3c2)CC1	3,70	BCGset	TBset
GSK2200157A	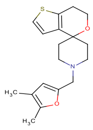	gskversion	Cc1cc(CN2CCC3(CC2)OCCc2sccc32)oc1C	3,70	BCGset	TBset
GSK798463A	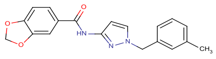	gskversion	Cc1cccc(Cn2ccc(NC(=O)c3ccc4OCOc4c3)n2)c1	3,70	BCGset	TBset
GSK385518A	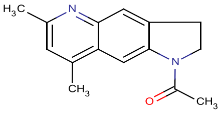	gskversion	CC(=O)N1CCc2cc3nc(C)cc(C)c3cc12	3,70	BCGset	TBset

GSK2043267A	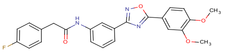	gskversion	COc1ccc(cc1OC)-c1nc(no1)-c1cccc(NC(=O)Cc2ccc(F)cc2)c1	3,70	BCGset	TBset
GSK1072678A	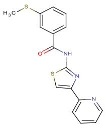	gskversion	CSc1cccc(c1)C(=O)Nc1nc(cs1)-c1ccccn1	3,72	BCGset	TBset
BRL-8903SA	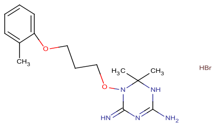	gskversion	Cc1ccccc1OCCCON1C(=N)N=C(N)NC1(C)C	3,76	BCGset	TBset
GSK1783672A	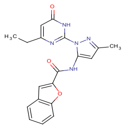	gskversion	CCc1cc(=O)[nH]c(n1)-n1nc(C)cc1NC(=O)c1cc2ccccc2o1	3,80	BCGset	
GSK1783705A	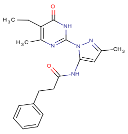	gskversion	CCc1c(C)nc([nH]c1=O)-n1nc(C)cc1NC(=O)CCc1ccccc1	3,80	BCGset	
GSK433921A	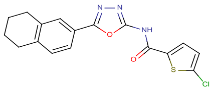	gskversion	Clc1ccc(s1)C(=O)Nc1nnc(o1)-c1ccc2CCCCc2c1	3,80	BCGset	
GSK452659A	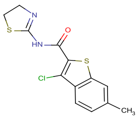	gskversion	Cc1ccc2c(Cl)c(sc2c1)C(=O)NC1=NCCS1	3,80	BCGset	
SB-826303	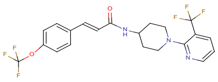	gskversion	FC(F)(F)Oc1ccc(\C=C\C(=O)NC2CCN(CC2)c2ncccc2C(F)(F)F)cc1	3,80	BCGset	
GSK1829727A	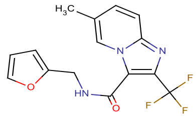	gskversion	Cc1ccc2nc(c(C(=O)NCc3ccco3)n2c1)C(F)(F)F	3,89	BCGset	TBset
GSK1188386A	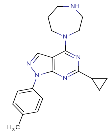	gskversion	Cc1ccc(cc1)-n1ncc2c(nc(nc12)C1CC1)N1CCCNCC1	3,90	BCGset	
GSK1213678A	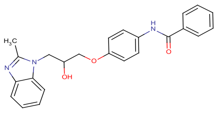	gskversion	Cc1nc2ccccc2n1CC(O)COc1ccc(NC(=O)c2ccccc2)cc1	3,90	BCGset	
GSK1239840A	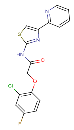	gskversion	Fc1ccc(OCC(=O)Nc2nc(cs2)-c2ccccn2)c(Cl)c1	3,90	BCGset	
GSK130507A	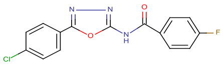	gskversion	Fc1ccc(cc1)C(=O)Nc1nnc(o1)-c1ccc(Cl)cc1	3,90	BCGset	

GSK143123A	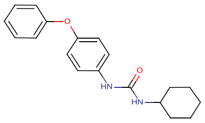	gskversion	O=C(NC1CCCCC1)Nc1ccc(Oc2ccccc2)cc1	3,90	BCGset	
GSK1583058A	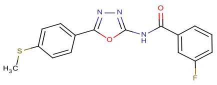	gskversion	CSc1ccc(cc1)-c1nnc(NC(=O)c2cccc(F)c2)o1	3,90	BCGset	
GSK1846201A	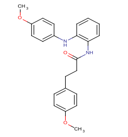	gskversion	COc1ccc(CCC(=O)Nc2ccccc2Nc2ccc(OC)cc2)cc1	3,90	BCGset	
GSK2149528A	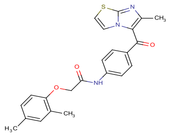	gskversion	Cc1nc2sccn2c1C(=O)c1ccc(NC(=O)COc2ccc(C)cc2C)cc1	3,90	BCGset	
GSK942642A	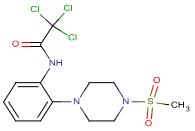	gskversion	CS(=O)(=O)N1CCN(CC1)c1ccccc1NC(=O)C(Cl)(Cl)Cl	3,90	BCGset	
GW655133X	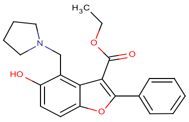	gskversion	CCOC(=O)c1c(oc2ccc(O)c(CN3CCCC3)c12)-c1ccccc1	3,90	BCGset	
GSK116466A	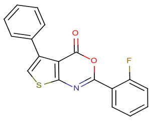	gskversion	Fc1ccccc1-c1nc2scc(-c3ccccc3)c2c(=O)o1	4,00	BCGset	
GSK1553808A	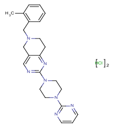	gskversion	Cc1ccccc1CN1CCc2nc(ncc2C1)N1CCN(CC1)c1ncccn1	4,00	BCGset	
GSK515917A	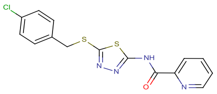	gskversion	Clc1ccc(CSc2nnc(NC(=O)c3ccccn3)s2)cc1	4,00	BCGset	
GSK735437A	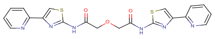	gskversion	O=C(COCC(=O)Nc1nc(cs1)-c1ccccn1)Nc1nc(cs1)-c1ccccn1	4,00	BCGset	
GSK870970A	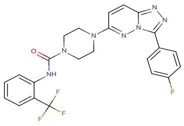	gskversion	Fc1ccc(cc1)-c1nnc2ccc(nn12)N1CCN(CC1)C(=O)Nc1ccccc1C(F)(F)F	4,00	BCGset	
GSK975810A	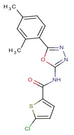	gskversion	Cc1ccc(-c2nnc(NC(=O)c3ccc(Cl)s3)o2)c(C)c1	4,00	BCGset	
SB-248988	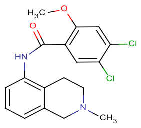	gskversion	COc1cc(Cl)c(Cl)cc1C(=O)Nc1cccc2CN(C)CCc12	4,00	BCGset	

SB-293671-A	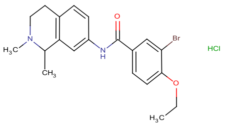	gskversion	CCOc1ccc(cc1Br)C(=O)Nc1ccc2CCN(C)C(C)c2c1	4,00	BCGset	
GSK237561A	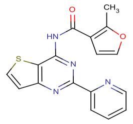	gskversion	Cc1occc1C(=O)Nc1nc(nc2ccsc12)-c1ccccn1	4,00	BCGset	TBset
GSK262906A	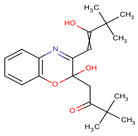	gskversion	CC(C)(C)C(=O)CC1(O)Oc2ccccc2N=C1C=C(O)C(C)(C)C	4,03	BCGset	TBset
GR153167X	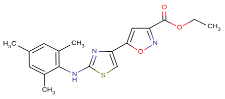	gskversion	CCOC(=O)c1cc(on1)-c1csc(Nc2c(C)cc(C)cc2C)n1	4,07	BCGset	TBset
GSK1013542A	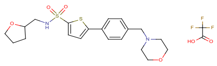	gskversion	O=S(=O)(NCC1CCCO1)c1ccc(s1)-c1ccc(CN2CCOCC2)cc1	4,10	BCGset	
GSK1079325A	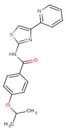	gskversion	CC(C)Oc1ccc(cc1)C(=O)Nc1nc(cs1)-c1ccccn1	4,10	BCGset	
GSK1188389A	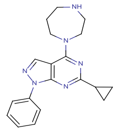	gskversion	C1CC1c1nc(N2CCCNCC2)c2cnn(-c3ccccc3)c2n1	4,10	BCGset	
GSK1216252A	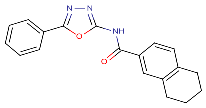	gskversion	O=C(Nc1nnc(o1)-c1ccccc1)c1ccc2CCCCc2c1	4,10	BCGset	
GSK963391A	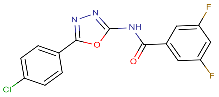	gskversion	Fc1cc(F)cc(c1)C(=O)Nc1nnc(o1)-c1ccc(Cl)cc1	4,10	BCGset	
GSK975842A	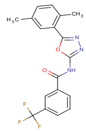	gskversion	Cc1ccc(C)c(c1)-c1nnc(NC(=O)c2cccc(c2)C(F)(F)F)o1	4,10	BCGset	
GR51104X	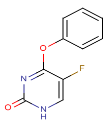	gskversion	Fc1c[nH]c(=O)nc1Oc1ccccc1	4,20	BCGset	
GSK1214451A	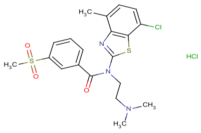	gskversion	CN(C)CCN(C(=O)c1cccc(c1)S(C)(=O)=O)c1nc2c(C)ccc(Cl)c2s1	4,20	BCGset	
GSK148788A	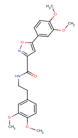	gskversion	COc1ccc(CCNC(=O)c2cc(on2)-c2ccc(OC)c(OC)c2)cc1OC	4,20	BCGset	

GSK1752829A	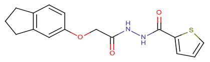	gskversion	O=C(COc1ccc2CCCc2c1)NNC(=O)c1cccs1	4,20	BCGset	
GSK186067A	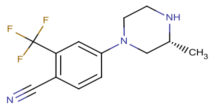	gskversion	C[C@@H]1CN(CCN1)c1ccc(C#N)c(c1)C(F)(F)F	4,20	BCGset	
GSK1893692A	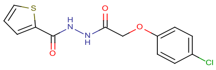	gskversion	Clc1ccc(OCC(=O)NNC(=O)c2cccs2)cc1	4,20	BCGset	
GSK267280A	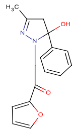	gskversion	CC1=NN(C(=O)c2ccco2)C(O)(C1)c1ccccc1	4,20	BCGset	
GSK547490A	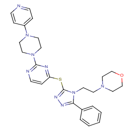	gskversion	C(Cn1c(Sc2ccnc(n2)N2CCN(CC2)c2ccncc2)nnc1-c1ccccc1)N1CCOCC1	4,20	BCGset	
SB-826304	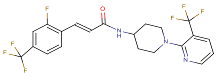	gskversion	Fc1cc(ccc1\C=C\C(=O)NC1CCN(CC1)c1ncccc1C(F)(F)F)C(F)(F)F	4,20	BCGset	
GSK1985270A	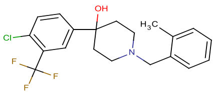	gskversion	Cc1ccccc1CN1CCC(O)(CC1)c1ccc(Cl)c(c1)C(F)(F)F	4,20	BCGset	TBset
GSK2032710A	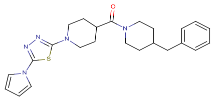	gskversion	O=C(C1CCN(CC1)c1nnc(s1)-n1cccc1)N1CCC(Cc2ccccc2)CC1	4,20	BCGset	TBset
GSK1079320A	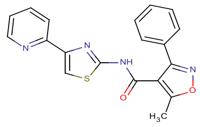	gskversion	Cc1onc(c1C(=O)Nc1nc(cs1)-c1ccccn1)-c1ccccc1	4,30	BCGset	
GSK1103969A	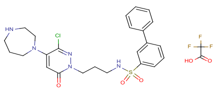	gskversion	Clc1nn(CCCNS(=O)(=O)c2cccc(c2)-c2ccccc2)c(=O)cc1N1CCCNCC1	4,30	BCGset	
GSK1306952A	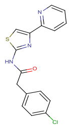	gskversion	Clc1ccc(CC(=O)Nc2nc(cs2)-c2ccccn2)cc1	4,30	BCGset	
GSK1568930A	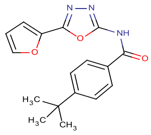	gskversion	CC(C)(C)c1ccc(cc1)C(=O)Nc1nnc(o1)-c1ccco1	4,30	BCGset	
GSK1752826A	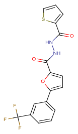	gskversion	FC(F)(F)c1cccc(c1)-c1ccc(o1)C(=O)NNC(=O)c1cccs1	4,30	BCGset	

GSK1826248A	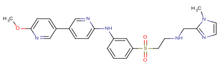	gskversion	COc1ccc(cn1)-c1ccc(Nc2cccc(c2)S(=O)(=O)CCNCc2nccn2C)nc1	4,30	BCGset	
GSK1839994A	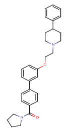	gskversion	O=C(N1CCCC1)c1ccc(cc1)-c1cccc(OCCN2CCC(CC2)c2ccccc2)c1	4,30	BCGset	
GSK391431A	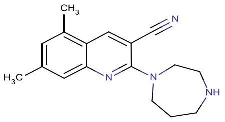	gskversion	Cc1cc(C)c2cc(C#N)c(nc2c1)N1CCCNCC1	4,30	BCGset	
GSK395715A	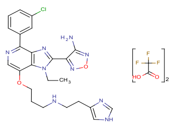	gskversion	CCn1c(nc2c(ncc(OCCCNCCc3c[nH]cn3)c12)-c1cccc(Cl)c1)-c1nonc1N	4,30	BCGset	
GSK430424A	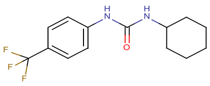	gskversion	FC(F)(F)c1ccc(NC(=O)NC2CCCCC2)cc1	4,30	BCGset	
GSK433913A	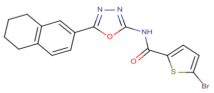	gskversion	Brc1ccc(s1)C(=O)Nc1nnc(o1)-c1ccc2CCCCc2c1	4,30	BCGset	
GSK1589671A	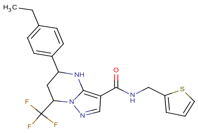	gskversion	CCc1ccc(cc1)C1CC(n2ncc(C(=O)NCc3cccs3)c2N1)C(F)(F)F	4,37	BCGset	TBset
GSK1017370A	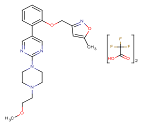	gskversion	COCCN1CCN(CC1)c1ncc(cn1)-c1ccccc1OCc1cc(C)on1	4,40	BCGset	
GSK1057434A	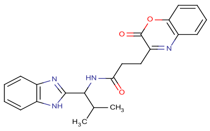	gskversion	CC(C)C(NC(=O)CCc1nc2ccccc2oc1=O)c1nc2ccccc2[nH]1	4,40	BCGset	
GSK1103973A	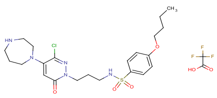	gskversion	CCCCOc1ccc(cc1)S(=O)(=O)NCCCn1nc(Cl)c(cc1=O)N1CCCNCC1	4,40	BCGset	
GSK1155282A	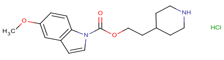	gskversion	COc1ccc2n(ccc2c1)C(=O)OCCC1CCNCC1	4,40	BCGset	
GSK1839971A	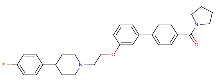	gskversion	Fc1ccc(cc1)C1CCN(CCOc2cccc(c2)-c2ccc(cc2)C(=O)N2CCCC2)CC1	4,40	BCGset	
GSK1926119A	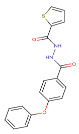	gskversion	O=C(NNC(=O)c1ccc(Oc2ccccc2)cc1)c1cccs1	4,40	BCGset	

GSK2197582A	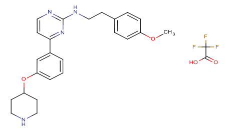	gskversion	COc1ccc(CCNc2nccc(n2)-c2cccc(OC3CCNCC3)c2)cc1	4,40	BCGset	
GSK260308A	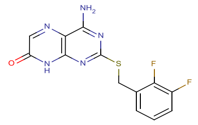	gskversion	Nc1nc(SCc2cccc(F)c2F)nc2[nH]c(=O)cnc12	4,40	BCGset	
GSK339049A	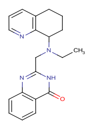	gskversion	CCN(Cc1nc2ccccc2c(=O)[nH]1)C1CCCc2cccnc12	4,40	BCGset	
GSK447517A	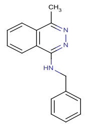	gskversion	Cc1nnc(NCc2ccccc2)c2ccccc12	4,40	BCGset	
GSK502518A	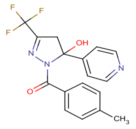	gskversion	Cc1ccc(cc1)C(=O)N1N=C(CC1(O)c1ccncc1)C(F)(F)F	4,40	BCGset	
GSK975812A	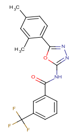	gskversion	Cc1ccc(-c2nnc(NC(=O)c3cccc(c3)C(F)(F)F)o2)c(C)c1	4,40	BCGset	
GSK976023A	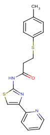	gskversion	Cc1ccc(SCCC(=O)Nc2nc(cs2)-c2ccccn2)cc1	4,40	BCGset	
GSK1761215A	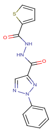	gskversion	O=C(NNC(=O)c1cccs1)c1cnn(n1)-c1ccccc1	4,50	BCGset	
GSK432630A	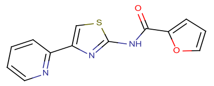	gskversion	O=C(Nc1nc(cs1)-c1ccccn1)c1ccco1	4,50	BCGset	
GSK500400A	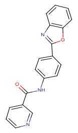	gskversion	O=C(Nc1ccc(cc1)-c1nc2ccccc2o1)c1cccnc1	4,50	BCGset	
GSK381407A	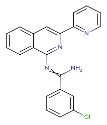	gskversion	NC(=Nc1nc(cc2ccccc12)-c1ccccn1)c1cccc(Cl)c1	4,50	BCGset	TBset
SB-712970	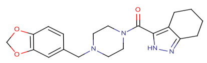	gskversion	O=C(N1CCN(Cc2ccc3OCOc3c2)CC1)c1[nH]nc2CCCCc12	4,50	BCGset	TBset
GSK1103976A	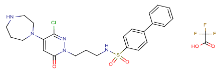	gskversion	Clc1nn(CCCNS(=O)(=O)c2ccc(cc2)-c2ccccc2)c(=O)cc1N1CCCNCC1	4,60	BCGset	

GSK1239234A	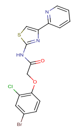	gskversion	Clc1cc(Br)ccc1OCC(=O)Nc1nc(cs1)-c1ccccn1	4,60	BCGset	
GSK1288009A	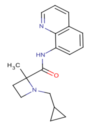	gskversion	CC1(CCN1CC1CC1)C(=O)Nc1cccc2cccnc12	4,60	BCGset	
GSK1293949A	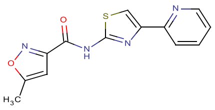	gskversion	Cc1cc(no1)C(=O)Nc1nc(cs1)-c1ccccn1	4,60	BCGset	
GSK1445244A	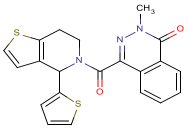	gskversion	Cn1nc(C(=O)N2CCc3sccc3C2c2cccs2)c2ccccc2c1=O	4,60	BCGset	
GSK516030A	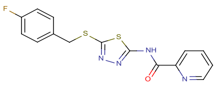	gskversion	Fc1ccc(CSc2nnc(NC(=O)c3ccccn3)s2)cc1	4,60	BCGset	
GSK868100A	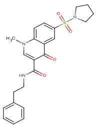	gskversion	Cn1cc(C(=O)NCCc2ccccc2)c(=O)c2cc(ccc12)S(=O)(=O)N1CCCC1	4,60	BCGset	
GW283926A	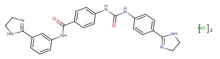	gskversion	O=C(Nc1ccc(cc1)C(=O)Nc1cccc(c1)C1=NCCN1)Nc1ccc(cc1)C1=NCCN1	4,60	BCGset	
SB-282992-A	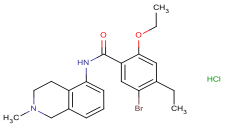	gskversion	CCOc1cc(CC)c(Br)cc1C(=O)Nc1cccc2CN(C)CCc12	4,60	BCGset	
GW713556X	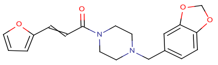	gskversion	O=C(C=Cc1ccco1)N1CCN(Cc2ccc3OCOc3c2)CC1	4,60	BCGset	TBset
SB-811137-V	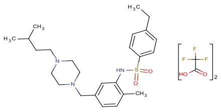	gskversion	CCc1ccc(cc1)S(=O)(=O)Nc1cc(CN2CCN(CCC(C)C)CC2)ccc1C	4,68	BCGset	TBset
GSK1826247A	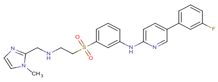	gskversion	Cn1ccnc1CNCCS(=O)(=O)c1cccc(Nc2ccc(cn2)-c2cccc(F)c2)c1	4,68	BCGset	TBset
GSK1584732A	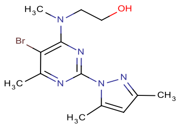	gskversion	CN(CCO)c1nc(nc(C)c1Br)-n1nc(C)cc1C	4,70	BCGset	
GSK1650100A	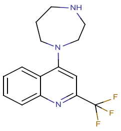	gskversion	FC(F)(F)c1cc(N2CCCNCC2)c2ccccc2n1	4,70	BCGset	

GSK1688273A	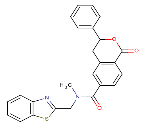	gskversion	CN(Cc1nc2ccccc2s1)C(=O)c1ccc2C(=O)OC(Cc2c1)c1ccccc1	4,70	BCGset	
GSK1804923A	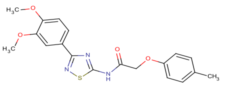	gskversion	COc1ccc(cc1OC)-c1nsc(NC(=O)COc2ccc(C)cc2)n1	4,70	BCGset	
GSK2200163A	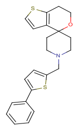	gskversion	C(N1CCC2(CC1)OCCc1sccc21)c1ccc(s1)-c1ccccc1	4,70	BCGset	
GSK281210A	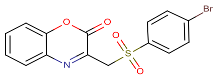	gskversion	Brc1ccc(cc1)S(=O)(=O)Cc1nc2ccccc2oc1=O	4,70	BCGset	
GSK574899A	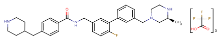	gskversion	C[C@H]1CN(Cc2cccc(c2)-c2cc(CNC(=O)c3ccc(CC4CCNCC4)cc3)ccc2F)CCN1	4,70	BCGset	
GSK920693A	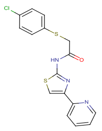	gskversion	Clc1ccc(SCC(=O)Nc2nc(cs2)-c2ccccn2)cc1	4,70	BCGset	
GSK754716A	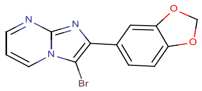	gskversion	Brc1c(nc2ncccn12)-c1ccc2OCOc2c1	4,70	BCGset	TBset
GSK705278A	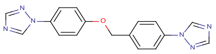	gskversion	C(Oc1ccc(cc1)-n1cncn1)c1ccc(cc1)-n1cncn1	4,73	BCGset	TBset
GSK1352957A	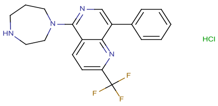	gskversion	FC(F)(F)c1ccc2c(ncc(-c3ccccc3)c2n1)N1CCCNCC1	4,80	BCGset	
GSK1460091A	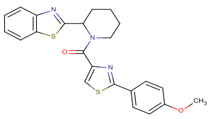	gskversion	COc1ccc(cc1)-c1nc(cs1)C(=O)N1CCCCC1c1nc2ccccc2s1	4,80	BCGset	
GSK1589678A	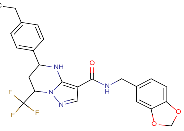	gskversion	CCc1ccc(cc1)C1CC(n2ncc(C(=O)NCc3ccc4OCOc4c3)c2N1)C(F)(F)F	4,80	BCGset	
GSK1740917A	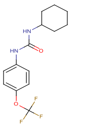	gskversion	FC(F)(F)Oc1ccc(NC(=O)NC2CCCCC2)cc1	4,80	BCGset	
GSK1783685A	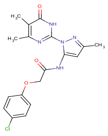	gskversion	Cc1cc(NC(=O)COc2ccc(Cl)cc2)n(n1)-c1nc(C)c(C)c(=O)[nH]1	4,80	BCGset	

GSK2118612A	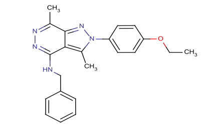	gskversion	CCOc1ccc(cc1)-n1nc2c(C)nnc(NCc3ccccc3)c2c1C	4,80	BCGset	
GSK426083A	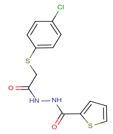	gskversion	Clc1ccc(SCC(=O)NNC(=O)c2cccs2)cc1	4,80	BCGset	
GSK1941290A	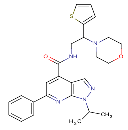	gskversion	CC(C)n1ncc2c(cc(nc12)-c1ccccc1)C(=O)NCC(N1CCOCC1)c1cccs1	4,80	BCGset	TBset
GSK1826825A	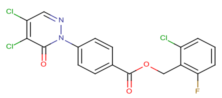	gskversion	Fc1cccc(Cl)c1COC(=O)c1ccc(cc1)-n1ncc(Cl)c(Cl)c1=O	4,90	BCGset	TBset
GSK490439A	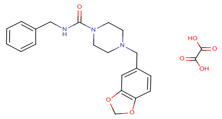	gskversion	O=C(NCc1ccccc1)N1CCN(Cc2ccc3OCOc3c2)CC1	4,90	BCGset	
GSK1589673A	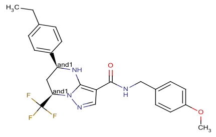	gskversion	CCc1ccc(cc1)C1CC(n2ncc(C(=O)NCc3ccc(OC)cc3)c2N1)C(F)(F)F	4,90	BCGset	TBset
BRL-18223AV	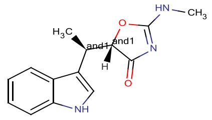	gskversion	CNC1=NC(=O)[C@@H](O1)[C@H](C)c1c[nH]c2ccccc12	5,00	BCGset	
GSK1215271A	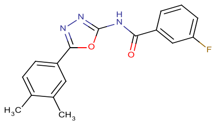	gskversion	Cc1ccc(cc1C)-c1nnc(NC(=O)c2cccc(F)c2)o1	5,00	BCGset	
GSK1257009A	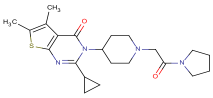	gskversion	Cc1sc2nc(C3CC3)n(C3CCN(CC(=O)N4CCCC4)CC3)c(=O)c2c1C	5,00	BCGset	
GSK1277123A	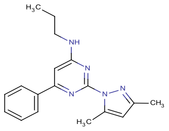	gskversion	CCCNc1cc(nc(n1)-n1nc(C)cc1C)-c1ccccc1	5,00	BCGset	
GSK900362A	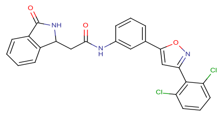	gskversion	Clc1cccc(Cl)c1-c1cc(on1)-c1cccc(NC(=O)CC2NC(=O)c3ccccc23)c1	5,00	BCGset	
GSK847920A	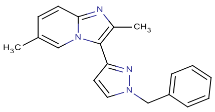	gskversion	Cc1nc2ccc(C)cn2c1-c1ccn(Cc2ccccc2)n1	5,00	BCGset	TBset
GSK1599440A	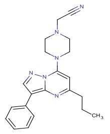	gskversion	CCCc1cc(N2CCN(CC#N)CC2)n2ncc(-c3ccccc3)c2n1	5,10	BCGset	

GSK1761037A	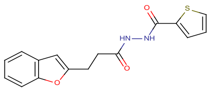	gskversion	O=C(CCc1cc2ccccc2o1)NNC(=O)c1cccs1	5,10	BCGset	
GSK626837B	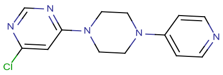	gskversion	Clc1cc(ncn1)N1CCN(CC1)c1ccncc1	5,10	BCGset	
GSK275628A	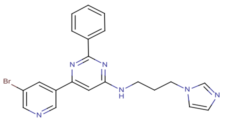	gskversion	Brc1cncc(c1)-c1cc(NCCCn2ccnc2)nc(n1)-c1ccccc1	5,19	BCGset	TBset
GSK1073593A	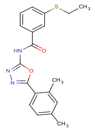	gskversion	CCSc1cccc(c1)C(=O)Nc1nnc(o1)-c1ccc(C)cc1C	5,20	BCGset	
GSK1073647A	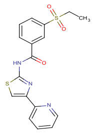	gskversion	CCS(=O)(=O)c1cccc(c1)C(=O)Nc1nc(cs1)-c1ccccn1	5,20	BCGset	
GSK1239841A	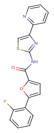	gskversion	Fc1ccccc1-c1ccc(o1)C(=O)Nc1nc(cs1)-c1ccccn1	5,20	BCGset	
GSK1615227A	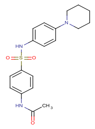	gskversion	CC(=O)Nc1ccc(cc1)S(=O)(=O)Nc1ccc(cc1)N1CCCCC1	5,20	BCGset	
GSK1746325A	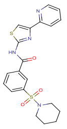	gskversion	O=C(Nc1nc(cs1)-c1ccccn1)c1cccc(c1)S(=O)(=O)N1CCCCC1	5,20	BCGset	
GW770961A	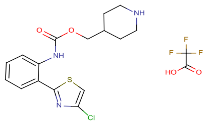	gskversion	Clc1csc(n1)-c1ccccc1NC(=O)OCC1CCNCC1	5,20	BCGset	
GSK636544A	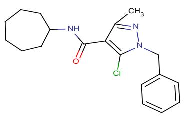	gskversion	Cc1nn(Cc2ccccc2)c(Cl)c1C(=O)NC1CCCCCC1	5,20	BCGset	TBset
GSK1434490A	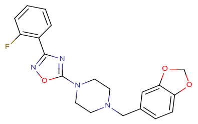	gskversion	Fc1ccccc1-c1noc(n1)N1CCN(Cc2ccc3OCOc3c2)CC1	5,20	BCGset	TBset
GSK1658592A	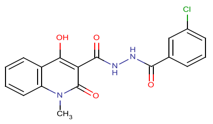	gskversion	Cn1c2ccccc2c(O)c(C(=O)NNC(=O)c2cccc(Cl)c2)c1=O	5,30	BCGset	
GSK1783684A	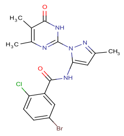	gskversion	Cc1cc(NC(=O)c2cc(Br)ccc2Cl)n(n1)-c1nc(C)c(C)c(=O)[nH]1	5,30	BCGset	

GSK1826011A	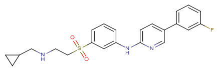	gskversion	Fc1cccc(c1)-c1ccc(Nc2cccc(c2)S(=O)(=O)CCNCC2CC2)nc1	5,30	BCGset	
GSK1982996A	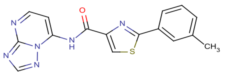	gskversion	Cc1cccc(c1)-c1nc(cs1)C(=O)Nc1ccnc2ncnn12	5,30	BCGset	
GSK350706A	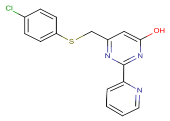	gskversion	Oc1cc(CSc2ccc(Cl)cc2)nc(n1)-c1ccccn1	5,30	BCGset	
GSK480736A	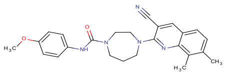	gskversion	COc1ccc(NC(=O)N2CCCN(CC2)c2nc3c(C)c(C)ccc3cc2C#N)cc1	5,30	BCGset	
GSK547499A	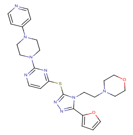	gskversion	C(Cn1c(Sc2ccnc(n2)N2CCN(CC2)c2ccncc2)nnc1-c1ccco1)N1CCOCC1	5,30	BCGset	
SB-563819	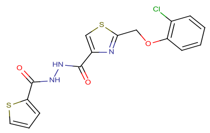	gskversion	Clc1ccccc1OCc1nc(cs1)C(=O)NNC(=O)c1cccs1	5,30	BCGset	
GSK345724A	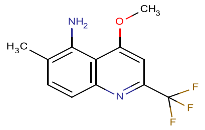	gskversion	COc1cc(nc2ccc(C)c(N)c12)C(F)(F)F	5,30	BCGset	TBset
GSK1731114A	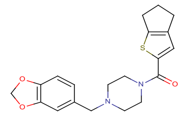	gskversion	O=C(N1CCN(Cc2ccc3OCOc3c2)CC1)c1cc2CCCc2s1	5,30	BCGset	TBset
BRL-51093AM	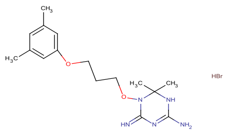	gskversion	Cc1cc(C)cc(OCCCON2C(=N)N=C(N)NC2(C)C)c1	5,31	BCGset	TBset
GSK1594233A	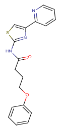	gskversion	O=C(CCCOc1ccccc1)Nc1nc(cs1)-c1ccccn1	5,40	BCGset	
GW645859X	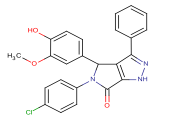	gskversion	COc1cc(ccc1O)C1N(C(=O)c2[nH]nc(c12)-c1ccccc1)c1ccc(Cl)cc1	5,40	BCGset	
GSK937733A	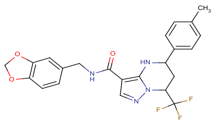	gskversion	Cc1ccc(cc1)C1CC(n2ncc(C(=O)NCc3ccc4OCOc4c3)c2N1)C(F)(F)F	5,40	BCGset	TBset
GSK1402290A	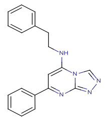	gskversion	C(Cc1ccccc1)Nc1cc(nc2nncn12)-c1ccccc1	5,50	BCGset	TBset

GSK1197849A	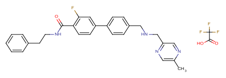	gskversion	Cc1cnc(CNCc2ccc(cc2)-c2ccc(C(=O)NCCc3ccccc3)c(F)c2)cn1	5,50	BCGset	
GSK120985A	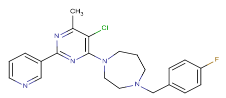	gskversion	Cc1nc(nc(N2CCCN(Cc3ccc(F)cc3)CC2)c1Cl)-c1cccnc1	5,50	BCGset	
GSK1274122A	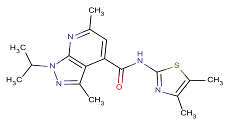	gskversion	CC(C)n1nc(C)c2c(cc(C)nc12)C(=O)Nc1nc(C)c(C)s1	5,50	BCGset	
GSK1299919A	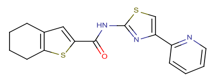	gskversion	O=C(Nc1nc(cs1)-c1ccccn1)c1cc2CCCCc2s1	5,50	BCGset	
GSK1836675A	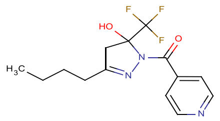	gskversion	CCCCC1=NN(C(=O)c2ccncc2)C(O)(C1)C(F)(F)F	5,50	BCGset	
GSK217635A	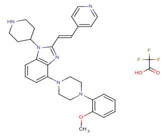	gskversion	COc1ccccc1N1CCN(CC1)c1cccc2n(C3CCNCC3)c(\C=C\c3ccncc3)nc12	5,50	BCGset	
GSK471625A	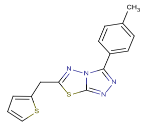	gskversion	Cc1ccc(cc1)-c1nnc2sc(Cc3cccs3)nn12	5,50	BCGset	
GSK918708A	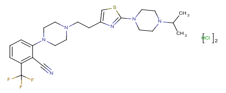	gskversion	CC(C)N1CCN(CC1)c1nc(CCN2CCN(CC2)c2cccc(c2C#N)C(F)(F)F)cs1	5,50	BCGset	
GW324595A	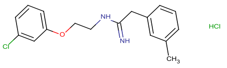	gskversion	Cc1cccc(CC(=N)NCCOc2cccc(Cl)c2)c1	5,50	BCGset	
SB-252962-A	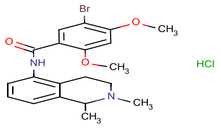	gskversion	COc1cc(OC)c(cc1Br)C(=O)Nc1cccc2C(C)N(C)CCc12	5,50	BCGset	
GSK1588120A	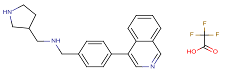	gskversion	C(NCc1ccc(cc1)-c1cncc2ccccc12)C1CCNC1	5,56	BCGset	TBset
GSK1188075A	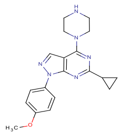	gskversion	COc1ccc(cc1)-n1ncc2c(nc(nc12)C1CC1)N1CCNCC1	5,60	BCGset	
GSK1443033A	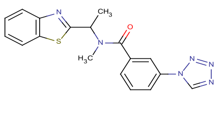	gskversion	CC(N(C)C(=O)c1cccc(c1)-n1cnnn1)c1nc2ccccc2s1	5,60	BCGset	

GSK1788523A	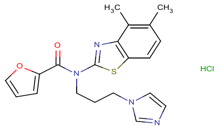	gskversion	Cc1ccc2sc(nc2c1C)N(CCCn1ccnc1)C(=O)c1ccco1	5,60	BCGset	
GSK1826012A	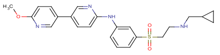	gskversion	COc1ccc(cn1)-c1ccc(Nc2cccc(c2)S(=O)(=O)CCNCC2CC2)nc1	5,60	BCGset	
GSK1924991A	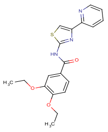	gskversion	CCOc1ccc(cc1OCC)C(=O)Nc1nc(cs1)-c1ccccn1	5,60	BCGset	
GSK1999915A	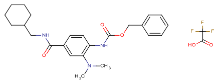	gskversion	CN(C)c1cc(ccc1NC(=O)OCc1ccccc1)C(=O)NCC1CCCCC1	5,60	BCGset	
GSK714667A	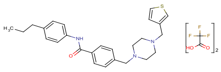	gskversion	CCCc1ccc(NC(=O)c2ccc(CN3CCN(Cc4ccsc4)CC3)cc2)cc1	5,60	BCGset	
SB-252961-A	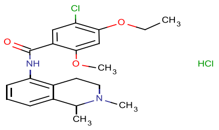	gskversion	CCOc1cc(OC)c(cc1Cl)C(=O)Nc1cccc2C(C)N(C)CCc12	5,60	BCGset	
GSK130506A	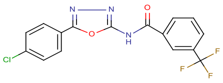	gskversion	FC(F)(F)c1cccc(c1)C(=O)Nc1nnc(o1)-c1ccc(Cl)cc1	5,62	BCGset	TBset
GSK1925843A	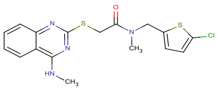	gskversion	CNc1nc(SCC(=O)N(C)Cc2ccc(Cl)s2)nc2ccccc12	5,62	BCGset	TBset
GSK1210019A	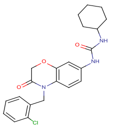	gskversion	Clc1ccccc1CN1C(=O)COc2cc(NC(=O)NC3CCCCC3)ccc12	5,70	BCGset	
GSK1256173A	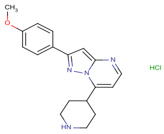	gskversion	COc1ccc(cc1)-c1cc2nccc(C3CCNCC3)n2n1	5,70	BCGset	
GSK1679883A	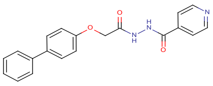	gskversion	O=C(COc1ccc(cc1)-c1ccccc1)NNC(=O)c1ccncc1	5,70	BCGset	
GSK1825940A	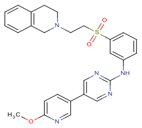	gskversion	COc1ccc(cn1)-c1cnc(Nc2cccc(c2)S(=O)(=O)CCN2CCc3ccccc3C2)nc1	5,70	BCGset	
GSK1881038A	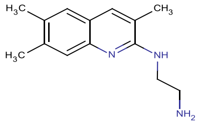	gskversion	Cc1cc2cc(C)c(NCCN)nc2cc1C	5,70	BCGset	

GW658979X	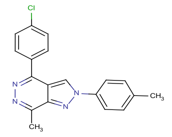	gskversion	Cc1ccc(cc1)-n1cc2c(nnc(C)c2n1)-c1ccc(Cl)cc1	5,70	BCGset	
GSK107678A	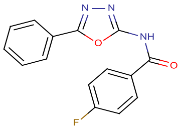	gskversion	Fc1ccc(cc1)C(=O)Nc1nnc(o1)-c1ccccc1	5,80	BCGset	
SB-443369	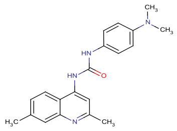	gskversion	CN(C)c1ccc(NC(=O)Nc2cc(C)nc3cc(C)ccc23)cc1	5,80	BCGset	
GSK991960A	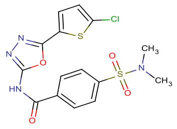	gskversion	CN(C)S(=O)(=O)c1ccc(cc1)C(=O)Nc1nnc(o1)-c1ccc(Cl)s1	5,82	BCGset	TBset
GSK1505361A	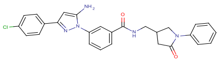	gskversion	Nc1cc(nn1-c1cccc(c1)C(=O)NCC1CN(C(=O)C1)c1ccccc1)-c1ccc(Cl)cc1	5,90	BCGset	
GSK2148633A	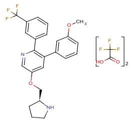	gskversion	COc1cccc(c1)-c1cc(OC[C@@H]2CCCN2)cnc1-c1cccc(c1)C(F)(F)F	5,90	BCGset	
GSK497430A	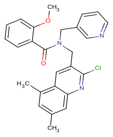	gskversion	COc1ccccc1C(=O)N(Cc1cccnc1)Cc1cc2c(C)cc(C)cc2nc1Cl	5,90	BCGset	
GSK607186A	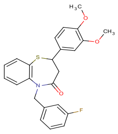	gskversion	COc1ccc(cc1OC)C1CC(=O)N(Cc2cccc(F)c2)c2ccccc2S1	5,90	BCGset	
GSK669378A	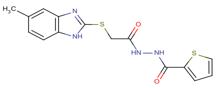	gskversion	Cc1ccc2[nH]c(SCC(=O)NNC(=O)c3cccs3)nc2c1	5,90	BCGset	
GSK986289A	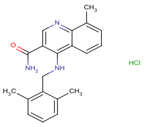	gskversion	Cc1cccc(C)c1CNc1c(cnc2c(C)cccc12)C(N)=O	5,90	BCGset	
SB-253290-A	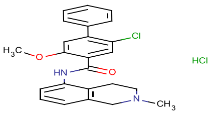	gskversion	COc1cc(c(Cl)cc1C(=O)Nc1cccc2CN(C)CCc12)-c1ccccc1	5,90	BCGset	
SB-729215-A	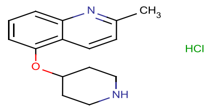	gskversion	Cc1ccc2c(OC3CCNCC3)cccc2n1	5,90	BCGset	
GSK445886A	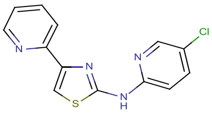	gskversion	Clc1ccc(Nc2nc(cs2)-c2ccccn2)nc1	5,90	BCGset	TBset

GSK1302651A	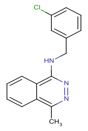	gskversion	Cc1nnc(NCc2cccc(Cl)c2)c2ccccc12	5,90	BCGset	TBset
GSK107516A	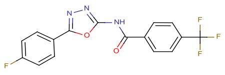	gskversion	Fc1ccc(cc1)-c1nnc(NC(=O)c2ccc(cc2)C(F)(F)F)o1	6,00	BCGset	
GSK1254879A	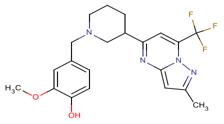	gskversion	COc1cc(CN2CCCC(C2)c2cc(n3nc(C)cc3n2)C(F)(F)F)ccc1O	6,00	BCGset	
GSK1722405A	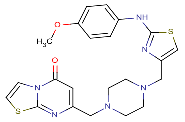	gskversion	COc1ccc(Nc2nc(CN3CCN(Cc4cc(=O)n5ccsc5n4)CC3)cs2)cc1	6,00	BCGset	
GSK1815207A	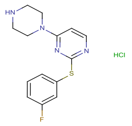	gskversion	Fc1cccc(Sc2nccc(n2)N2CCNCC2)c1	6,00	BCGset	
GSK1825998A	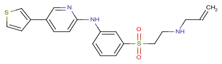	gskversion	C=CCNCCS(=O)(=O)c1cccc(Nc2ccc(cn2)-c2ccsc2)c1	6,00	BCGset	
GSK2168465A	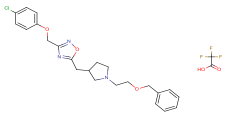	gskversion	Clc1ccc(OCc2noc(CC3CCN(CCOCc4ccccc4)C3)n2)cc1	6,00	BCGset	
GSK272286A	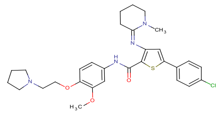	gskversion	COc1cc(NC(=O)c2sc(cc2\N=C2\CCCCN2C)-c2ccc(Cl)cc2)ccc1OCCN1CCCC1	6,00	BCGset	
GW819798X	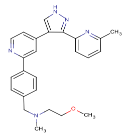	gskversion	COCCN(C)Cc1ccc(cc1)-c1cc(ccn1)-c1c[nH]nc1-c1cccc(C)n1	6,00	BCGset	
GSK937213A	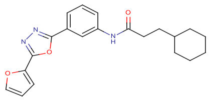	gskversion	O=C(CCC1CCCCC1)Nc1cccc(c1)-c1nnc(o1)-c1ccco1	6,10	BCGset	TBset
GSK1301131A	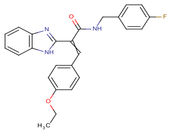	gskversion	CCOc1ccc(C=C(C(=O)NCc2ccc(F)cc2)c2nc3ccccc3[nH]2)cc1	6,10	BCGset	
GSK1519000A	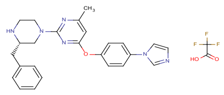	gskversion	Cc1cc(Oc2ccc(cc2)-n2ccnc2)nc(n1)N1CCN[C@@H](Cc2ccccc2)C1	6,10	BCGset	
GSK1664204A	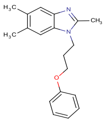	gskversion	Cc1nc2cc(C)c(C)cc2n1CCCOc1ccccc1	6,10	BCGset	

GSK1717306A	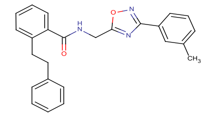	gskversion	Cc1cccc(c1)-c1noc(CNC(=O)c2ccccc2CCc2ccccc2)n1	6,10	BCGset	
GSK431728A	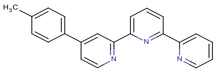	gskversion	Cc1ccc(cc1)-c1ccnc(c1)-c1cccc(n1)-c1ccccn1	6,10	BCGset	
GSK809140A	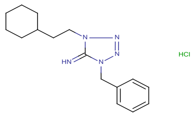	gskversion	N=c1n(CCC2CCCCC2)nnn1Cc1ccccc1	6,10	BCGset	
GSK817289A	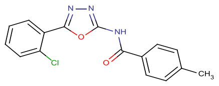	gskversion	Cc1ccc(cc1)C(=O)Nc1nnc(o1)-c1ccccc1Cl	6,10	BCGset	
GW357070A	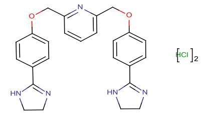	gskversion	C(Oc1ccc(cc1)C1=NCCN1)c1cccc(COc2ccc(cc2)C2=NCCN2)n1	6,10	BCGset	
GSK270670A	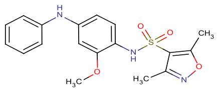	gskversion	COc1cc(Nc2ccccc2)ccc1NS(=O)(=O)c1c(C)noc1C	6,10	BCGset	TBset
GI247341A	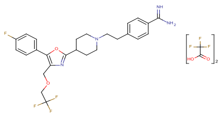	gskversion	NC(=N)c1ccc(CCN2CCC(CC2)c2nc(COCC(F)(F)F)c(o2)-c2ccc(F)cc2)cc1	6,17	BCGset	TBset
GSK1045380A	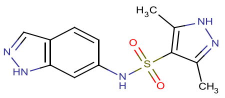	gskversion	Cc1n[nH]c(C)c1S(=O)(=O)Nc1ccc2cn[nH]c2c1	6,20	BCGset	
GSK1191667A	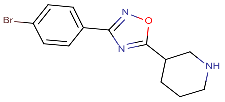	gskversion	Brc1ccc(cc1)-c1noc(n1)C1CCCNC1	6,20	BCGset	
GSK1573961A	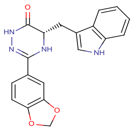	gskversion	O=C1NN=C(N[C@H]1Cc1c[nH]c2ccccc12)c1ccc2OCOc2c1	6,20	BCGset	
GSK1615454A	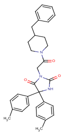	gskversion	Cc1ccc(cc1)C1(NC(=O)N(CC(=O)N2CCC(Cc3ccccc3)CC2)C1=O)c1ccc(C)cc1	6,20	BCGset	
GSK1783660A	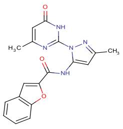	gskversion	Cc1cc(NC(=O)c2cc3ccccc3o2)n(n1)-c1nc(C)cc(=O)[nH]1	6,20	BCGset	
GSK1920455A	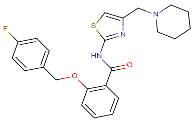	gskversion	Fc1ccc(COc2ccccc2C(=O)Nc2nc(CN3CCCCC3)cs2)cc1	6,20	BCGset	

GSK686665A	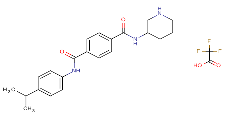	gskversion	CC(C)c1ccc(NC(=O)c2ccc(cc2)C(=O)NC2CCCNC2)cc1	6,20	BCGset	
GSK997258A	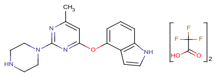	gskversion	Cc1cc(Oc2cccc3[nH]ccc23)nc(n1)N1CCNCC1	6,20	BCGset	
GSK1829660A	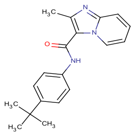	gskversion	Cc1nc2ccccn2c1C(=O)Nc1ccc(cc1)C(C)(C)C	6,20	BCGset	TBset
GSK1480644A	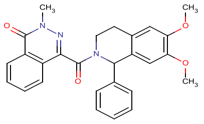	gskversion	COc1cc2CCN(C(c3ccccc3)c2cc1OC)C(=O)c1nn(C)c(=O)c2ccccc12	6,30	BCGset	
GSK1576706A	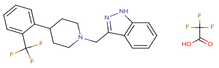	gskversion	FC(F)(F)c1ccccc1C1CCN(Cc2n[nH]c3ccccc23)CC1	6,30	BCGset	
GSK1583389A	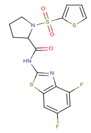	gskversion	Fc1cc(F)c2nc(NC(=O)C3CCCN3S(=O)(=O)c3cccs3)sc2c1	6,30	BCGset	
GSK547491A	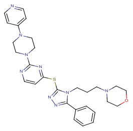	gskversion	C(CN1CCOCC1)Cn1c(Sc2ccnc(n2)N2CCN(CC2)c2ccncc2)nnc1-c1ccccc1	6,30	BCGset	
GSK547525A	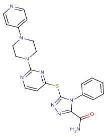	gskversion	NC(=O)c1nnc(Sc2ccnc(n2)N2CCN(CC2)c2ccncc2)n1-c1ccccc1	6,30	BCGset	
GW609615X	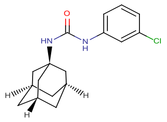	gskversion	Clc1cccc(NC(=O)NC23CC4CC(CC(C4)C2)C3)c1	6,30	BCGset	
GSK547543A	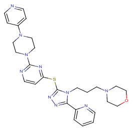	gskversion	C(CN1CCOCC1)Cn1c(Sc2ccnc(n2)N2CCN(CC2)c2ccncc2)nnc1-c1ccccn1	6,38	BCGset	TBset
GSK1054388A	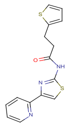	gskversion	O=C(CCc1cccs1)Nc1nc(cs1)-c1ccccn1	6,40	BCGset	
GSK1279771A	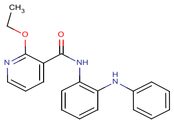	gskversion	CCOc1ncccc1C(=O)Nc1ccccc1Nc1ccccc1	6,40	BCGset	
GSK1307955A	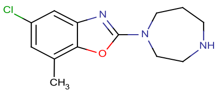	gskversion	Cc1cc(Cl)cc2nc(oc12)N1CCCNCC1	6,40	BCGset	

GSK1581732A	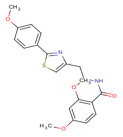	gskversion	COc1ccc(cc1)-c1nc(CCNC(=O)c2ccc(OC)cc2OC)cs1	6,40	BCGset	
GSK1583248A	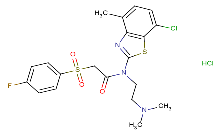	gskversion	CN(C)CCN(C(=O)CS(=O)(=O)c1ccc(F)cc1)c1nc2c(C)ccc(Cl)c2s1	6,40	BCGset	
GSK1688709A	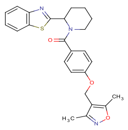	gskversion	Cc1noc(C)c1COc1ccc(cc1)C(=O)N1CCCCC1c1nc2ccccc2s1	6,40	BCGset	
GSK1825341A	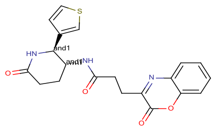	gskversion	O=C(CCc1nc2ccccc2oc1=O)N[C@@H]1CCC(=O)N[C@H]1c1ccsc1	6,40	BCGset	
GSK2200145A	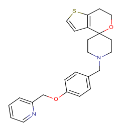	gskversion	C(Oc1ccc(CN2CCC3(CC2)OCCc2sccc32)cc1)c1ccccn1	6,40	BCGset	
GSK459972A	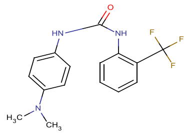	gskversion	CN(C)c1ccc(NC(=O)Nc2ccccc2C(F)(F)F)cc1	6,40	BCGset	
SB-251011-A	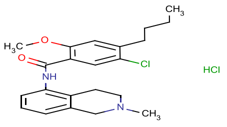	gskversion	CCCCc1cc(OC)c(cc1Cl)C(=O)Nc1cccc2CN(C)CCc12	6,40	BCGset	
SB-430458	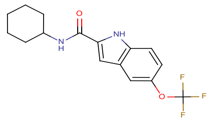	gskversion	FC(F)(F)Oc1ccc2[nH]c(cc2c1)C(=O)NC1CCCCC1	6,40	BCGset	
GI103688B	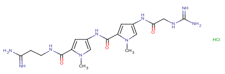	gskversion	Cn1cc(NC(=O)c2cc(NC(=O)CNC(N)=N)cn2C)cc1C(=O)NCCC(N)=N	6,46	BCGset	TBset
GSK1256136A	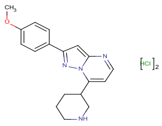	gskversion	COc1ccc(cc1)-c1cc2nccc(C3CCCNC3)n2n1	6,50	BCGset	
GSK156448A	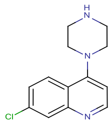	gskversion	Clc1ccc2c(ccnc2c1)N1CCNCC1	6,50	BCGset	
GSK1839898A	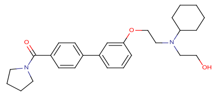	gskversion	OCCN(CCOc1cccc(c1)-c1ccc(cc1)C(=O)N1CCCC1)C1CCCCC1	6,50	BCGset	
GSK431563A	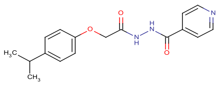	gskversion	CC(C)c1ccc(OCC(=O)NNC(=O)c2ccncc2)cc1	6,50	BCGset	

SB-713533	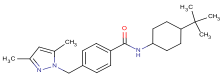	gskversion	Cc1cc(C)n(Cc2ccc(cc2)C(=O)NC2CCC(CC2)C(C)(C)C)n1	6,50	BCGset	
GSK786331A	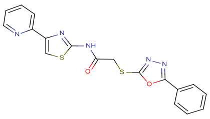	gskversion	O=C(CSc1nnc(o1)-c1ccccc1)Nc1nc(cs1)-c1ccccn1	6,60	BCGset	
GSK1758774A	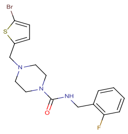	gskversion	Fc1ccccc1CNC(=O)N1CCN(Cc2ccc(Br)s2)CC1	6,60	BCGset	TBset
GSK1729177A	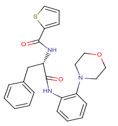	gskversion	O=C(Nc1ccccc1N1CCOCC1)[C@H](Cc1ccccc1)NC(=O)c1cccs1	6,60	BCGset	TBset
GSK1650514A	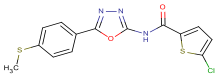	gskversion	CSc1ccc(cc1)-c1nnc(NC(=O)c2ccc(Cl)s2)o1	6,61	BCGset	TBset
GSK1812410A	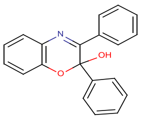	gskversion	OC1(Oc2ccccc2N=C1c1ccccc1)c1ccccc1	6,68	BCGset	TBset
GSK1147407A	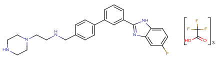	gskversion	Fc1ccc2[nH]c(nc2c1)-c1cccc(c1)-c1ccc(CNCCN2CCNCC2)cc1	6,70	BCGset	
GSK1272261A	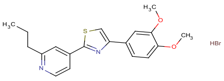	gskversion	CCCc1cc(ccn1)-c1nc(cs1)-c1ccc(OC)c(OC)c1	6,70	BCGset	
GSK1278038A	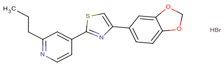	gskversion	CCCc1cc(ccn1)-c1nc(cs1)-c1ccc2OCOc2c1	6,70	BCGset	
GSK160672A	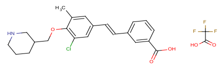	gskversion	Cc1cc(\C=C\c2cccc(c2)C(O)=O)cc(Cl)c1OCC1CCCNC1	6,70	BCGset	
GSK2124696A	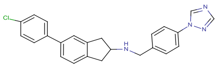	gskversion	Clc1ccc(cc1)-c1ccc2CC(Cc2c1)NCc1ccc(cc1)-n1cncn1	6,70	BCGset	
GSK2170062A	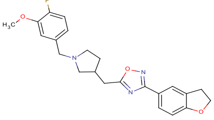	gskversion	COc1cc(CN2CCC(Cc3nc(no3)-c3ccc4OCCc4c3)C2)ccc1F	6,70	BCGset	
GSK380205A	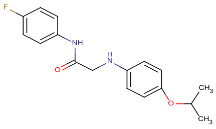	gskversion	CC(C)Oc1ccc(NCC(=O)Nc2ccc(F)cc2)cc1	6,70	BCGset	

GSK825286A	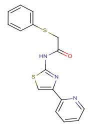	gskversion	O=C(CSc1ccccc1)Nc1nc(cs1)-c1ccccn1	6,70	BCGset	
GW874674A	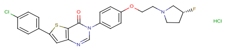	gskversion	F[C@@H]1CCN(CCOc2ccc(cc2)-n2cnc3cc(sc3c2=O)-c2ccc(Cl)cc2)C1	6,70	BCGset	
GSK831784A	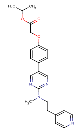	gskversion	CC(C)OC(=O)COc1ccc(cc1)-c1cnc(nc1)N(C)CCc1ccncc1	6,76	BCGset	TBset
GSK152499A	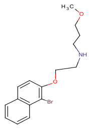	gskversion	COCCCNCCOc1ccc2ccccc2c1Br	6,80	BCGset	
GSK391272A	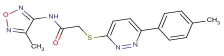	gskversion	Cc1nonc1NC(=O)CSc1ccc(nn1)-c1ccc(C)cc1	6,80	BCGset	
GSK409004A	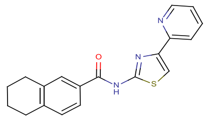	gskversion	O=C(Nc1nc(cs1)-c1ccccn1)c1ccc2CCCCc2c1	6,80	BCGset	
GSK854602A	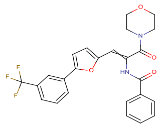	gskversion	FC(F)(F)c1cccc(c1)-c1ccc(C=C(NC(=O)c2ccccc2)C(=O)N2CCOCC2)o1	6,80	BCGset	
GW362225X	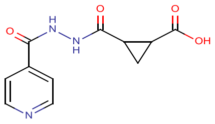	gskversion	OC(=O)C1CC1C(=O)NNC(=O)c1ccncc1	6,80	BCGset	
SB-274894-A	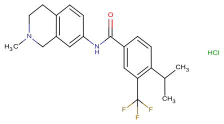	gskversion	CC(C)c1ccc(cc1C(F)(F)F)C(=O)Nc1ccc2CCN(C)Cc2c1	6,80	BCGset	
GSK1857145A	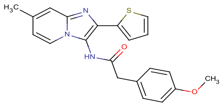	gskversion	COc1ccc(CC(=O)Nc2c(nc3cc(C)ccn23)-c2cccs2)cc1	6,80	BCGset	TBset
GSK1214891A	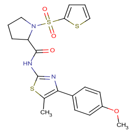	gskversion	COc1ccc(cc1)-c1nc(NC(=O)C2CCCN2S(=O)(=O)c2cccs2)sc1C	6,90	BCGset	
GSK1307488A	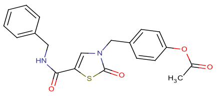	gskversion	CC(=O)Oc1ccc(Cn2cc(sc2=O)C(=O)NCc2ccccc2)cc1	6,90	BCGset	
GSK1402087A	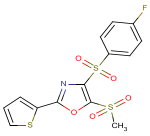	gskversion	CS(=O)(=O)c1oc(nc1S(=O)(=O)c1ccc(F)cc1)-c1cccs1	6,90	BCGset	

GSK1623433A	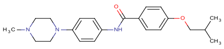	gskversion	CC(C)COc1ccc(cc1)C(=O)Nc1ccc(cc1)N1CCN(C)CC1	6,90	BCGset	
GSK1722666A	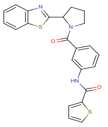	gskversion	O=C(Nc1cccc(c1)C(=O)N1CCCC1c1nc2ccccc2s1)c1cccs1	6,90	BCGset	
GSK1783644A	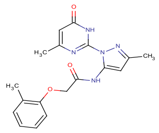	gskversion	Cc1cc(NC(=O)COc2ccccc2C)n(n1)-c1nc(C)cc(=O)[nH]1	6,90	BCGset	
GSK1826255A	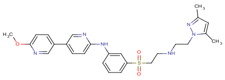	gskversion	COc1ccc(cn1)-c1ccc(Nc2cccc(c2)S(=O)(=O)CCNCCn2nc(C)cc2C)nc1	6,90	BCGset	
GSK391898A	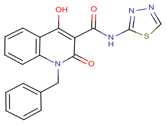	gskversion	Oc1c(C(=O)Nc2nncs2)c(=O)n(Cc2ccccc2)c2ccccc12	6,90	BCGset	
GSK486838A	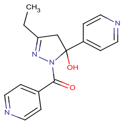	gskversion	CCC1=NN(C(=O)c2ccncc2)C(O)(C1)c1ccncc1	6,90	BCGset	
GSK547527A	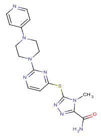	gskversion	Cn1c(Sc2ccnc(n2)N2CCN(CC2)c2ccncc2)nnc1C(N)=O	6,90	BCGset	
GSK753238A	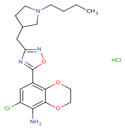	gskversion	CCCCN1CCC(Cc2noc(n2)-c2cc(Cl)c(N)c3OCCOc23)C1	6,90	BCGset	
GSK817310A	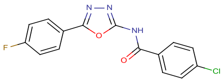	gskversion	Fc1ccc(cc1)-c1nnc(NC(=O)c2ccc(Cl)cc2)o1	6,90	BCGset	
GW507275X	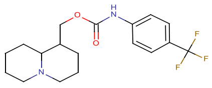	gskversion	FC(F)(F)c1ccc(NC(=O)OCC2CCCN3CCCCC23)cc1	6,90	BCGset	
GSK1035931A	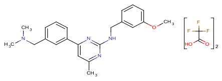	gskversion	COc1cccc(CNc2nc(C)cc(n2)-c2cccc(CN(C)C)c2)c1	7,00	BCGset	
GSK1093557A	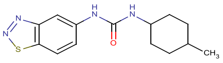	gskversion	CC1CCC(CC1)NC(=O)Nc1ccc2snnc2c1	7,00	BCGset	
GSK1565002A	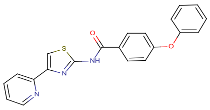	gskversion	O=C(Nc1nc(cs1)-c1ccccn1)c1ccc(Oc2ccccc2)cc1	7,00	BCGset	

GSK1567135A	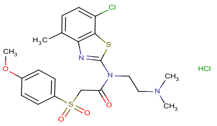	gskversion	COc1ccc(cc1)S(=O)(=O)CC(=O)N(CCN(C)C)c1nc2c(C)ccc(Cl)c2s1	7,00	BCGset	
GSK1570383A	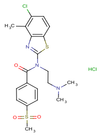	gskversion	CN(C)CCN(C(=O)c1ccc(cc1)S(C)(=O)=O)c1nc2c(C)c(Cl)ccc2s1	7,00	BCGset	
GSK1825829A	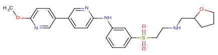	gskversion	COc1ccc(cn1)-c1ccc(Nc2cccc(c2)S(=O)(=O)CCNCC2CCCO2)nc1	7,00	BCGset	
GSK1911186A	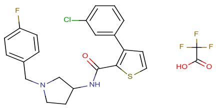	gskversion	Fc1ccc(CN2CCC(C2)NC(=O)c2sccc2-c2cccc(Cl)c2)cc1	7,00	BCGset	
GSK202782A	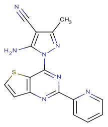	gskversion	Cc1nn(c(N)c1C#N)-c1nc(nc2ccsc12)-c1ccccn1	7,00	BCGset	
GSK512203A	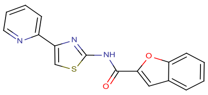	gskversion	O=C(Nc1nc(cs1)-c1ccccn1)c1cc2ccccc2o1	7,00	BCGset	
GSK971223A	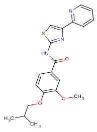	gskversion	COc1cc(ccc1OCC(C)C)C(=O)Nc1nc(cs1)-c1ccccn1	7,00	BCGset	
GSK976468A	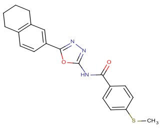	gskversion	CSc1ccc(cc1)C(=O)Nc1nnc(o1)-c1ccc2CCCCc2c1	7,00	BCGset	
SB-640828-V	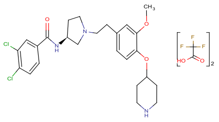	gskversion	COc1cc(CCN2CC[C@@H](C2)NC(=O)c2ccc(Cl)c(Cl)c2)ccc1OC1CCNCC1	7,00	BCGset	
SB-830656	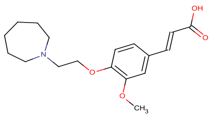	gskversion	COc1cc(\C=C\C(O)=O)ccc1OCCN1CCCCCC1	7,00	BCGset	
GSK1051703A	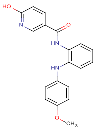	gskversion	COc1ccc(Nc2ccccc2NC(=O)c2ccc(O)nc2)cc1	7,08	BCGset	TBset
GSK848336A	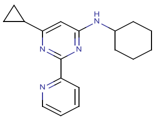	gskversion	C1CC1c1cc(NC2CCCCC2)nc(n1)-c1ccccn1	7,08	BCGset	TBset
GSK1090537A	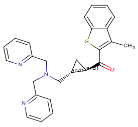	gskversion	Cc1c(sc2ccccc12)C(=O)[C@@H]1C[C@H]1CN(Cc1ccccn1)Cc1ccccn1	7,10	BCGset	

GSK1427115A	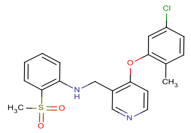	gskversion	Cc1ccc(Cl)cc1Oc1ccncc1CNc1ccccc1S(C)(=O)=O	7,10	BCGset	
GSK298771A	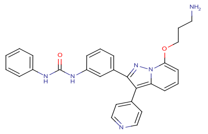	gskversion	NCCCOc1cccc2c(c(nn12)-c1cccc(NC(=O)Nc2ccccc2)c1)-c1ccncc1	7,10	BCGset	
SB-254878	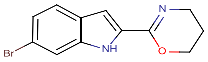	gskversion	Brc1ccc2cc([nH]c2c1)C1=NCCCO1	7,10	BCGset	
SB-457709-BT	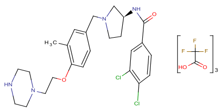	gskversion	Cc1cc(CN2CC[C@@H](C2)NC(=O)c2ccc(Cl)c(Cl)c2)ccc1OCCN1CCNCC1	7,10	BCGset	
GSK1073395A	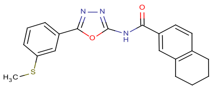	gskversion	CSc1cccc(c1)-c1nnc(NC(=O)c2ccc3CCCCc3c2)o1	7,20	BCGset	
GSK2105756A	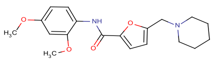	gskversion	COc1ccc(NC(=O)c2ccc(CN3CCCCC3)o2)c(OC)c1	7,20	BCGset	
GSK497029A	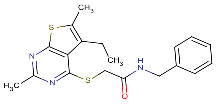	gskversion	CCc1c(C)sc2nc(C)nc(SCC(=O)NCc3ccccc3)c12	7,20	BCGset	
GSK874867A	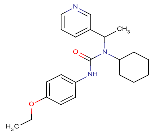	gskversion	CCOc1ccc(NC(=O)N(C(C)c2cccnc2)C2CCCCC2)cc1	7,20	BCGset	
GSK1254220A	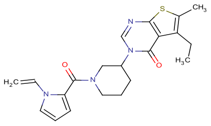	gskversion	CCc1c(C)sc2ncn(C3CCCN(C3)C(=O)c3cccn3C=C)c(=O)c12	7,30	BCGset	
GSK1826033A	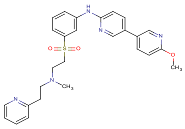	gskversion	COc1ccc(cn1)-c1ccc(Nc2cccc(c2)S(=O)(=O)CCN(C)CCc2ccccn2)nc1	7,30	BCGset	
GSK1911140A	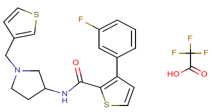	gskversion	Fc1cccc(c1)-c1ccsc1C(=O)NC1CCN(Cc2ccsc2)C1	7,30	BCGset	
GSK2016627A	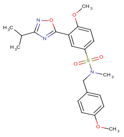	gskversion	COc1ccc(CN(C)S(=O)(=O)c2ccc(OC)c(c2)-c2nc(no2)C(C)C)cc1	7,30	BCGset	
GSK735506A	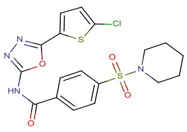	gskversion	Clc1ccc(s1)-c1nnc(NC(=O)c2ccc(cc2)S(=O)(=O)N2CCCCC2)o1	7,30	BCGset	

SB-205641-A	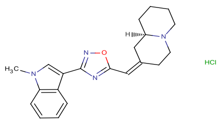	gskversion	Cn1cc(-c2noc(\C=C3\CCN4CCCC[C@@H]4C3)n2)c2ccccc12	7,30	BCGset	
SB-330946-A	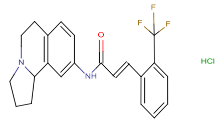	gskversion	FC(F)(F)c1ccccc1\C=C\C(=O)Nc1ccc2CCN3CCCC3c2c1	7,30	BCGset	
GSK1734508A	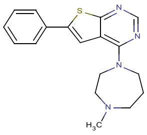	gskversion	CN1CCCN(CC1)c1ncnc2sc(cc12)-c1ccccc1	7,40	BCGset	
GSK1825921A	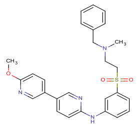	gskversion	COc1ccc(cn1)-c1ccc(Nc2cccc(c2)S(=O)(=O)CCN(C)Cc2ccccc2)nc1	7,40	BCGset	
GSK437009A	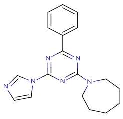	gskversion	C1CCCN(CC1)c1nc(nc(n1)-n1ccnc1)-c1ccccc1	7,41	BCGset	TBset
GSK276001A	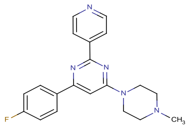	gskversion	CN1CCN(CC1)c1cc(nc(n1)-c1ccncc1)-c1ccc(F)cc1	7,41	BCGset	TBset
GSK1097740A	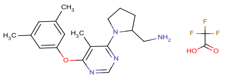	gskversion	Cc1cc(C)cc(Oc2ncnc(N3CCCC3CN)c2C)c1	7,50	BCGset	
GSK147297A	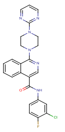	gskversion	Fc1ccc(NC(=O)c2cnc(N3CCN(CC3)c3ncccn3)c3ccccc23)cc1Cl	7,50	BCGset	
GSK1783678A	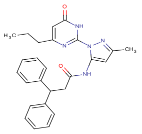	gskversion	CCCc1cc(=O)[nH]c(n1)-n1nc(C)cc1NC(=O)CC(c1ccccc1)c1ccccc1	7,50	BCGset	
GSK1839228A	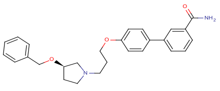	gskversion	NC(=O)c1cccc(c1)-c1ccc(OCCCN2CC[C@H](C2)OCc2ccccc2)cc1	7,50	BCGset	
GSK233136A	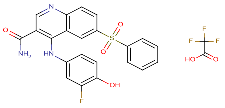	gskversion	NC(=O)c1cnc2ccc(cc2c1Nc1ccc(O)c(F)c1)S(=O)(=O)c1ccccc1	7,50	BCGset	
GSK380055A	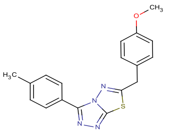	gskversion	COc1ccc(Cc2nn3c(nnc3s2)-c2ccc(C)cc2)cc1	7,50	BCGset	
GSK471629A	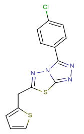	gskversion	Clc1ccc(cc1)-c1nnc2sc(Cc3cccs3)nn12	7,50	BCGset	

GSK621231A	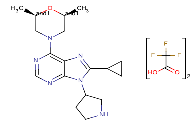	gskversion	C[C@H]1CN(C[C@@H](C)O1)c1ncnc2n(C3CCNC3)c(nc12)C1CC1	7,50	BCGset	
GSK997261A	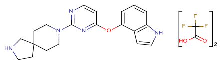	gskversion	C1CC2(CN1)CCN(CC2)c1nccc(Oc2cccc3[nH]ccc23)n1	7,50	BCGset	
GSK1826089A	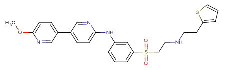	gskversion	COc1ccc(cn1)-c1ccc(Nc2cccc(c2)S(=O)(=O)CCNCCc2cccs2)nc1	7,59	BCGset	TBset
GSK479031A	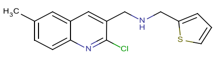	gskversion	Cc1ccc2nc(Cl)c(CNCc3cccs3)cc2c1	7,59	BCGset	TBset
GSK1274062A	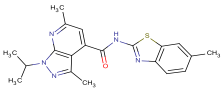	gskversion	CC(C)n1nc(C)c2c(cc(C)nc12)C(=O)Nc1nc2ccc(C)cc2s1	7,60	BCGset	
GSK1752553A	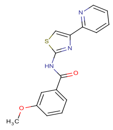	gskversion	COc1cccc(c1)C(=O)Nc1nc(cs1)-c1ccccn1	7,60	BCGset	
GSK1967906A	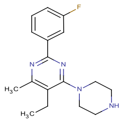	gskversion	CCc1c(C)nc(nc1N1CCNCC1)-c1cccc(F)c1	7,60	BCGset	
GSK316415A	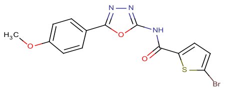	gskversion	COc1ccc(cc1)-c1nnc(NC(=O)c2ccc(Br)s2)o1	7,60	BCGset	
GSK727404A	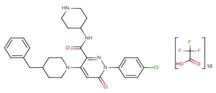	gskversion	Clc1ccc(cc1)-n1nc(C(=O)NC2CCNCC2)c(cc1=O)N1CCC(Cc2ccccc2)CC1	7,60	BCGset	
GW478077X	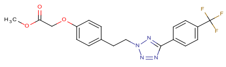	gskversion	COC(=O)COc1ccc(CCn2nnc(n2)-c2ccc(cc2)C(F)(F)F)cc1	7,60	BCGset	
SB-790652-M	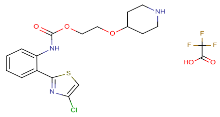	gskversion	Clc1csc(n1)-c1ccccc1NC(=O)OCCOC1CCNCC1	7,60	BCGset	
GSK1055950A	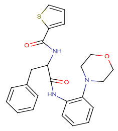	gskversion	O=C(Nc1ccccc1N1CCOCC1)C(Cc1ccccc1)NC(=O)c1cccs1	7,60	BCGset	TBset
GSK1829816A	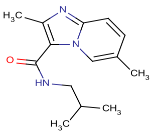	gskversion	CC(C)CNC(=O)c1c(C)nc2ccc(C)cn12	7,67	BCGset	TBset

GSK1905227A	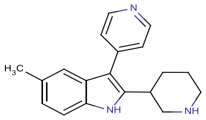	gskversion	Cc1ccc2[nH]c(C3CCCNC3)c(-c3ccncc3)c2c1	7,67	BCGset	TBset
GSK1855672A	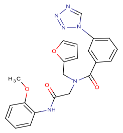	gskversion	COc1ccccc1NC(=O)CN(Cc1ccco1)C(=O)c1cccc(c1)-n1cnnn1	7,70	BCGset	
GSK933302A	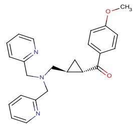	gskversion	COc1ccc(cc1)C(=O)[C@H]1C[C@@H]1CN(Cc1ccccn1)Cc1ccccn1	7,70	BCGset	
SB-657505	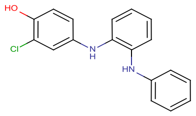	gskversion	Oc1ccc(Nc2ccccc2Nc2ccccc2)cc1Cl	7,70	BCGset	
SB-811796-V	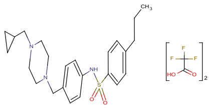	gskversion	CCCc1ccc(cc1)S(=O)(=O)Nc1ccc(CN2CCN(CC3CC3)CC2)cc1	7,76	BCGset	TBset
GSK1863309A	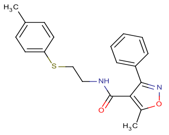	gskversion	Cc1onc(c1C(=O)NCCSc1ccc(C)cc1)-c1ccccc1	7,76	BCGset	TBset
GSK316438A	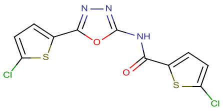	gskversion	Clc1ccc(s1)C(=O)Nc1nnc(o1)-c1ccc(Cl)s1	7,76	BCGset	TBset
GSK1107705A	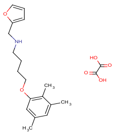	gskversion	Cc1cc(C)c(C)c(OCCCCNCc2ccco2)c1	7,80	BCGset	
GSK1166774A	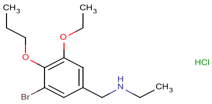	gskversion	CCCOc1c(Br)cc(CNCC)cc1OCC	7,80	BCGset	
GSK1839982A	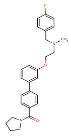	gskversion	CN(CCOc1cccc(c1)-c1ccc(cc1)C(=O)N1CCCC1)Cc1ccc(F)cc1	7,80	BCGset	
GSK900872A	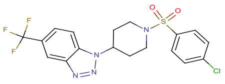	gskversion	FC(F)(F)c1ccc2n(nnc2c1)C1CCN(CC1)S(=O)(=O)c1ccc(Cl)cc1	7,80	BCGset	
GW362236X	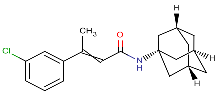	gskversion	CC(=CC(=O)NC12CC3CC(CC(C3)C1)C2)c1cccc(Cl)c1	7,80	BCGset	
GSK547511A	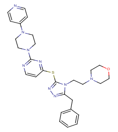	gskversion	C(Cn1c(Cc2ccccc2)nnc1Sc1ccnc(n1)N1CCN(CC1)c1ccncc1)N1CCOCC1	7,85	BCGset	TBset

SB-706404	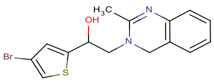	gskversion	CC1=Nc2ccccc2CN1CC(O)c1cc(Br)cs1	7,85	BCGset	TBset
GSK1103979A	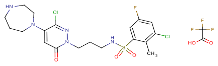	gskversion	Cc1c(Cl)cc(F)cc1S(=O)(=O)NCCCn1nc(Cl)c(cc1=O)N1CCCNCC1	7,90	BCGset	
GSK1826297A	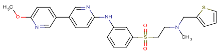	gskversion	COc1ccc(cn1)-c1ccc(Nc2cccc(c2)S(=O)(=O)CCN(C)Cc2cccs2)nc1	7,90	BCGset	
GSK431107A	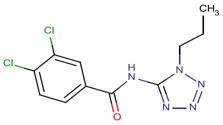	gskversion	CCCn1nnnc1NC(=O)c1ccc(Cl)c(Cl)c1	7,90	BCGset	
GSK541718A	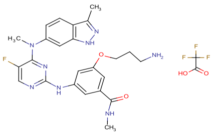	gskversion	CNC(=O)c1cc(Nc2ncc(F)c(n2)N(C)c2ccc3c(C)n[nH]c3c2)cc(OCCCN)c1	7,90	BCGset	
GW496492X	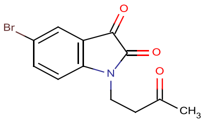	gskversion	CC(=O)CCN1C(=O)C(=O)c2cc(Br)ccc12	7,90	BCGset	
GW560770X	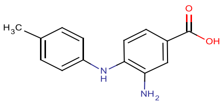	gskversion	Cc1ccc(Nc2ccc(cc2N)C(O)=O)cc1	7,90	BCGset	
GSK1691553A	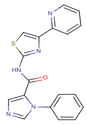	gskversion	O=C(Nc1nc(cs1)-c1ccccn1)c1cncn1-c1ccccc1	7,94	BCGset	TBset
GSK146660A	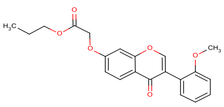	gskversion	CCCOC(=O)COc1ccc2c(c1)occ(-c1ccccc1OC)c2=O	7,94	BCGset	TBset
GSK1150241A	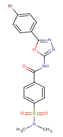	gskversion	CN(C)S(=O)(=O)c1ccc(cc1)C(=O)Nc1nnc(o1)-c1ccc(Br)cc1	8,00	BCGset	
GSK1460068A	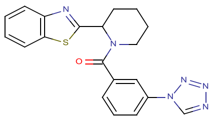	gskversion	O=C(N1CCCCC1c1nc2ccccc2s1)c1cccc(c1)-n1cnnn1	8,00	BCGset	
GW726795A	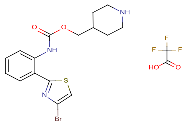	gskversion	Brc1csc(n1)-c1ccccc1NC(=O)OCC1CCNCC1	8,00	BCGset	
SB-830300	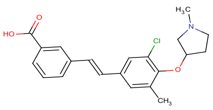	gskversion	CN1CCC(C1)Oc1c(C)cc(\C=C\c2cccc(c2)C(O)=O)cc1Cl	8,00	BCGset	

GSK468214A	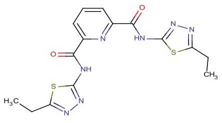	gskversion	CCc1nnc(NC(=O)c2cccc(n2)C(=O)Nc2nnc(CC)s2)s1	8,00	BCGset	TBset
GSK1281439A	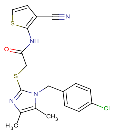	gskversion	Cc1nc(SCC(=O)Nc2sccc2C#N)n(Cc2ccc(Cl)cc2)c1C	8,10	BCGset	
GSK1586679A	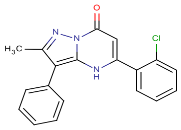	gskversion	Cc1nn2c([nH]c(cc2=O)-c2ccccc2Cl)c1-c1ccccc1	8,10	BCGset	
GSK1636456A	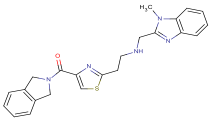	gskversion	Cn1c(CNCCc2nc(cs2)C(=O)N2Cc3ccccc3C2)nc2ccccc12	8,10	BCGset	
GSK380027A	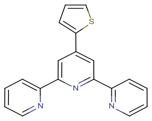	gskversion	c1csc(c1)-c1cc(nc(c1)-c1ccccn1)-c1ccccn1	8,10	BCGset	
GSK430471A	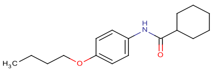	gskversion	CCCCOc1ccc(NC(=O)C2CCCCC2)cc1	8,10	BCGset	
GSK460708A	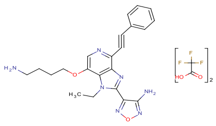	gskversion	CCn1c(nc2c(ncc(OCCCCN)c12)C#Cc1ccccc1)-c1nonc1N	8,10	BCGset	
GSK676357A	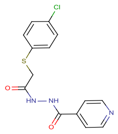	gskversion	Clc1ccc(SCC(=O)NNC(=O)c2ccncc2)cc1	8,10	BCGset	
SB-414272	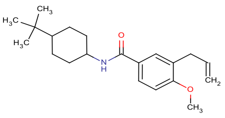	gskversion	COc1ccc(cc1CC=C)C(=O)NC1CCC(CC1)C(C)(C)C	8,10	BCGset	
GSK1733953A	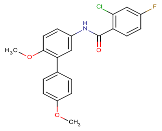	gskversion	COc1ccc(cc1)-c1cc(NC(=O)c2ccc(F)cc2Cl)ccc1OC	8,13	BCGset	TBset
GR124560X	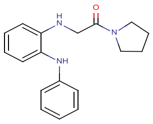	gskversion	O=C(CNc1ccccc1Nc1ccccc1)N1CCCC1	8,20	BCGset	
GSK1788506A	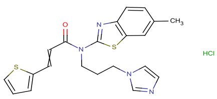	gskversion	Cc1ccc2nc(sc2c1)N(CCCn1ccnc1)C(=O)C=Cc1cccs1	8,20	BCGset	
GSK670323A	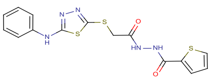	gskversion	O=C(CSc1nnc(Nc2ccccc2)s1)NNC(=O)c1cccs1	8,20	BCGset	

GSK878236A	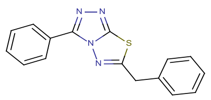	gskversion	C(c1nn2c(nnc2s1)-c1ccccc1)c1ccccc1	8,20	BCGset	
GSK690382A	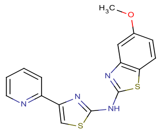	gskversion	COc1ccc2sc(Nc3nc(cs3)-c3ccccn3)nc2c1	8,20	BCGset	TBset
SB-552112	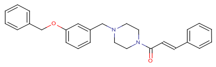	gskversion	O=C(\C=C\c1ccccc1)N1CCN(Cc2cccc(OCc3ccccc3)c2)CC1	8,22	BCGset	TBset
GSK126454A	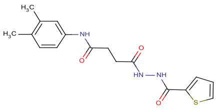	gskversion	Cc1ccc(NC(=O)CCC(=O)NNC(=O)c2cccs2)cc1C	8,30	BCGset	
GSK1752790A	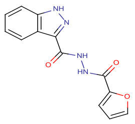	gskversion	O=C(NNC(=O)c1n[nH]c2ccccc12)c1ccco1	8,30	BCGset	
GSK880009A	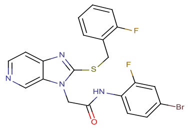	gskversion	Fc1ccccc1CSc1nc2ccncc2n1CC(=O)Nc1ccc(Br)cc1F	8,30	BCGset	
SB-701112-R	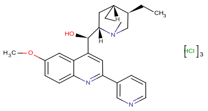	gskversion	CC[C@H]1CN2CC[C@@H]1C[C@H]2[C@H](O)c1cc(nc2ccc(OC)cc12)-c1cccnc1	8,30	BCGset	
GSK347301A	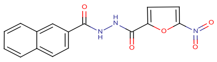	gskversion	O=C(NNC(=O)c1ccc2ccccc2c1)c1ccc(o1)N(=O)=O	8,32	BCGset	TBset
GSK1832831A	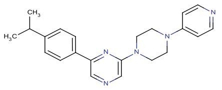	gskversion	CC(C)c1ccc(cc1)-c1cncc(n1)N1CCN(CC1)c1ccncc1	8,32	BCGset	TBset
GSK2157753A	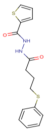	gskversion	O=C(CCCSc1ccccc1)NNC(=O)c1cccs1	8,32	BCGset	TBset
GSK1578152A	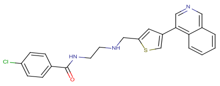	gskversion	Clc1ccc(cc1)C(=O)NCCNCc1cc(cs1)-c1cncc2ccccc12	8,40	BCGset	
GSK552672A	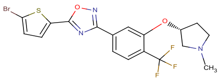	gskversion	CN1CC[C@H](C1)Oc1cc(ccc1C(F)(F)F)-c1noc(n1)-c1ccc(Br)s1	8,40	BCGset	
SB-715964-H	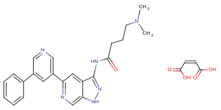	gskversion	CN(C)CCCC(=O)Nc1n[nH]c2cnc(cc12)-c1cncc(c1)-c1ccccc1	8,40	BCGset	

GSK1385423A	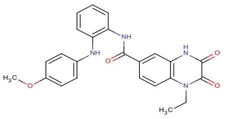	gskversion	CCn1c2ccc(cc2[nH]c(=O)c1=O)C(=O)Nc1ccccc1Nc1ccc(OC)cc1	8,40	BCGset	TBset
GSK1372568A	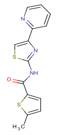	gskversion	Cc1ccc(s1)C(=O)Nc1nc(cs1)-c1ccccn1	8,41	BCGset	TBset
GSK1788487A	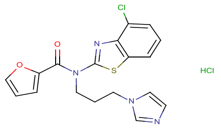	gskversion	Clc1cccc2sc(nc12)N(CCCn1ccnc1)C(=O)c1ccco1	8,41	BCGset	TBset
GSK920703A	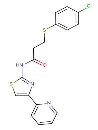	gskversion	Clc1ccc(SCCC(=O)Nc2nc(cs2)-c2ccccn2)cc1	8,41	BCGset	TBset
GSK1079318A	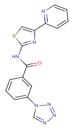	gskversion	O=C(Nc1nc(cs1)-c1ccccn1)c1cccc(c1)-n1cnnn1	8,50	BCGset	
GSK1132084A	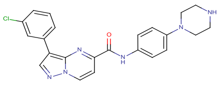	gskversion	Clc1cccc(c1)-c1cnn2ccc(nc12)C(=O)Nc1ccc(cc1)N1CCNCC1	8,50	BCGset	
GSK1406589A	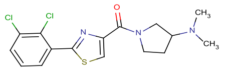	gskversion	CN(C)C1CCN(C1)C(=O)c1csc(n1)-c1cccc(Cl)c1Cl	8,50	BCGset	
GSK1556382A	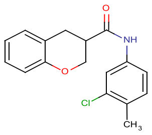	gskversion	Cc1ccc(NC(=O)C2COc3ccccc3C2)cc1Cl	8,50	BCGset	
GSK1770209A	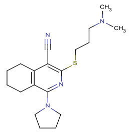	gskversion	CN(C)CCCSc1nc(N2CCCC2)c2CCCCc2c1C#N	8,50	BCGset	
GSK746360A	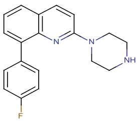	gskversion	Fc1ccc(cc1)-c1cccc2ccc(nc12)N1CCNCC1	8,50	BCGset	
SB-577234	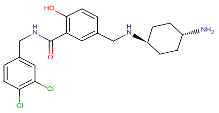	gskversion	N[C@H]1CC[C@@H](CC1)NCc1ccc(O)c(c1)C(=O)NCc1ccc(Cl)c(Cl)c1	8,50	BCGset	
GSK1170100A	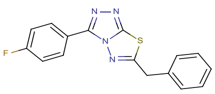	gskversion	Fc1ccc(cc1)-c1nnc2sc(Cc3ccccc3)nn12	8,60	BCGset	
GSK1863941A	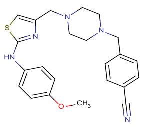	gskversion	COc1ccc(Nc2nc(CN3CCN(Cc4ccc(cc4)C#N)CC3)cs2)cc1	8,60	BCGset	

GSK1956503A	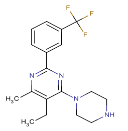	gskversion	CCc1c(C)nc(nc1N1CCNCC1)-c1cccc(c1)C(F)(F)F	8,60	BCGset	
SB-579760	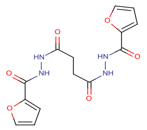	gskversion	O=C(CCC(=O)NNC(=O)c1ccco1)NNC(=O)c1ccco1	8,60	BCGset	
GSK1107112A	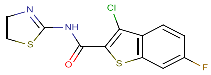	gskversion	Fc1ccc2c(Cl)c(sc2c1)C(=O)NC1=NCCS1	8,61	BCGset	TBset
GSK1598164A	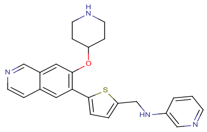	gskversion	C(Nc1cccnc1)c1ccc(s1)-c1cc2ccncc2cc1OC1CCNCC1	8,61	BCGset	TBset
GR118224A	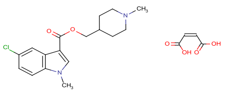	gskversion	CN1CCC(COC(=O)c2cn(C)c3ccc(Cl)cc23)CC1	8,70	BCGset	
GSK1484742A	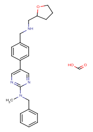	gskversion	CN(Cc1ccccc1)c1ncc(cn1)-c1ccc(CNCC2CCCO2)cc1	8,70	BCGset	
GSK512438A	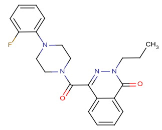	gskversion	CCCn1nc(C(=O)N2CCN(CC2)c2ccccc2F)c2ccccc2c1=O	8,70	BCGset	
GSK920694A	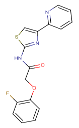	gskversion	Fc1ccccc1OCC(=O)Nc1nc(cs1)-c1ccccn1	8,70	BCGset	
GSK921295A	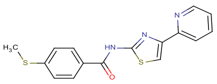	gskversion	CSc1ccc(cc1)C(=O)Nc1nc(cs1)-c1ccccn1	8,71	BCGset	TBset
GW664700A	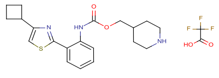	gskversion	O=C(Nc1ccccc1-c1nc(cs1)C1CCC1)OCC1CCNCC1	8,71	BCGset	TBset
GW369335X	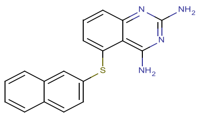	gskversion	Nc1nc(N)c2c(Sc3ccc4ccccc4c3)cccc2n1	8,71	BCGset	TBset
GSK1459976A	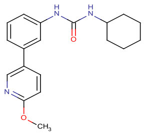	gskversion	COc1ccc(cn1)-c1cccc(NC(=O)NC2CCCCC2)c1	8,80	BCGset	
GSK1537821A	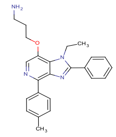	gskversion	CCn1c(nc2c(ncc(OCCCN)c12)-c1ccc(C)cc1)-c1ccccc1	8,80	BCGset	

GSK1559212A	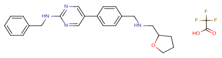	gskversion	C(NCc1ccc(cc1)-c1cnc(NCc2ccccc2)nc1)C1CCCO1	8,80	BCGset	
GSK2093583A	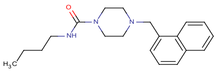	gskversion	CCCCNC(=O)N1CCN(Cc2cccc3ccccc23)CC1	8,80	BCGset	
GSK2201491A	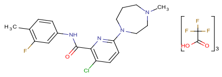	gskversion	CN1CCCN(CC1)c1ccc(Cl)c(n1)C(=O)Nc1ccc(C)c(F)c1	8,80	BCGset	
GSK357733A	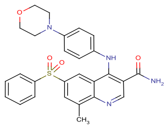	gskversion	Cc1cc(cc2c(Nc3ccc(cc3)N3CCOCC3)c(cnc12)C(N)=O)S(=O)(=O)c1ccccc1	8,80	BCGset	
GSK362325A	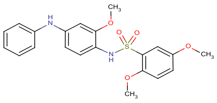	gskversion	COc1ccc(OC)c(c1)S(=O)(=O)Nc1ccc(Nc2ccccc2)cc1OC	8,80	BCGset	
GSK498384A	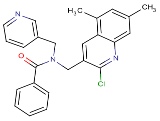	gskversion	Cc1cc(C)c2cc(CN(Cc3cccnc3)C(=O)c3ccccc3)c(Cl)nc2c1	8,80	BCGset	
GSK522834A	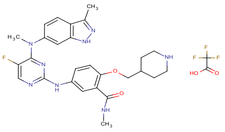	gskversion	CNC(=O)c1cc(Nc2ncc(F)c(n2)N(C)c2ccc3c(C)n[nH]c3c2)ccc1OCC1CCNCC1	8,80	BCGset	
GW663013X	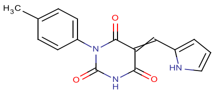	gskversion	Cc1ccc(cc1)N1C(=O)NC(=O)C(=Cc2ccc[nH]2)C1=O	8,80	BCGset	
GSK889423A	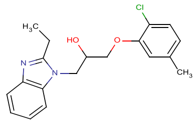	gskversion	CCc1nc2ccccc2n1CC(O)COc1cc(C)ccc1Cl	8,81	BCGset	TBset
GSK1066536A	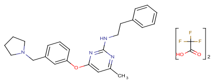	gskversion	Cc1cc(Oc2cccc(CN3CCCC3)c2)nc(NCCc2ccccc2)n1	8,90	BCGset	
GSK1374356A	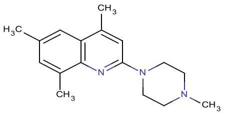	gskversion	CN1CCN(CC1)c1cc(C)c2cc(C)cc(C)c2n1	8,90	BCGset	
GSK2044743A	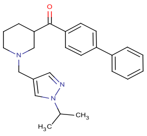	gskversion	CC(C)n1cc(CN2CCCC(C2)C(=O)c2ccc(cc2)-c2ccccc2)cn1	8,90	BCGset	
GSK561950A	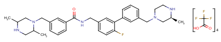	gskversion	C[C@H]1CN(Cc2cccc(c2)-c2cc(CNC(=O)c3cccc(CN4CC(C)NCC4C)c3)ccc2F)CCN1	8,90	BCGset	

GSK975784A	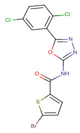	gskversion	Clc1ccc(Cl)c(c1)-c1nnc(NC(=O)c2ccc(Br)s2)o1	8,90	BCGset	
GSK1668869A	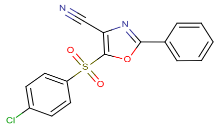	gskversion	Clc1ccc(cc1)S(=O)(=O)c1oc(nc1C#N)-c1ccccc1	8,90	BCGset	TBset
GSK547487A	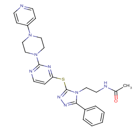	gskversion	CC(=O)NCCn1c(Sc2ccnc(n2)N2CCN(CC2)c2ccncc2)nnc1-c1ccccc1	8,91	BCGset	TBset
GSK1056042A	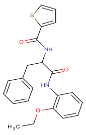	gskversion	CCOc1ccccc1NC(=O)C(Cc1ccccc1)NC(=O)c1cccs1	9,00	BCGset	
GSK1905762A	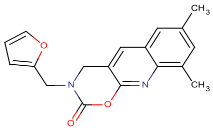	gskversion	Cc1cc(C)c2nc3OC(=O)N(Cc4ccco4)Cc3cc2c1	9,00	BCGset	
GSK1911142A	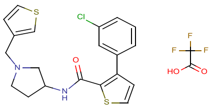	gskversion	Clc1cccc(c1)-c1ccsc1C(=O)NC1CCN(Cc2ccsc2)C1	9,00	BCGset	
GSK892651A	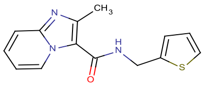	gskversion	Cc1nc2ccccn2c1C(=O)NCc1cccs1	9,02	BCGset	TBset
GSK275984A	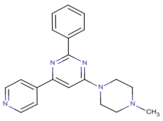	gskversion	CN1CCN(CC1)c1cc(nc(n1)-c1ccccc1)-c1ccncc1	9,02	BCGset	TBset
GSK1567329A	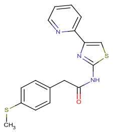	gskversion	CSc1ccc(CC(=O)Nc2nc(cs2)-c2ccccn2)cc1	9,10	BCGset	
GSK1774736A	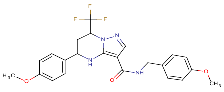	gskversion	COc1ccc(CNC(=O)c2cnn3C(CC(Nc23)c2ccc(OC)cc2)C(F)(F)F)cc1	9,10	BCGset	
GSK1859937A	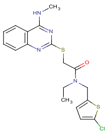	gskversion	CCN(Cc1ccc(Cl)s1)C(=O)CSc1nc(NC)c2ccccc2n1	9,10	BCGset	
GSK2200156A	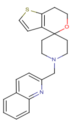	gskversion	C(N1CCC2(CC1)OCCc1sccc21)c1ccc2ccccc2n1	9,10	BCGset	
GW578349X	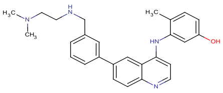	gskversion	CN(C)CCNCc1cccc(c1)-c1ccc2nccc(Nc3cc(O)ccc3C)c2c1	9,10	BCGset	

GSK352635A	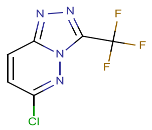	gskversion	FC(F)(F)c1nnc2ccc(Cl)nn12	9,10	BCGset	TBset
GSK1637967A	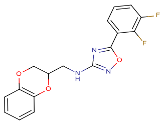	gskversion	Fc1cccc(-c2nc(NCC3COc4ccccc4O3)no2)c1F	9,20	BCGset	
GSK350834A	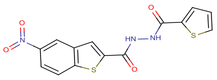	gskversion	O=C(NNC(=O)c1cc2cc(ccc2s1)N(=O)=O)c1cccs1	9,20	BCGset	
SB-388225-A	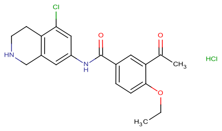	gskversion	CCOc1ccc(cc1C(C)=O)C(=O)Nc1cc(Cl)c2CCNCc2c1	9,20	BCGset	
GSK1518999A	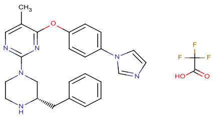	gskversion	Cc1cnc(nc1Oc1ccc(cc1)-n1ccnc1)N1CCN[C@@H](Cc2ccccc2)C1	9,23	BCGset	TBset
SB-746177	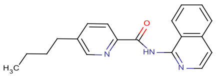	gskversion	CCCCc1ccc(nc1)C(=O)Nc1nccc2ccccc12	9,23	BCGset	TBset
GSK1117127A	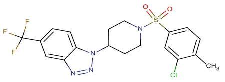	gskversion	Cc1ccc(cc1Cl)S(=O)(=O)N1CCC(CC1)n1nnc2cc(ccc12)C(F)(F)F	9,30	BCGset	
GSK2250079A	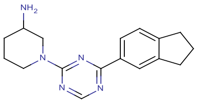	gskversion	NC1CCCN(C1)c1ncnc(n1)-c1ccc2CCCc2c1	9,30	BCGset	
GSK472569A	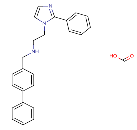	gskversion	C(Cn1ccnc1-c1ccccc1)NCc1ccc(cc1)-c1ccccc1	9,30	BCGset	
GW678927A	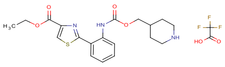	gskversion	CCOC(=O)c1csc(n1)-c1ccccc1NC(=O)OCC1CCNCC1	9,30	BCGset	
SB-204827-A	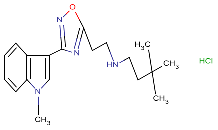	gskversion	Cn1cc(-c2noc(CCNCCC(C)(C)C)n2)c2ccccc12	9,30	BCGset	
GSK1570606A	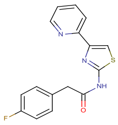	gskversion	Fc1ccc(CC(=O)Nc2nc(cs2)-c2ccccn2)cc1	9,30	BCGset	TBset
GSK1073410A	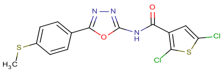	gskversion	CSc1ccc(cc1)-c1nnc(NC(=O)c2cc(Cl)sc2Cl)o1	9,40	BCGset	

GSK1286863A	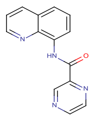	gskversion	O=C(Nc1cccc2cccnc12)c1cnccn1	9,40	BCGset	
GSK1295475A	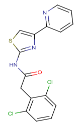	gskversion	Clc1cccc(Cl)c1CC(=O)Nc1nc(cs1)-c1ccccn1	9,40	BCGset	
GSK1777957A	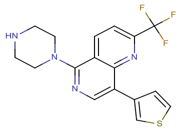	gskversion	FC(F)(F)c1ccc2c(ncc(-c3ccsc3)c2n1)N1CCNCC1	9,40	BCGset	
GSK1896394A	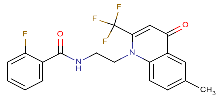	gskversion	Cc1ccc2n(CCNC(=O)c3ccccc3F)c(cc(=O)c2c1)C(F)(F)F	9,40	BCGset	
GSK2156660A	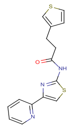	gskversion	O=C(CCc1ccsc1)Nc1nc(cs1)-c1ccccn1	9,40	BCGset	
GSK480740A	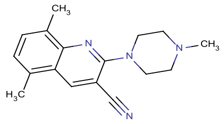	gskversion	CN1CCN(CC1)c1nc2c(C)ccc(C)c2cc1C#N	9,40	BCGset	
SB-204909-A	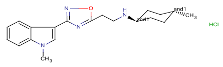	gskversion	C[C@H]1CC[C@@H](CC1)NCCc1nc(no1)-c1cn(C)c2ccccc12	9,40	BCGset	
GV187303X	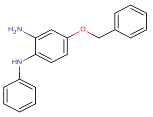	gskversion	Nc1cc(OCc2ccccc2)ccc1Nc1ccccc1	9,44	BCGset	TBset
BRL-51100AM	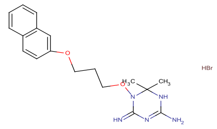	gskversion	CC1(C)NC(N)=NC(=N)N1OCCCOc1ccc2ccccc2c1	9,50	BCGset	
GSK1064445A	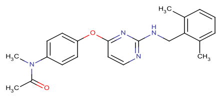	gskversion	CN(C(C)=O)c1ccc(Oc2ccnc(NCc3c(C)cccc3C)n2)cc1	9,50	BCGset	
GSK1157396A	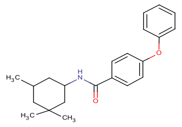	gskversion	CC1CC(CC(C)(C)C1)NC(=O)c1ccc(Oc2ccccc2)cc1	9,50	BCGset	
GSK1379183A	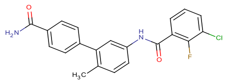	gskversion	Cc1ccc(NC(=O)c2cccc(Cl)c2F)cc1-c1ccc(cc1)C(N)=O	9,50	BCGset	
GSK1421166A	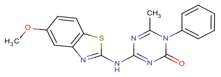	gskversion	COc1ccc2sc(Nc3nc(C)n(-c4ccccc4)c(=O)n3)nc2c1	9,50	BCGset	

GSK1729163A	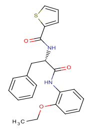	gskversion	CCOc1ccccc1NC(=O)[C@H](Cc1ccccc1)NC(=O)c1cccs1	9,50	BCGset	
GSK1788522A	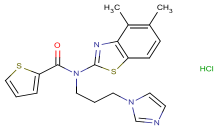	gskversion	Cc1ccc2sc(nc2c1C)N(CCCn1ccnc1)C(=O)c1cccs1	9,50	BCGset	
GSK1829705A	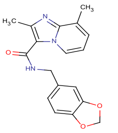	gskversion	Cc1nc2c(C)cccn2c1C(=O)NCc1ccc2OCOc2c1	9,50	BCGset	
GSK2200142A	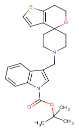	gskversion	CC(C)(C)OC(=O)n1cc(CN2CCC3(CC2)OCCc2sccc32)c2ccccc12	9,50	BCGset	
GSK520926A	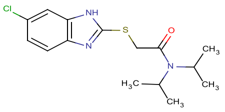	gskversion	CC(C)N(C(C)C)C(=O)CSc1nc2ccc(Cl)cc2[nH]1	9,50	BCGset	
GW351921X	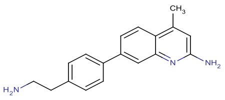	gskversion	Cc1cc(N)nc2cc(ccc12)-c1ccc(CCN)cc1	9,50	BCGset	
SB-354364	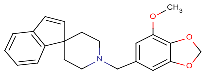	gskversion	COc1cc(CN2CCC3(CC2)C=Cc2ccccc32)cc2OCOc12	9,50	BCGset	TBset
GSK1310678A	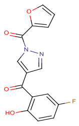	gskversion	Oc1ccc(F)cc1C(=O)c1cnn(c1)C(=O)c1ccco1	9,55	BCGset	TBset
GSK1635139A	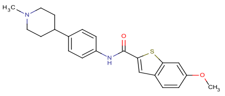	gskversion	COc1ccc2cc(sc2c1)C(=O)Nc1ccc(cc1)C1CCN(C)CC1	9,55	BCGset	TBset
GSK1073385A	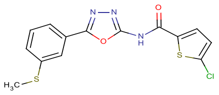	gskversion	CSc1cccc(c1)-c1nnc(NC(=O)c2ccc(Cl)s2)o1	9,60	BCGset	
GSK1199840A	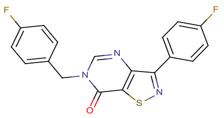	gskversion	Fc1ccc(Cn2cnc3c(nsc3c2=O)-c2ccc(F)cc2)cc1	9,60	BCGset	
GSK1789622A	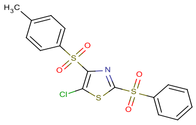	gskversion	Cc1ccc(cc1)S(=O)(=O)c1nc(sc1Cl)S(=O)(=O)c1ccccc1	9,60	BCGset	
GSK1825992A	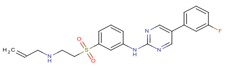	gskversion	Fc1cccc(c1)-c1cnc(Nc2cccc(c2)S(=O)(=O)CCNCC=C)nc1	9,60	BCGset	

GSK352939A	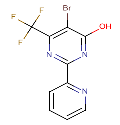	gskversion	Oc1nc(nc(c1Br)C(F)(F)F)-c1ccccn1	9,60	BCGset	
GSK787059A	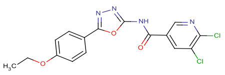	gskversion	CCOc1ccc(cc1)-c1nnc(NC(=O)c2cnc(Cl)c(Cl)c2)o1	9,60	BCGset	
SB-293648-A	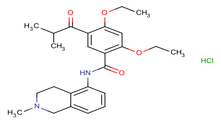	gskversion	CCOc1cc(OCC)c(cc1C(=O)Nc1cccc2CN(C)CCc12)C(=O)C(C)C	9,60	BCGset	
SB-811139-V	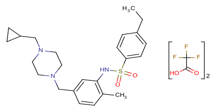	gskversion	CCc1ccc(cc1)S(=O)(=O)Nc1cc(CN2CCN(CC3CC3)CC2)ccc1C	9,60	BCGset	
GSK463878A	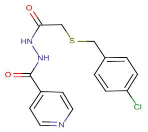	gskversion	Clc1ccc(CSCC(=O)NNC(=O)c2ccncc2)cc1	9,70	BCGset	
GSK662085A	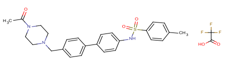	gskversion	CC(=O)N1CCN(Cc2ccc(cc2)-c2ccc(NS(=O)(=O)c3ccc(C)cc3)cc2)CC1	9,70	BCGset	
GSK1611550A	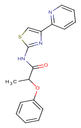	gskversion	CC(Oc1ccccc1)C(=O)Nc1nc(cs1)-c1ccccn1	9,77	BCGset	TBset
GSK133167A	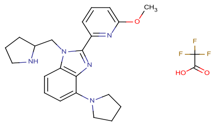	gskversion	COc1cccc(n1)-c1nc2c(cccc2n1CC1CCCN1)N1CCCC1	9,77	BCGset	TBset
GSK1174628A	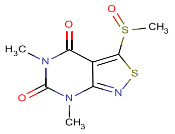	gskversion	Cn1c2nsc(S(C)=O)c2c(=O)n(C)c1=O	9,77	BCGset	TBset
GSK1352903A	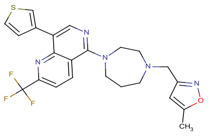	gskversion	Cc1cc(CN2CCCN(CC2)c2ncc(-c3ccsc3)c3nc(ccc23)C(F)(F)F)no1	9,80	BCGset	
GSK1473876A	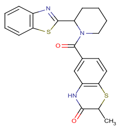	gskversion	CC1Sc2ccc(cc2NC1=O)C(=O)N1CCCCC1c1nc2ccccc2s1	9,80	BCGset	
GSK1540431A	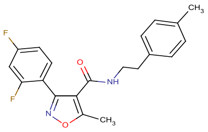	gskversion	Cc1onc(c1C(=O)NCCc1ccc(C)cc1)-c1ccc(F)cc1F	9,80	BCGset	
GSK1801530A	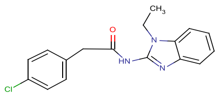	gskversion	CCn1c(NC(=O)Cc2ccc(Cl)cc2)nc2ccccc12	9,80	BCGset	

GSK581005A	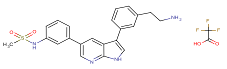	gskversion	CS(=O)(=O)Nc1cccc(c1)-c1cnc2[nH]cc(-c3cccc(CCN)c3)c2c1	9,80	BCGset	
GSK670764A	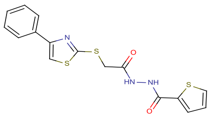	gskversion	O=C(CSc1nc(cs1)-c1ccccc1)NNC(=O)c1cccs1	9,80	BCGset	
GSK817364A	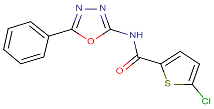	gskversion	Clc1ccc(s1)C(=O)Nc1nnc(o1)-c1ccccc1	9,80	BCGset	
GW874683X	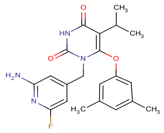	gskversion	CC(C)c1c(Oc2cc(C)cc(C)c2)n(Cc2cc(N)nc(F)c2)c(=O)[nH]c1=O	9,80	BCGset	
SB-252384-A	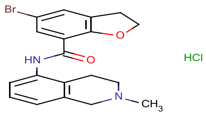	gskversion	CN1CCc2c(C1)cccc2NC(=O)c1cc(Br)cc2CCOc12	9,80	BCGset	
SB-281244	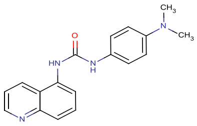	gskversion	CN(C)c1ccc(NC(=O)Nc2cccc3ncccc23)cc1	9,80	BCGset	
GSK1712788A	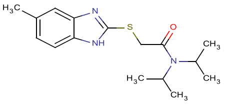	gskversion	CC(C)N(C(C)C)C(=O)CSc1nc2cc(C)ccc2[nH]1	9,90	BCGset	
GSK1829730A	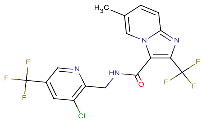	gskversion	Cc1ccc2nc(c(C(=O)NCc3ncc(cc3Cl)C(F)(F)F)n2c1)C(F)(F)F	9,90	BCGset	
GSK1900293A	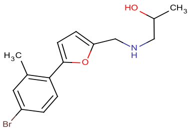	gskversion	CC(O)CNCc1ccc(o1)-c1ccc(Br)cc1C	9,90	BCGset	
GSK368523A	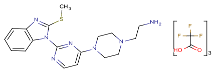	gskversion	CSc1nc2ccccc2n1-c1nccc(n1)N1CCN(CCN)CC1	9,90	BCGset	
GSK676381A	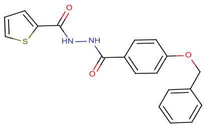	gskversion	O=C(NNC(=O)c1ccc(OCc2ccccc2)cc1)c1cccs1	9,90	BCGset	
GSK870943A	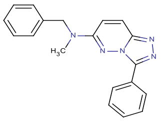	gskversion	CN(Cc1ccccc1)c1ccc2nnc(-c3ccccc3)n2n1	9,90	BCGset	
SB-461573-A	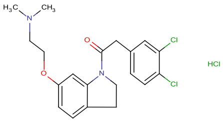	gskversion	CN(C)CCOc1ccc2CCN(C(=O)Cc3ccc(Cl)c(Cl)c3)c2c1	9,90	BCGset	

SB-509529-V	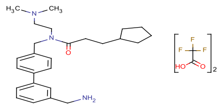	gskversion	CN(C)CCN(Cc1ccc(cc1)-c1cccc(CN)c1)C(=O)CCC1CCCC1	9,90	BCGset	
SB-747683	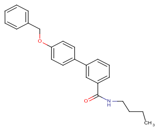	gskversion	CCCCNC(=O)c1cccc(c1)-c1ccc(OCc2ccccc2)cc1	9,90	BCGset	
SB-811154-V	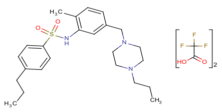	gskversion	CCCN1CCN(Cc2ccc(C)c(NS(=O)(=O)c3ccc(CCC)cc3)c2)CC1	9,90	BCGset	
GSK1106139A	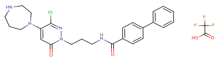	gskversion	Clc1nn(CCCNC(=O)c2ccc(cc2)-c2ccccc2)c(=O)cc1N1CCCNCC1	10,00	BCGset	
GSK1238425A	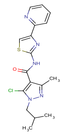	gskversion	CC(C)Cn1nc(C)c(C(=O)Nc2nc(cs2)-c2ccccn2)c1Cl	10,00	BCGset	
GSK1325216A	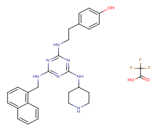	gskversion	Oc1ccc(CCNc2nc(NCc3cccc4ccccc34)nc(NC3CCNCC3)n2)cc1	10,00	BCGset	
GSK1633249A	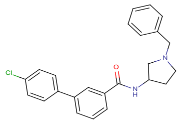	gskversion	Clc1ccc(cc1)-c1cccc(c1)C(=O)NC1CCN(Cc2ccccc2)C1	10,00	BCGset	
GSK922232A	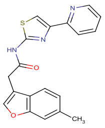	gskversion	Cc1ccc2c(CC(=O)Nc3nc(cs3)-c3ccccn3)coc2c1	10,00	BCGset	
GW558375X	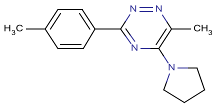	gskversion	Cc1ccc(cc1)-c1nnc(C)c(n1)N1CCCC1	10,00	BCGset	
